# AI-Driven *De Novo* Design and Development of Nontoxic DYRK1A Inhibitors

**DOI:** 10.1021/acs.jmedchem.5c00512

**Published:** 2025-05-03

**Authors:** Eduardo González García, Pablo Varas, Pedro González-Naranjo, Eugenia Ulzurrun, Guillermo Marcos-Ayuso, Concepción Pérez, Juan A. Páez, David Rios Insua, Simón Rodríguez Santana, Nuria E. Campillo

**Affiliations:** † Instituto de Ciencias Matemáticas (ICMAT-CSIC), C/Nicolás Cabrera, 13-15, 28049 Madrid, Spain; ‡ AItenea Biotech S.L., C/Alfonso XII, 46, 28014 Madrid, Spain; § Instituto de Química Médica (IQM-CSIC). C/Juan de la Cierva, 3, 28006 Madrid, Spain; ∥ Centro de Investigaciones Biológicas Margarita Salas (CIB Margarita Salas-CSIC). C/Ramiro de Maeztu, 9, 28040 Madrid, Spain; ⊥ Universidad Pontificia Comillas (ICAI) - IIT. C/Alberto Aguilera, 25, 28015 Madrid, Spain

## Abstract

Dual-specificity tyrosine-phosphorylation-regulated kinase 1A (DYRK1A) is implicated in several human diseases, including DYRK1A syndrome, cancer, and neurodegenerative disorders such as Alzheimer’s disease, making it a relevant therapeutic target. In this study, we combine artificial intelligence with traditional drug discovery methods to design nontoxic DYRK1A inhibitors. An ensemble QSAR model was used to predict binding affinities, while a directed message passing neural network evaluated toxicity. Novel compounds were generated using a hierarchical graph-based generative model and subsequently refined through molecular docking, chemical synthesis, and experimental validation. This pipeline led to the identification of pyrazolyl-1*H*-pyrrolo­[2,3-*b*]­pyridine **1** as a potent inhibitor, from which a new derivative series was developed. Enzymatic assays confirmed nanomolar DYRK1A inhibition, and additional assays demonstrated antioxidant and anti-inflammatory properties. Overall, the resulting compounds exhibit strong DYRK1A inhibition and favorable pharmacological profiles.

## Graphical abstract

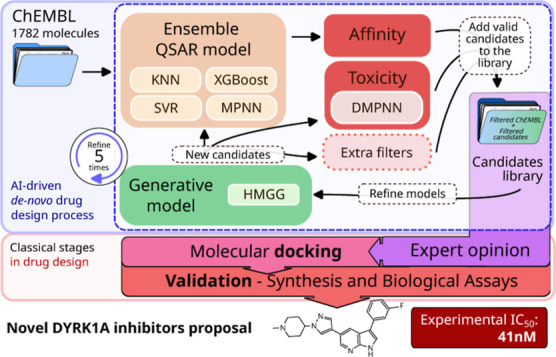

## Introduction

Drug discovery, particularly when aiming to address complex diseases like Alzheimer’s disease (AD) or cancer, relies on a deep understanding of biological mechanisms and identifying potential therapeutic targets. Among these, the dual-specificity tyrosine-phosphorylation-regulated kinase 1A (DYRK1A) family is notable for its role in supporting fundamental biological processes and its association with important diseases, including DYRK1A syndrome, cancer, diabetes,^1,2^ and neurodegenerative disorders such as AD.^2,3^ This makes DYRK1A an exciting and promising target to simultaneously tackle multiple different diseases.

The design of new molecules against a target has traditionally been addressed using classic design methods, which face the significant and complex challenges derived from navigating the vast chemical space to identify compounds that meet desired properties. In recent years, artificial intelligence (AI) has emerged as a promising methodology providing tools for *de novo* molecule generation, leveraging extensive databases in conjunction with novel AI generative methods.^4–6^ By integrating these approaches, researchers aim to accelerate the discovery of novel therapeutics. Numerous successful candidates have emerged from similar strategies, garnering widespread interest in the machine learning, statistics, and chemical design communities.^6–10^ This interest has been most pronounced in instances with abundant data, while successful cases based on limited data sets remain comparatively rare. Furthermore, most studies focus on proposing promising molecule candidates based on computational models, with far fewer extending to full in vitro validation with synthesis and laboratory measurement of molecular properties. This limitation is understandable, as such efforts require significant resources, including access to qualified experts and well-equipped laboratories.

This paper demonstrates a successful integration of AI-based techniques for *de novo* molecule generation to design DYRK1A inhibitors. Our generative pipeline is validated through experimental in vitro studies of candidate molecules proposed to inhibit the DYRK1A enzyme, a promising therapeutic target for different diseases. Conducted in a small-data regime, this process employed a range of AI techniques to develop a robust model for suitable candidate generation. The main candidate identified was synthesized alongside its derivatives to evaluate their biological activity. Enzymatic inhibition, anti-inflammatory effects, and antioxidant capacity were experimentally confirmed, showcasing highly promising results.

## AI *De Novo* Molecular Design

Drug development is one of the most complex industrial processes, typically taking 10–12 years and costing over a trillion dollars. Around 90% of proposed compounds fail to gain FDA approval.^11^ Preclinical testing also relies on increasing animal use, raising ethical concerns while remaining a necessary step. The challenge lies in navigating a vast chemical space (10^60^) molecules to identify viable drug candidates.^12^ Efforts to accelerate this process have focused on reducing time, costs, and animal testing.^13,14^ AI has emerged as a promising tool, facilitating and improving various aspects of drug discovery.^10,15^

A key application of AI in drug development is *de novo* molecular design, which generates novel molecules with optimized properties. Recent advances in AI-driven molecular generation have led to diverse approaches tailored to various drug discovery tasks. Broadly speaking, these methods can be mostly categorized into two main types:

1. QSAR models estimate compound properties using chemical database information, using *regression* for continuous values (e.g., affinity, log *P*, QED) or *classification* for discrete labels (e.g., toxic vs nontoxic). Their performance depends on data quantity and quality. While neural networks^16^ require large data sets, public databases and frameworks such as Chemprop^17^ (used in this work) help mitigate these limitations, enabling the use of advanced predictive techniques.
2. Generative AI models create new data drawing on existing data sets, extrapolating from data or expert knowledge to propose novel compounds in *de novo* design. These approaches can be *data-heavy* (e.g., deep learning) or *data-sparse* (e.g., evolutionary methods). We focus on multitarget generation using methods that extract insights from small data sets. While integrating predictive and generative models is effective, our limited DYRK1A inhibition data required treating both tasks separately. To address this, we fine-tuned state-of-the-art models with minimal computational resources to efficiently design molecular candidates.

## Related Work in AI *De Novo* Design

While the concepts behind computer-assisted *de novo* molecular design have long been established,^18,19^ recent advances have driven unprecedented improvements in the field. In particular, AI-based methods are increasingly being applied to accelerate and optimize drug design processes.^20^ Seminal works on generative models, such as Gómez-Bombarelli et al., have paved the way for future developments, significantly influencing advancements in QSAR model formulation.^21^

Given the rapid expansion of research in this area, an exhaustive list of references is beyond the scope of this paper and is better suited for a comprehensive review, such as that by Pang et al.^22^ Their work highlights the efficiency of deep generative models in producing drug-like molecules with tailored properties. Li et al.^23^ extended these ideas by introducing a 3D deep generative model capable of designing molecules that fit specific target binding sites, successfully applying it to inhibitors of the SARS-CoV-2 main protease.

Earlier studies^18^ emphasized the need to consider synthetic feasibility in molecular design, a challenge increasingly addressed by AI-driven models incorporating synthetic accessibility constraints. More recent works^24–26^ have demonstrated AI’s potential to accelerate drug discovery by refining molecular generation and optimizing multiple pharmacological properties. Collectively, these advances mark a shift toward generative models that are highly adaptable to real-world challenges.

On the experimental side, a 2019 study used deep learning to identify inhibitors of the discoidin domain receptor 1 kinase (DDR1).^24^ By leveraging biological activity data and molecular structures, the study predicted candidate molecules with DDR1 inhibitory activity, which were subsequently validated through molecular docking to assess their binding affinity and structural compatibility. This AI-driven approach significantly accelerated the discovery of DDR1-targeting therapeutics.

Building on this methodology, our work harnesses AI tools—including predictive models and generative algorithms—to design nontoxic DYRK1A inhibitors. We integrate our DYRK1A affinity data set with state-of-the-art approaches, generating structurally novel molecules that undergo comprehensive validation, including protein–ligand docking simulations. After synthesis, these compounds are further evaluated through enzymatic and cellular assays, ultimately leading to a family of DYRK1A inhibitors with a strong drug-like profile. Such extensive validation is uncommon in similar studies, underscoring the robustness of our approach.

## Results and Discussion

The main focus of this work is the design of DYRK1A inhibitors using AI tools and subsequent experimental verification. Following the computational design phase, we synthesize and biologically evaluate the performance of each compound to determine its suitability for this task. This evaluation includes both *in vitro* and cellular assays for the most promising molecules. To approach this systematically, we propose the protocol detailed in Figure 1, which outlines the complete process and involves two complementary strategies: AI *de novo* design and classical drug development.

**Figure 1:**
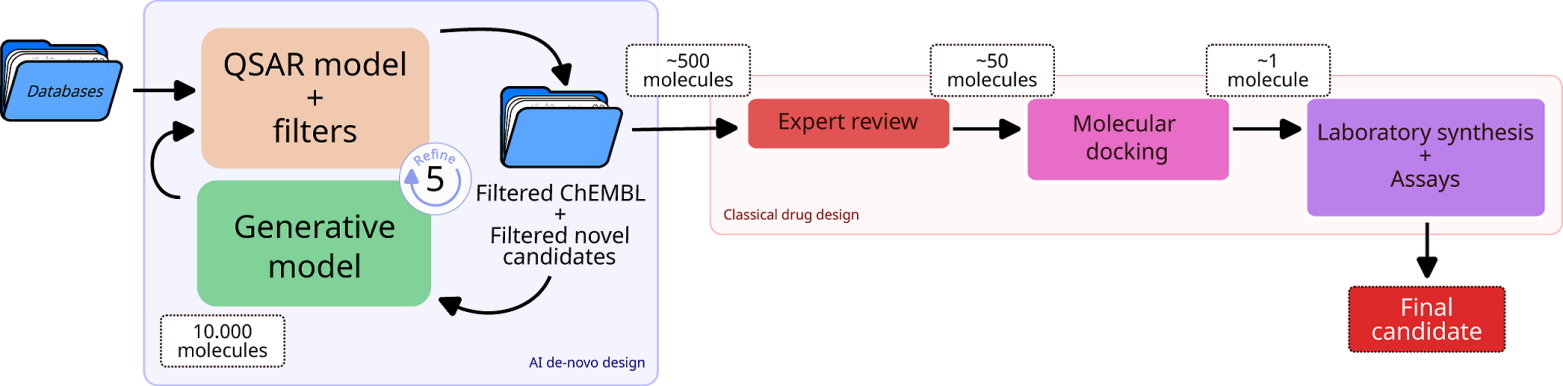
Protocol for DYRK1A inhibitors development.

Following Figure 1, utilizing our DYRK1A affinity data set, obtained from the ChEMBL database,^27^ we constructed and fitted QSAR predictive models to estimate the properties of different compounds. By combining this with other public data sets and available models, we also predicted additional chemical properties for proposed compounds with unknown affinity levels, such as their potential toxicity. Similarity and internal consistency filters were applied to ensure that the proposed molecules were synthesizable in the laboratory and distinct from those in pre-existing databases. This entire process was coupled with a generative model, which was iteratively refined using the curated selection of candidates to thoroughly explore the most relevant part of the chemical space. Please refer to the Experimental Section for additional experimental details. Next, thousands (10^4^) of candidates were generated using our generative models and filtered based on predictions of binding affinity, toxicity, and similarity to known inhibitors. Approximately the top 5% of the most promising candidate molecules were retained through this part of the pipeline. Following a review by expert chemists, the top candidates underwent molecular docking studies to rank their potential. Finally, the highest-ranked molecules were handpicked, synthesized, and tested in the laboratory.

### AI-Assisted *De Novo* Design of DYRK1A Inhibitors

#### QSAR Models for DYRK1A Inhibitors

As mentioned earlier, the first step in our denovo molecule design process involves training a model capable of assessing the quality of molecules based on two primary objectives: DYRK1A inhibition, measured through their binding affinity, and nontoxicity. The resulting QSAR model will be key component of the later generative effort, as it enables more efficient navigation through the chemical space, guiding the search toward the most promising candidate regions. This approach estimates these key properties without requiring each proposed compound to be explicitly present in the database. The process was conducted under a low-data regime, as the available database of affinity values was not large. While this limitation poses challenges for certain techniques, we view it as a significant point of interest, as successfully conducting molecule generation in such a context could pave the way for similar efforts in other low-data scenarios.

As a measure for the affinity, we employ several approaches trained on the primary DYRK1A database. The values for pChEMBL present in this database are derived from four different measurement methods (*K*_i_, *K*_d_, IC_50_ and EC_50_). This introduces observable differences between the recorded pChEMBL values solely caused by the measurement selected, as illustrated in Figure 2. In this context, we conduct separate standardization and normalization for different measurements (for further details, please see the Experimental Section).

**Figure 2:**
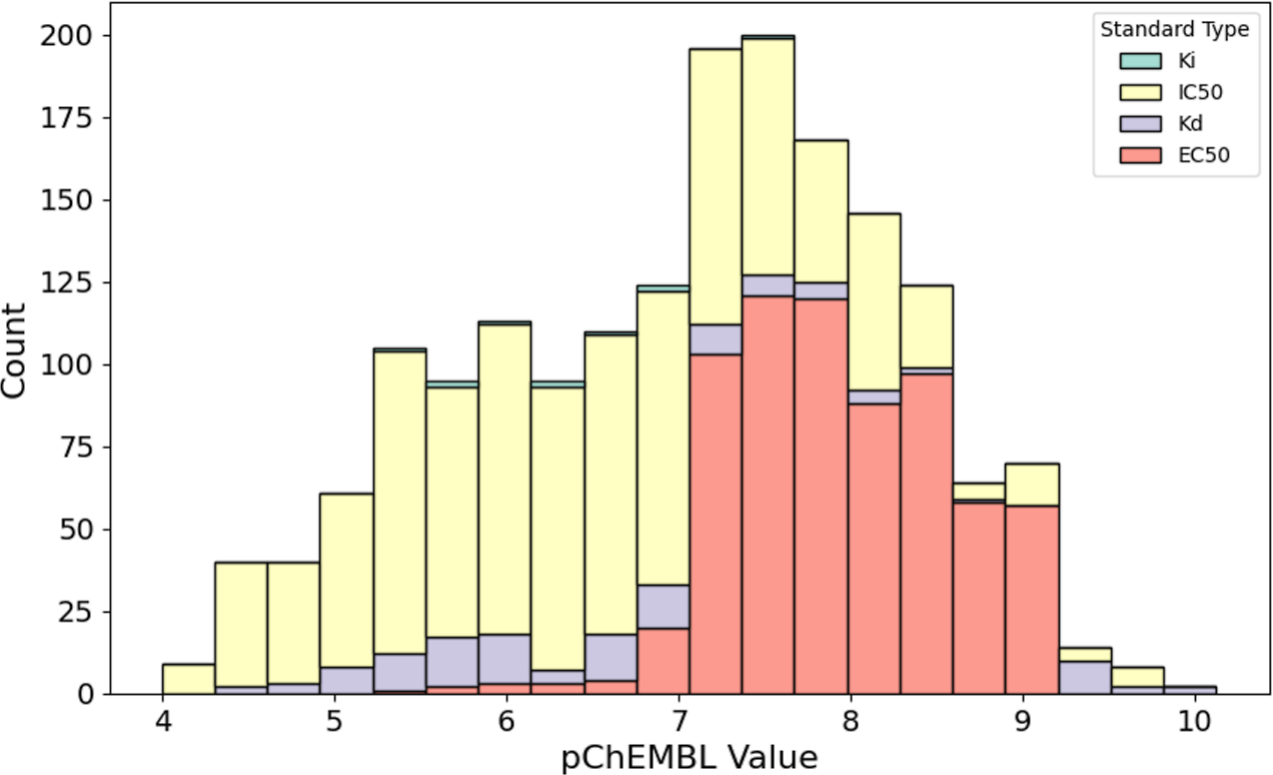
Stacked-bars plot of the frequencies of DYRK1A inhibitors affinity featured in the database by measurement type.

To predict the affinity of each compound, we designed an ensemble model comprising XGBoost,^28^ support vector regressors (SVR), k-nearest neighbors (KNN), and a directed message-passing neural network (DMPNN),^17^ following extensive experimental evaluation. While each model performed well individually, integrating these methods into an ensemble predictor provided more robust and reliable forecasts. This ensemble model was compared with other predictive models, including MolCLR,^29^ a graph neural network (GNN) pretrained using contrastive learning; subgraph pattern GNn (SPGNN),^30^ another GNN pretrained with a combination of self-supervised and multitask supervised tasks; and traditional models such as random forests (RF), Gaussian processes (GP), and a multilayer perceptron (MLP). For further details about the models and molecular representations used, please refer to the section on Experimental Section.

Figure 3 presents the performance of all models evaluated using four key metrics: root mean squared error (RMSE), mean absolute error (MAE), explained variance score (EVS), and coefficient of determination (*R* ^2^). As illustrated, the ensemble consistently delivered superior median performance across all metrics, with minimal variance across the data set. Based on these results, this ensemble was chosen as the QSAR model for this study, followed by a cross-validation procedure to optimize the combination of hyperparameters and molecular representations specific to each model. In this implementation, we maintained homogeneous weights for all the ensemble’s models. However, further generalizations of this approach could employ a weighted average for the distinct model’s predictions that constitute the ensemble, potentially achieving even higher performance.

**Figure 3:**
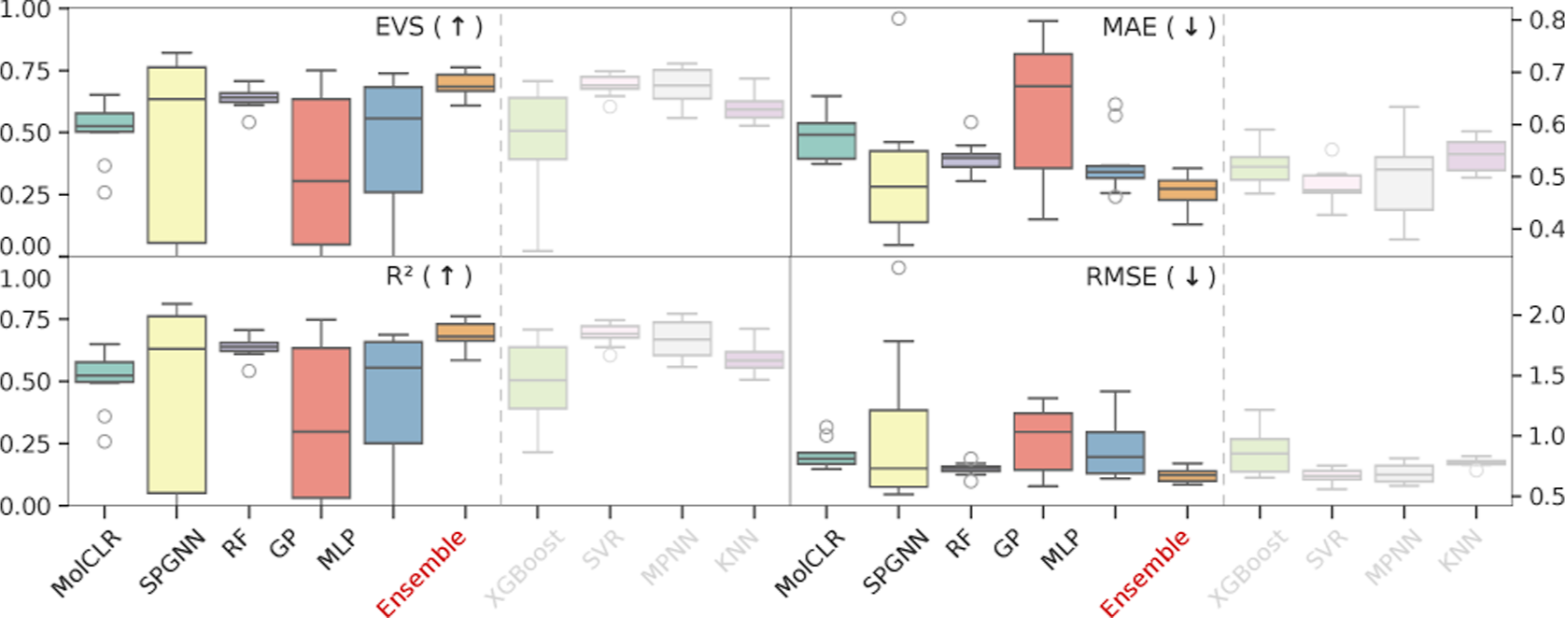
Metric-wise results for each method. Each subfigure title specifies the metric and whether it should be maximized (↑) or minimized (↓). Individual models that constitute the ensemble are shown to the right of the dashed lines, with a faded appearance for distinction. In general, the ensemble showcases the best performances overall.

Figure 4 provides additional insights by summarizing the methods through their mean rankings and corresponding standard deviations, offering a clearer comparison suggesting the superiority of the ensemble model. Rankings were constructed by sorting the models based on their predictive performance for each evaluation metric.

**Figure 4:**
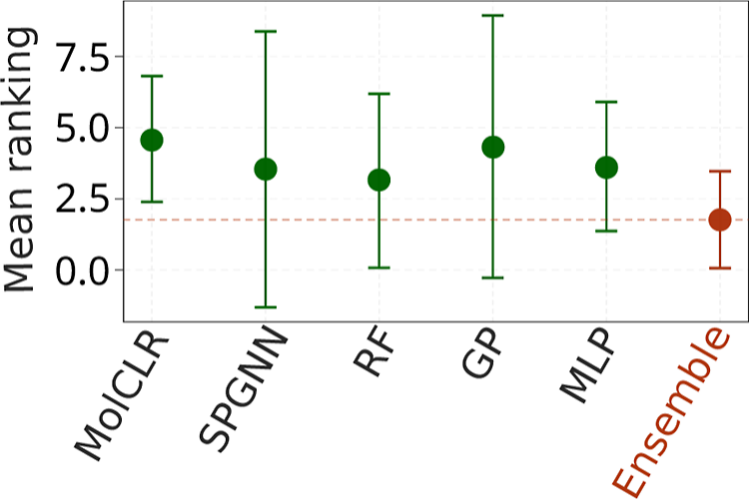
Performance ranking of models. The selected ensemble is highlighted in red (lower is better).

To evaluate the toxicity of the identified compounds, we employed the directed MPNN from the Chemprop package.^17^ This state-of-the-art model outputs 12 values, each representing the probability of a compound belonging to a specific toxicity class. The model was utilized both as a predictive tool and a filtering mechanism, requiring each candidate molecule to be classified as nontoxic across all 12 metrics to qualify as nontoxic.

Finally, several filters were employed during the generative process to classify potential candidate molecules as promising alongside the predictive affinity and toxicity models. In particular, we included filters to ensure a low similarity of generated molecules to those in pre-existing data sets, as well as a consistency check applied to the predictions of the QSAR ensemble model to improve the robustness of the whole system. Specifically, if the predictions from each ensemble’s models differed by more than a specified threshold, the proposed compound was discarded. This ensured alignment among the models regarding the predicted affinity value of promising compounds. This heuristic provided greater stability during the subsequent generative phases. For further details, please refer to the description of the screening filters in the Experimental Section.

#### Generative Models and *De Novo* Design

For the generative process, we explored several models to generate potential DYRK1A inhibitors, with the models selected depending on the data availability and chemical viability of the proposed compounds. In all cases, candidate molecules were rigorously filtered according to our criteria for binding affinity, toxicity, and novelty.

The chosen model was the HGG model,^31^ trained on a data set of DYRK1A inhibitors. Through an iterative process, it produced five batches of 10^4^ new molecules. After each generation, the new molecules were evaluated for binding affinity, toxicity, and structural similarity to known inhibitors. Molecules passing these filters were added back to the training set, and the model was retrained, enabling iterative refinement of the candidate list over five cycles. We restrict the usage of this recursive approach to a few iterations (≤5) to avoid convergence, which may induce a decrease in the diversity of the outputs after excessive iterations. Empirically, this process ensured a final set of molecules with high affinity, low toxicity, and high chemical novelty, resulting in a robust selection of viable inhibitors. The model consistently generated compounds that met these criteria and demonstrated structural characteristics aligned with known effective compounds. Expert chemists reviewing these structures deemed them promising in terms of chemical properties and drug-likeness. Subsequent experimental results confirmed these initial assessments, further validating the effectiveness of the HGG approach. For additional experimental details, as well as information on other models evaluated during this step, please refer to the Experimental Section.

Finally, the top candidates generated by the HGG model underwent conventional molecular docking calculations to check their experimental performance.

#### Molecular Docking of Top Designed Compounds

A structure-based virtual screening (SBVS) was performed for ∼50 novel inhibitors targeting the ATP-binding zone of the DYRK1A protein. As control the 4E3 (5t) compound was redocked into the ATP binding site of the chain A of the published DYRK1A structure (PDB code 4YLL),^32^ reproducing the original pose as the highest ranked posed (rmsd value of 0.715 Å, see Experimental Section). Table 1 shows the top compounds (rank I-IX) obtained from virtual screening, all of which score higher than the control (4E3). As additional information, Table S1 (see Supporting Information) shows the top 50 molecules resulting from the docking studies.

**Table 1:**
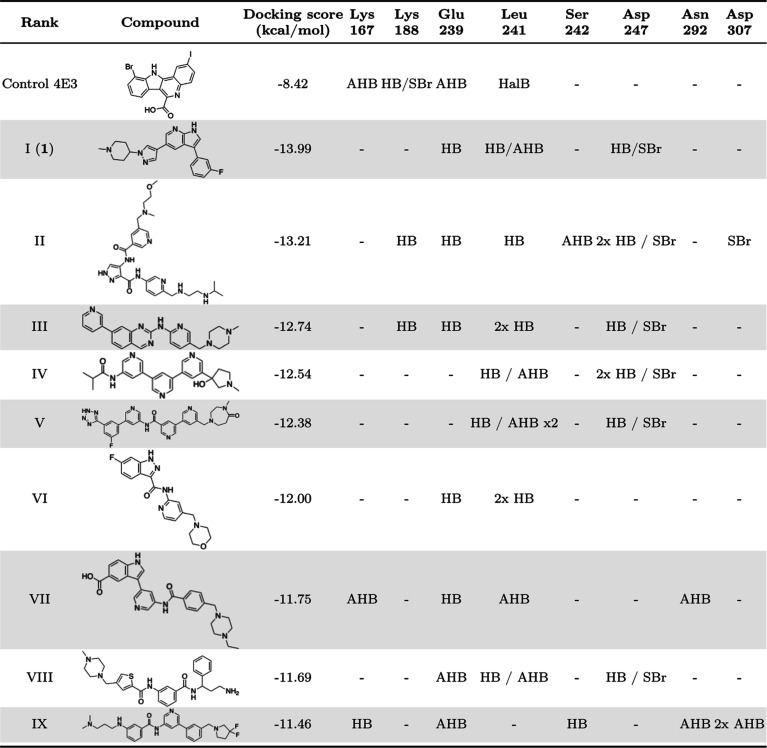
Interactions of the Top-10 Ranked Compounds with Residues in the Binding Site of DYRK1A according to Theoretical Molecular Docking Studies^a^

a AHB: aromatic hydrogen bond; HB: hydrogen bond; SBr: salt bridge; HalB: halogen bond.

The inhibitor 4E3 forms bonds with Lys167, Lys188, Glu239, and Leu241 (Table 1). Remarkably, interactions with catalytic residues Glu239 and Leu241 are present in 67% of the compounds I-IX, suggesting their crucial role in ligand binding typical for the other inhibitors.^33^ On the other hand, only compounds II and III interact with the catalytic lysine Lys188. However, the remaining seven compounds have an arene oriented toward this catalytic residue.

Significantly, the key hinge interactions are preserved across several newly synthesized compounds. The conserved hinge motif in protein kinases, comprising three amino acids, is defined by their positions relative to the downstream sequence of the “gatekeeper” residue, designated as gk + 1, gk + 2, and gk + 3. This motif is well-known for its role in forming traditional hydrogen bonds with inhibitors.^34^ In our case, the reference compound does not exhibit these hydrogen bonds. In contrast, the novel inhibitors I, III, and VI interact with the hinge backbone residues Glu239 (gatekeeper + 1) and Leu241 (gatekeeper + 3) (Table 1). Particularly, the ligand’s chemical moieties interact with the hinge region through three key hydrogen bonds, the ligand donates an H-bond to the main-chain carbonyl of gk + 1, while a nitrogen atom accepts an H-bond from the main-chain amide of gk + 3. The third interaction occurs when the ligand donates a proton to the main-chain carbonyl of gk + 3. Compounds I and III exhibit interactions with the hinge backbone similar to those observed in the adenine moiety of ATP within the ATP-binding pocket, involving both canonical and noncanonical hydrogen bonds (exemplified in Figure 5). Although compound VI also forms hydrogen bonds with gk + 1 (Glu239) and gk + 3 (Leu241), these interactions are exclusively canonical (Table 1). In contrast, none of the remaining compounds exhibit these three specific interactions with the hinge region.

**Figure 5:**
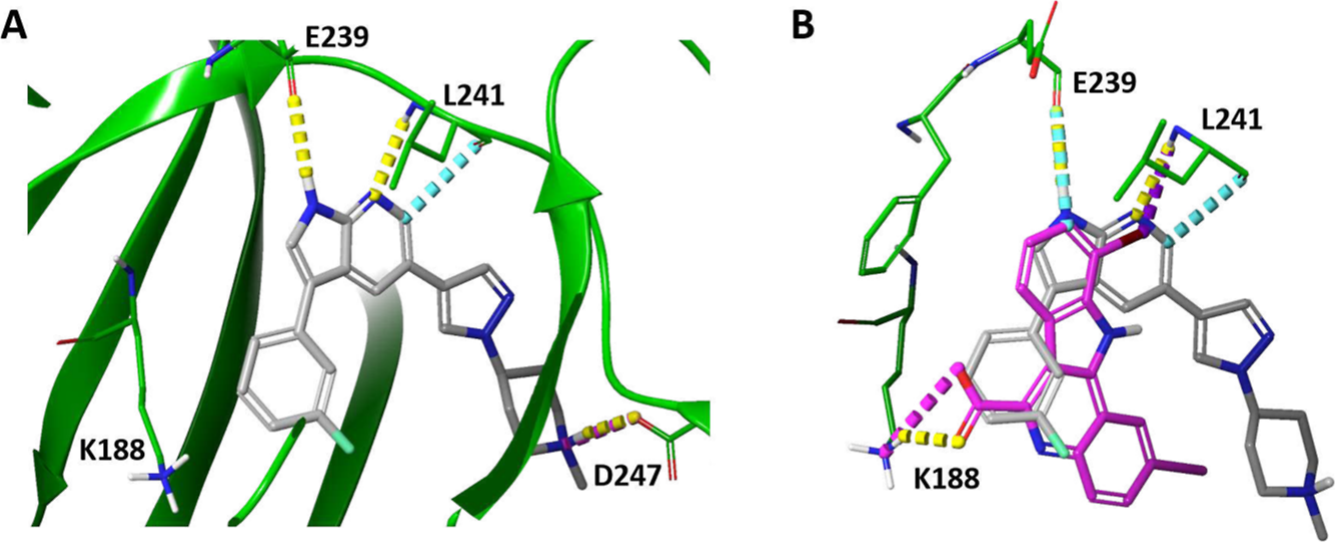
Interactions of residues gk + 1 (E239) and gk + 3 (L241) in the ATP binding site of DYRK1A structure (PDB 4YLL) with inhibitors. (A) Hit compound I. (B) Superposition of the reference compound 4E3 (magenta) and the top hit compound I (gray).

The identification of both canonical and noncanonical hydrogen bonds aligns with the established mechanism through which inhibitor scaffolds mimic adenine’s interaction with the hinge. These scaffolds incorporate hydrogen bond donors and acceptors that engage with the carbonyl groups of gk + 1 and gk + 3.^35,36^ Considering both the docking score and the hinge hydrogen bond interactions, 3-(3-fluorophenyl)-5-(1-(1-methylpiperidin-4-yl)-1*H*-pyrazol-4-yl)-1*H*-pyrrolo­[2,3-*b*]­pyridine (**1**) was selected as the top candidate for synthesis and subsequent biological assays.

## Experimental Development and Validation

### Synthesis of the New Designed Compounds

The 3-(3-fluorophenyl)-5-(1-(1-methylpiperidin-4-yl)-1*H*pyrazol-4-yl)-1*H*-pyrrolo­[2,3-*b*]­pyridine (**1**) was selected as the candidate for synthesis (Figure 6). A literature search for 5-(4-piperidinyl-1*H*-pyrazolyl)-1*H*-pyrrolo­[2,3-*b*]­pyridine derivatives yielded only three papers^37–39^ and several patents^40–45^ related to this family of candidates. Based on these references, a general synthetic procedure was proposed (Scheme 1).

**Figure 6:**
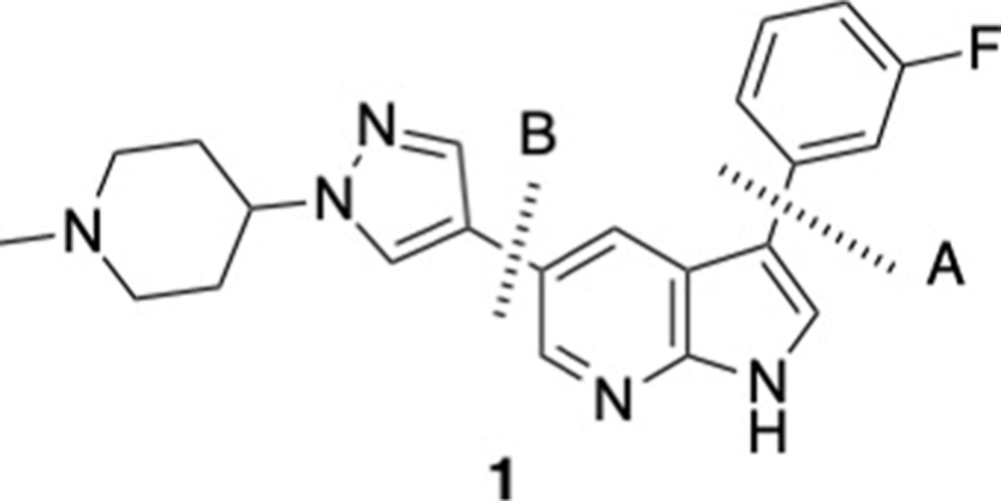
Retrosynthesis to access compound **1**.

**Scheme 1:**
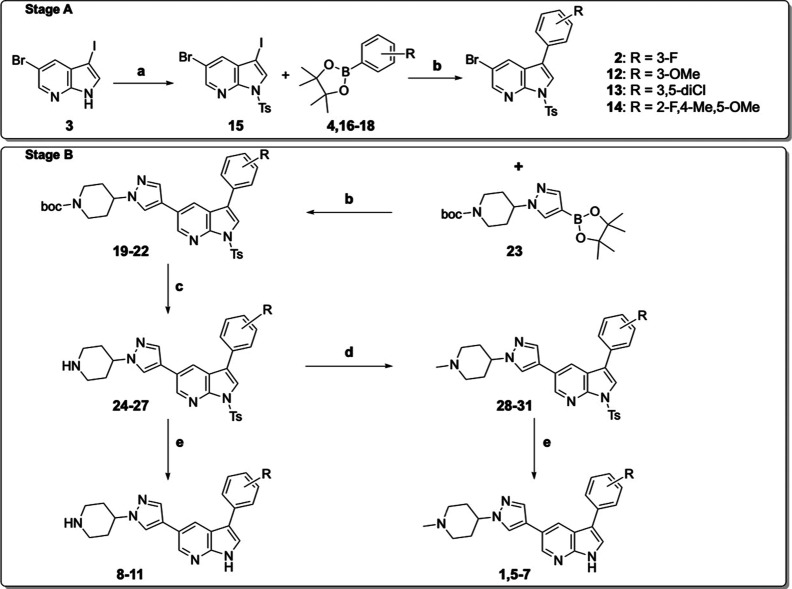
General Synthetic Route for the Preparation of 3-Arylpyrrolo­[2,3-*b*]­pyridine Derivatives **2**, **12–14** (Stage A) and Selected 1-Methylpiperidin-4-yl-1*H*-pyrazol-4-yl-pyrrolo­[2,3-*b*]­pyridine Derivatives **1**, **5–7**, and Corresponding Demethilated **8–11** (Stage B)

The synthetic methodology for obtaining the target compound involves two stages. The first one comprises the formation of 3-fluorophenyl-1*H*-pyrrolo­[2,3-*b*]­pyridine (stage A; Scheme 1), followed by the second stage (B), which introduces the 1-(1-methylpiperidin-4-yl)-1*H*-pyrazol-4-yl group (also included in Scheme 1).

Stage A represents a structurally versatile and general synthetic route for the formation of 3-fluorophenyl-1*H*-pyrrolo­[2,3-*b*]­pyridine (**2**). Initial attempts to synthesize compound **2** directly from compound **3** without protection were unsuccessful. Consequently, the preparation of **2** was accomplished via through a two-step route: first, protecting the N-1 position of the pyrrole ring in compound **3** to yield intermediate **15** (step a), followed by the introduction of the aryl substituent at position 3 using 2-(3-fluorophenyl)-4,4,5,5-tetramethyl-1,3,2-dioxaborolane **4** (Scheme 1).

The second stage (B) involves the preparation of 3-fluorophenyl-1*H*-pyrazol-4-yl-1*H*-pyrrolo­[2,3-*b*]­pyridine **1** through four-steps. This sequence includes the initial introduction of the piperidinepyrazolyl group at position 5 of the pyrrolo­[2,3-*b*]­pyridine ring, followed by methylation of the nitrogen in the piperidinyl group, as outlined in Scheme 1.

After successfully synthesizing compound **1**, a virtual library of 229 potential derivatives was proposed (see Table S2 in the Supporting Information) to predict their affinity for DYRK1A using our AI-based QSAR predictive model previously discussed. This virtual library was designed based on two key criteria: (i) structural variability of substituents on the phenyl ring and (ii) synthetic accessibility during step b (stage A; Scheme 1) using commercially available 4,4,5,5-tetramethyl-1,3,2-dioxaborolanes with different phenyl substituted groups at position 2. Consequently, the aryl substituted groups at position 3 of the pyrrolo­[2,3-*b*]­pyridine ring consisted of phenyl groups bearing one to three substituents selected from fluoro, chloro, methyl, trifluoromethyl, methoxy, nitro, and dimethylamino.

The activities of this virtual chemical library were predicted using the AI-based QSAR models described above. Most of the proposed derivatives exhibited affinity at the micro- or sub-μM level (see Table S2 in the Supporting Information). Analyzing the predicted activity of this virtual library, it was observed that donor substituents (–OMe, –Me in para, for example) presented a slightly lower activity. Thus, electron-withdrawing mono-, di-, and trisubstituted phenyl derivatives with good scores were chosen. Therefore, along with the initial compound **1**, a representative set of 3-aryl-5-pyrazolyl-1*H*-pyrrolo­[2,3-*b*]­pyridine derivatives (**5–7**) were proposed as potential candidates. Additionally, the corresponding demethylated precursors (**8–11**) were evaluated as potential candidates (Figure 7).

**Figure 7:**
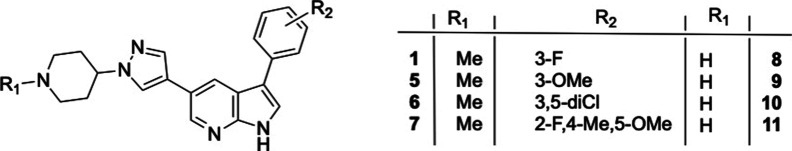
Representative set of 3-aryl-5-pyrazolyl-1*H*-pyrrolo­[2,3-*b*]­pyridine derivatives proposed.

According to stage A (Scheme 1), the first step involves the protection of position N-1 of 5-bromo-3-iodo-1*H*-pyrrolo­[2,3-*b*]­pyridine (**3**) with tosyl chloride. Subsequently, the synthesis of 3-aryl-1*H*-pyrrolo­[2,3-*b*]­pyridine derivatives (**2** and **12–14**) was performed by reacting 1-tosyl-5-bromo-3-iodo-1*H*-pyrrolo­[2,3-*b*]­pyridine (**15**) with the corresponding 2-aryl-4,4,5,5-tetramethyl-1,3,2-dioxaborolane derivatives (**16–18**), including 3-methoxy, 3,5-dichloro, or 2-fluoro-4-methyl-5-methoxy phenyl substituents, using Pd­(dppf)­Cl_2_ as catalyst.

The general synthetic route for the introduction of the 1-(1-methylpiperidin-4-yl)-1*H*-pyrazol-4-yl group from **2**, **12–14** comprises four steps (stage B; Scheme 1). The synthesis of 3-aryl-5-pyrazolyl-1*H*-pyrrolo­[2,3-*b*]­pyridine derivatives (**19–22**) was carried out by reaction 5-bromo-1-tosyl-1*H*-pyrrolo­[2,3-*b*]­pyridine derivatives (**2**, **12–14**) with *tert*-butyl 4-(4-(4,4,5,5-tetramethyl-1,3,2-dioxaborolan-2-yl)-1*H*-pyrazol-1-yl)­piperidine-1-carboxylate (**23**), using Pd­(dppf)­Cl_2_ as a catalyst. The removal of the Boc group then afforded the corresponding NH-piperidino derivatives (**24–27**). The methylpiperazinylpyrrolo­[2,3-*b*]­pyridines (**28–31**) were prepared from **24–27** by reacting with formaldehyde in formic acid. Finally, deprotection of the *N*-1-tosyl group yielded the selected candidates (**1**, **5–7**).

Last, the preparation of the corresponding NH-piperidinyl derivatives (**8–11**) was achieved by deprotection of the *N*-1-tosyl group from the piperidin-4-yl-1*H*-pyrazol-4-yl-pyrrolo­[2,3-*b*]­pyridines (**24–27**).

The structures of all newly synthesized compounds were confirmed based on their analytical and spectroscopic data. Detailed spectroscopic characterization is provided in the Supporting Information. Specifically, ^1^H NMR and ^13^C NMR chemical shifts are summarized in Tables S3 and S4, with Figures S1–S16 presenting the corresponding spectra in the Supporting Information.

### Biological Assays

Once derivatives **1**, **5–11** were synthesized, their biological evaluation was conducted. The results (see Table 2) indicate that compound **1**, designed using AI tools, exhibits activity at the nanomolar level. Additionally, the derivatives **5–7**, proposed and predicted by AI-QSAR models, and the corresponding demethylated also demonstrate significant activity. Notably, compounds **1** and **5**, along with their demethylated analogues **8** and **9**, demonstrated IC_50_ values comparable to the reference compound, harmine. Overall, there was no significant difference in IC_50_ values between the methylated compounds and their demethylated analogues. In addition, the activity against DYRK1B was also evaluated. It was observed that in general they are slightly less active, but without a significant difference. The higher activity observed for DYRK1A is expected, as the model was fine-tuned specifically for this target, while activity against DYRK1B arose naturally from the design process. In principle, we could generate compounds with reduced DYRK1B activity by explicitly incorporating this criterion into the design pipeline—for instance, by applying a DYRK1B filter that discourages the selection of molecules active against it while preserving strong affinity for DYRK1A.

**Table 2:** Inhibition of DYRK1A (IC_50_, nM), DYRK1B, and Oxygen Radical Absorbance Capacity (ORAC, Trolox Equivalents) of Compounds **1**, **5–11**

compd	R1	R2	IC_50_ (nM)^a^ DYRK1A	IC_50_ (nM)^a^ DYRK1B	ORAC^b^
1	3-F	Me	41 ± 3	78 ± 3	1.02 ± 0.03
5	3-OMe	Me	79 ± 5	119 ± 11	1.25 ± 0.07
6	3,5-diCl	Me	459 ± 24	577 ± 49	0.44 ± 0.06
7	2-F, 4-Me, 5-OMe	Me	231 ± 20	174 ± 31	1.3 ± 0.1
8	3-F	H	48 ± 3	70 ± 5	1.0 ± 0.1
9	3-OMe	H	81 ± 4	109 ± 8	1.3 ± 0.1
10	3,5-diCl	H	450 ± 18	566 ± 61	0.55 ± 0.07
11	2-F, 4-Me, 5-OMe	H	165 ± 17	165 ± 30	1.2 ± 0.1
harmine			92 ± 10	147 ± 54	

a Compounds were evaluated using ATP (10 μmol/well) and DYRKtidE (4 μmol/well) as substrate. Experiments were performed in triplicate.

b Data are expressed as μmol of trolox equivalents/μmol of tested compound.

Regarding the antioxidant capacity of compounds **1**, **5–11**, the ORAC assay revealed values around 1 trolox equivalent for most compounds, except for the dichlorinated derivatives **6** and **10**, which showed values around 0.5 trolox equivalents. As with the inhibitory activity, no notable differences were observed between the methylated compounds and their demethylated analogues (Table 2).

The difference in activity (IC_50_ and ORAC) between the methylated derivatives and the corresponding NH derivatives is not significant. This lack of difference is also observed in the docking studies, since this group (NH and NMe) does not present significant interactions.

In addition, an interesting property closely related to AD is the one that refers to the anti-inflammatory capacity of a drug. Thus, the compounds were studied using LPS-induced proinflammatory responses in BV2 microglial cells. First, the toxicity of the compounds was assessed via an MTT assay. Compounds **6**, **7**, **10**, and **11** were found to be nontoxic at concentrations up to 10 μM, whereas compounds **1**, **5**, **8**, and **9** exhibited toxicity at 10 μM but were nontoxic at 5 μM.

Subsequently, the effect of the compounds on LPS-induced proinflammatory responses in BV2 microglial cells was investigated. As Figure 8 illustrates, all compounds reduced LPS-induced NO production, with compounds **1** and **8** showing the most pronounced effects. The inhibition was dose-dependent and was particularly strong for compounds **1** and **5**, along with their demethylated analogues **8** and **9** (Figures S17 and S18 in the Supporting Information). These findings align with the high DYRK1A inhibition observed previously for these compounds.

**Figure 8:**
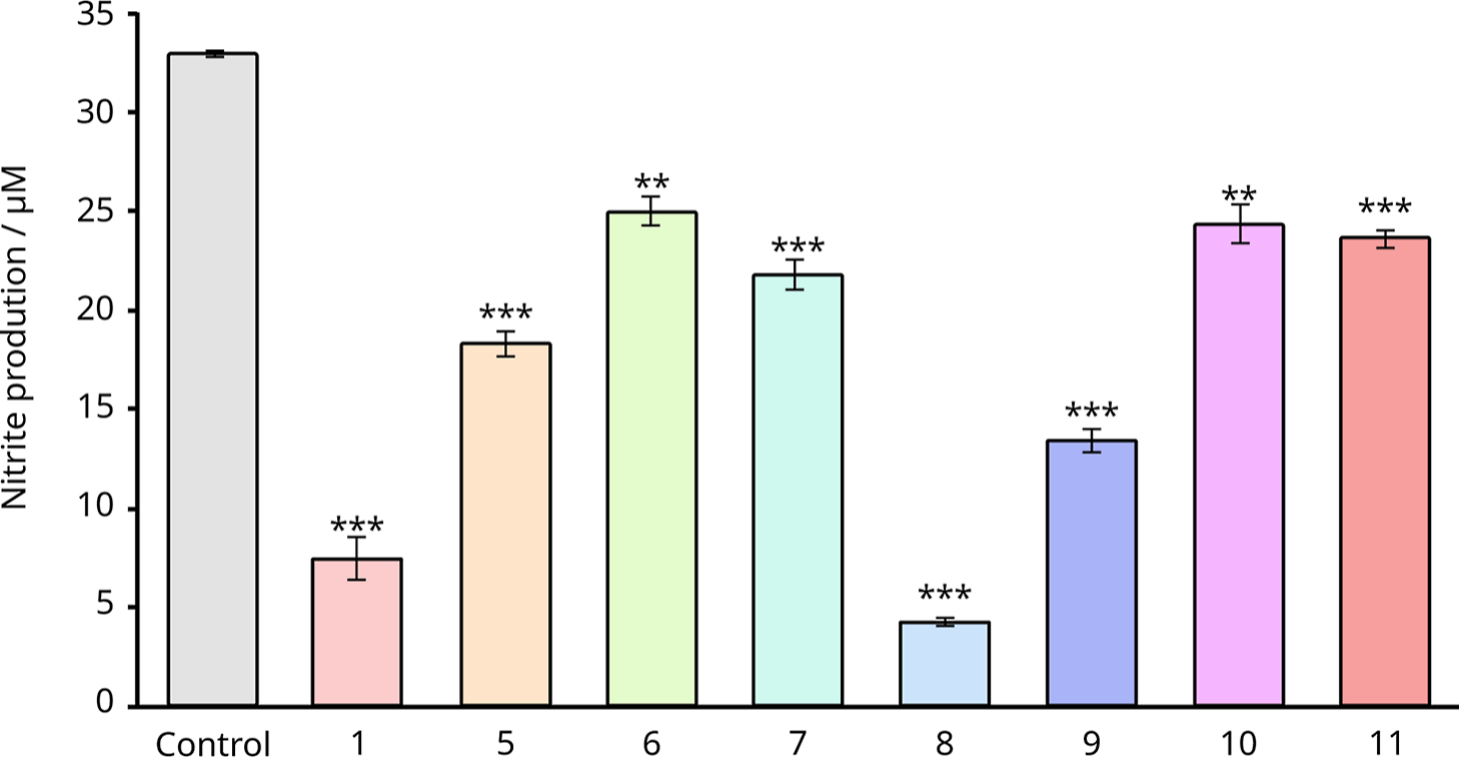
Antiinflamatory effect of compounds **1** and **5–11** at their maximum dose assayed 5 μM (**1**, **5**, **8** and **9**) or 10 μM (**6**, **7**, **10** and **11**). BV2 cells were incubated for 24 h with lipopolysaccharide (LPS; 200 ng mL^–1^) in the absence or presence of inhibitors, and the production of nitrite was evaluated through Griess reaction. Cells were pretreated with inhibitors for 1 h before lipopolysaccharide (LPS) stimulation. Values represent the mean and their respective standard deviations from three independent experiments. **: *p* < 0.01; ***: *p* < 0.001 versus LPS-treated cells.

### Drug-like Properties Prediction

The pharmacokinetic properties of the newly synthesized compounds were predicted using the Schrödinger suite. In-silico ADMET/Tox-related parameters were computed with the QikProp application within the Schrödinger software,^46^ which generates a set of physicochemically relevant descriptors then used to evaluate ADMET/Tox properties. The ADMET/Tox-compliance score, a drug-likeness parameter, predicts the pharmacokinetic and toxicological profiles of the ligands, reflects the number of property descriptors calculated by QikProp that fall outside the optimal range observed in 95% of known drugs. As can be appreciated in Table S5 (Supporting Information) all derivatives exhibited favorable QikProp scores, within the favorable range to be considered a drug-type.

In addition, the PAMPA assay^47^ was used to predict the in vitro permeability of compounds **1**, **5–11** and evaluate their brain penetration by passive diffusion.

The results shown that all the compounds would be able to cross the blood–brain barrier (BBB) by passive permeation (Table 3) except the compound **10**, which are in the limit of a positive prediction.

**Table 3:** Predictive Penetration in the CNS in the PAMPA-BBB Assay of Compounds **1**, **5–11**

compd	R1	R2	BBB prediction^a^
1	3-F	Me	CNS+
5	3-OMe	Me	CNS+
6	3,5-diCl	Me	CNS+
7	2-F, 4-Me, 5-OMe	Me	CNS+
8	3-F	H	CNS+
9	3-OMe	H	CNS+
10	3,5-diCl	H	CNS+/–
11	2-F, 4-Me, 5-OMe	H	CNS+

a Data are the mean ± SD of three independent experiments.

### SAS Computational

In order to analyze the experimental results of the developed derivatives, a docking study was conducted. Table 4 shows the interactions and docking scores of the eight synthesized derivatives. The first observation is the similar profile of the NH derivatives and their corresponding methylated counterparts, as they exhibit the same interactions (Figure 9A). This aligns closely with the experimental results, which show no significant differences in activity values between the two groups.

**Table 4:** Docking Scores and Interactions^a^

rank	docking score (kcal/mol)	Glu239/NHPyrrol ring	Leu241/N/CHPyridine ring	Asp247/NPyperidine ring	Asn292/Pyperidine ring	Asp307/Pyperidine ring
1	–13.99	HB	HB/AHB	HB/SBr		
5	–13.826	HB	HB/2xAHB	HB/SBr		
6	–11.724	HB			HB	HB/SBr
7	–13.664	HB	HB/2xAHB	HB/SBr		
8	–13.674	HB	HB/AHB	HB/SBr		
9	–13.082	HB	HB/AHB	HB/SBr		
10	–10.925	HB			HB	SBr
11	–13.516	HB	HB/2xAHB	HB/SBr		

a HB: hydrogen bond; AHB: aromatic hydrogen bond; SBr: salt bridge.

**Figure 9:**
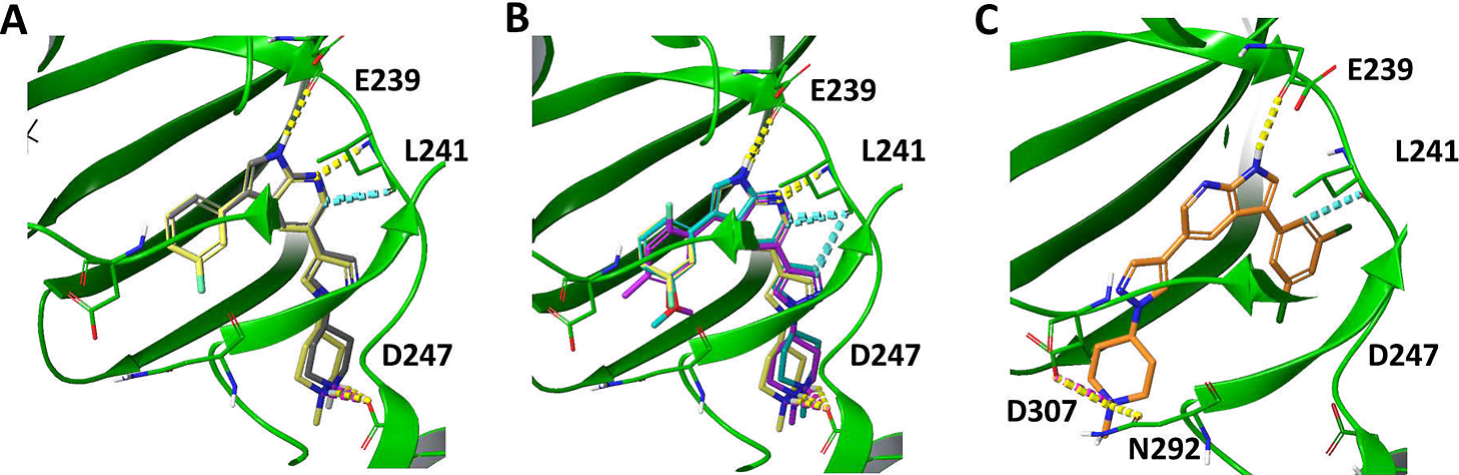
Interactions of hinge residues gk + 1 (E239) and gk + 3 (L241). (A) Comparison between the methylated derivative **1** (yellow) and its corresponding NH counterpart **8** (gray). Interactions of methylated compounds (B) **1** (yellow), **5** (cyan), and **7** (magenta). (C) Interactions of methylated compound **6** (orange).

The second conclusion that can be drawn is the difference in activity observed for derivative **6** (and its NH counterpart) compared to the rest of the compounds. In the other derivatives, the typical key hinge interactions with Glu239 and Leu241 are preserved, which anchor and stabilize the molecules (Figure 9B). However, compound **6** (and **10**) lack these interactions, and their poses are markedly different, significantly impacting their activity (Figure 9C). A possible explanation for this behavior lies in the combination of high electronegativity and large volume of the substituent at R2 (3,5-dichlorophenyl) compared to the other substituents in that position.

To analyze these differences in depth, DFT (density functional theory) and MD (molecular dynamics) calculations were performed to obtain a comprehensive set of molecular descriptors, including the HOMO–LUMO gap, Mulliken charges, NBO interactions, molecular volume and Sterimol parameters (see Supporting Information). The Morfeus software was used at the GFN2-xTB level to enhance descriptor collection, facilitating comprehensive structure–activity relationship analysis.^48,49^ The studied derivatives exhibited significant differences in their interaction with protein residues Asp239 and Leu241. Compounds **1** and **5** displayed nearly identical binding conformations, as their pyrrolo­[2,3-*b*]­pyridine core maintains a comparable electronic environment. This is due to the meta positioning of the fluorine (electron-withdrawing) and methoxy (electron-donating) groups on the phenyl ring, which minimizes their electronic influence on the core. In contrast, compound **7** adopts a distinct binding conformation due to steric repulsion, causing a 70° rotation of the phenyl ring, which alters its orientation at the binding site (see Supporting Information). NBO analysis further supports these findings, showing that the lone pair (LP) electron donation from nitrogen to the conjugated system is stronger in compound **1** (10.34 kcal/mol) than in compound **7** (7.35 kcal/mol), leading to weaker hydrogen bonding interactions in compound **7** (see Table 5).

**Table 5:** Main Donations and Contribution (kcal/mol) of the Electron Density Distribution from the Lone Pairs of Pyrazole and Pyridine Nitrogen in Different Molecules

interaction	compound **1**	compound **6**	compound **7**
LP (Npyrazole) → BD*(π-system)	10.34	9.40	7.35
LP (Npy) → BD*(π-system)	35.13	35.66	34.34
LP (Npy) → BD*(π-system)	49.84	37.74	33.80

Compounds **6** deviates from the trend, exhibiting both steric and electronic effects due to two chlorine atoms at the meta positions (see Supporting Information). Its electron donation energy (9.4 kcal/mol) falls between those of compounds **1** and **7**, affecting its binding interactions. The combined NBO interactions, Sterimol parameters and docking scores provide a comprehensive understanding of how substituent effects on the phenyl ring influence the pyrrolo­[2,3-*b*]­pyridine core’s electronic properties, ultimately dictating protein–ligand binding efficiency.

## Conclusions

The objective of this research was to apply a comprehensive range of AI-based techniques to develop an effective model to generate candidate compounds with good drug-like properties as DYRK1A inhibitors. Conducted under a small-data regime, this study utilized a robust pipeline encompassing *de novo* molecular generation, AI-QSAR modeling, expert knowledge integration, and docking studies.

The strategic application of AI tools, including predictive and generative models, proved highly effective in designing nontoxic DYRK1A inhibitors within a dual-target drug discovery framework. An ensemble model comprising XGBoost, support vector regressors, k-nearest neighbors, and a DMPNN was developed to predict the binding affinity of each compound, while the DMPNN further assessed toxicity profiles. For the generative phase, a hierarchical graph generation model enabled the design of promising DYRK1A inhibitors, facilitating the identification of molecular structures with favorable binding affinity, toxicity, and drug-like properties.

Classical docking studies were employed to prioritize candidates for synthesis and experimental validation. Among these, fluorophenyl-5-methylpiperidinopyrazolyl-1*H*-pyrrolo­[2,3-*b*]­pyridine **1** emerged as the top candidate based on its superior docking score and hinge hydrogen bond interactions. Compound **1** was synthesized and pharmacologically evaluated, demonstrating potent DYRK1A inhibitory activity at the nanomolar level.

Further exploration of this novel compound family resulted in synthesizing and evaluating derivatives (**1**, **5–7**) and the corresponding demethylated (**8–11**), all of which exhibited comparable efficacy. These derivatives also possess additional antioxidant and anti-inflammatory properties, broadening their therapeutic potential.

In conclusion, this study successfully identified a novel DYRK1A inhibitor (**1**) with nanomolar potency using AI-guided methodologies and established a new family of pyrazolylpyrrolo-[2,3-*b*]­pyridine derivatives with promising pharmacological profiles, paving the way for further exploration as potential drugs.

## Experimental Section

### AI-Assisted *De novo* Design

This section provides a and detailed account of the complete process undertaken for the *de novo* generative models that led to the identification of the proposed candidate molecules. It includes an outline of the key characteristics of the data set used, the generative workflow, and additional details necessary to facilitate the reproduction of similar procedures in analogous contexts.

### Data Sets

Two data sets containing molecules in SMILES format were utilized in this work:

- The primary data set consists of 1782 active inhibitors targeting DYRK1A, sourced from the ChEMBL database.^27^ It includes pharmacological activity data for each molecule measured using various methods, such as *K*_i_, *K*_d_, IC_50_ or EC_50_. These values are converted to pChEMBL scores, defined as the negative log_10_ of the molar concentration.
- The Tox21 data set,^50^ comprising 12,060 training samples with 12 binary labels representing the outcomes of 12 distinct toxicological experiments.

### AI-Based QSAR Models

We aim to develop new compounds with high affinity for DYRK1A while maintaining nontoxic profiles, framing this as a dual-objective problem. To achieve this, we first construct QSAR models for each property of interest (affinity and toxicity), enabling us to evaluate the quality of the proposed molecules.

1. Affinity target: As mentioned earlier, to construct the *affinity model*, we employed the primary DYRK1A database. Compared to typical data sets used in QSAR model development, this data set is relatively small, presenting challenges in achieving high-quality predictions. The pChEMBL values were derived different measurements, e.g. *K*_i_, *K*_d_, IC_50_, and EC_50_. As shown in Figure 2, these different measurements require us to conduct standardization and normalization separately for each measurement type. We use a Box–Cox transformation,^51^ and the resulting values were used for performance assessment. This correction was also considered when evaluating each model’s performance.

We employed multiple approaches to develop the predictive affinity model, integrating various molecular representations for each compound to improve accuracy. This process involved selecting the most suitable descriptors for each model, tailored to its specific requirements. The available descriptors were:

- Graph: This representation models a molecule as a graph,^52^ where atoms are nodes and bonds are edges, effectively capturing molecular connectivity and structural relationships.
- Morgan (Morgan fingerprints^53^): Circular fingerprints that encode the local environment around each atom, capturing atom neighborhoods. These are widely used for similarity searches and structure–activity relationship (SAR) analysis.
- Rdkitfpbits:^54^ A representation using bit vectors that denote specific substructures and functional groups within a molecule, enabling rapid identification of molecular features.
- M3C: A frequency-based encoding that quantifies how often each substructure appears within a molecule. This descriptor provides a detailed measure of molecular features and is obtained using the DescriptaStorus package.
- SPGNN-e:^30^ Learned molecular representations derived from graph neural networks (GNNs). These representations capture complex atomic and bonding relationships in a data-driven manner, enhancing predictive accuracy.

Given the available representations of the data set, we employed various predictive algorithms to construct our QSAR model. For each candidate model, multiple combinations of molecular descriptors were tested, selecting the configuration that yielded the most favorable results. Hyper-parameter selection was performed using a grid search with 10-fold cross-validation for each algorithm. The algorithms tested for constructing the QSAR model were:

- MolCLR:^29^ A self-supervised learning framework applied to **Graph** representations. It leverages large unlabeled data sets to pretrain graph neural networks through contrastive learning by maximizing agreement between augmented views of the same molecule, thereby learning meaningful molecular representations.
- SPGNN:^30^ A graph neural network model that operates on **Graph** representations, pretrained with tasks at both the node and graph levels. This approach enhances the model’s ability to learn detailed structural relationships.
- GP (Gaussian Process): Utilizes **M3C** representations to predict molecular properties with uncertainty estimation. This probabilistic model provides confidence intervals for predictions, making it particularly valuable for small data sets.
- RF (Random Forest): Relies on **RDKit** fingerprints to predict molecular properties. It constructs an ensemble of decision trees, averaging predictions across multiple trees to produce robust results.
- MLP (Multilayer Perceptron): Combines **M3C** and **RDKit** fingerprints to enhance predictive power. MLP captures complex relationships by passing the input through multiple layers of interconnected nodes.
- KNN (K-Nearest Neighbors): Uses a combination of **M3C** and **SPGNN-e** representations to predict molecular properties. It determines a molecule’s properties by analyzing its closest neighbors in the data set.
- SVR (Support Vector Regressor): Utilizes both **M3C** and **RDKit** fingerprints for regression-based predictions. SVR identifies a hyperplane in high-dimensional space that best fits the data points for accurate property estimation.
- Chemprop:^17^ A directed message-passing neural network that uses **Graph** representations to predict molecular properties. It computes edge embeddings through message passing and aggregates them into a molecular embedding for prediction.
- XGBoost (Extreme Gradient Boosting):^28^ Employs both **Morgan** and **RDKit** representations to deliver highly accurate and efficient predictions. XGBoost constructs an ensemble of weak learners using gradient boosting, iteratively refining the model to improve performance.

Based on the performance metrics shown in Figure 3, we constructed our primary predictive model as an ensemble consisting of XGBoost,^28^ support vector regressors (SVR), K-nearest neighbors (KNN), and the Chemprop directed message-passing neural network.^17^ SVR contributed strong regression capabilities by employing kernel methods to model nonlinear relationships using M3C and RDKit fingerprints. KNN, combining M3C and SPGNN-e representations, captured local molecular similarities by predicting properties based on the nearest neighbors in the data set. XGBoost, utilizing Morgan and RDKit fingerprints, provided robust and efficient predictions through its gradient boosting algorithm, which iteratively and effectively improves weak learners. Finally, Chemprop enhanced the ensemble with deep learning-based structural insights by leveraging graph representations and directed message-passing mechanisms. This diverse combination allowed the ensemble to produce robust and reliable predictions.

2. Toxicity target: To complete the binary target QSAR model, we developed a model to predict the toxicity of each compound. For this task, we utilized the larger Tox21 data set.^50^ In this data set, each compound is assessed for toxicity across 12 biomarkers, such as the aryl hydrocarbon receptor (AhR) and the estrogen receptor (ER). The selected model is the directed message-passing neural network, implemented within the Chemprop package,^17^ a state-of-the-art algorithm for molecular property prediction. This model operates on molecular graphs, passing messages between atoms and bonds to capture intricate structural relationships and predict chemical properties.

The output of the model is an array of 12 probability values, each representing the likelihood (ranging from 0 to 1) of the compound belonging to a specific toxicity class. This model is employed both as a predictive tool and as a filter in the generative process, requiring candidate molecules to achieve a probability of toxicity below 0.5 across all 12 metrics. This serves as an initial screening step to ensure that generated compounds do not exhibit toxic traits, thereby aligning with the desired drug-like properties. While the cutoff at 0.5 has yielded promising experimental results, stricter thresholds can be applied for individual toxicity labels if a more conservative approach is desired.

In addition to the QSAR models, we implemented a similarity function to enhance the diversity of the proposed molecules. Specifically, the Tanimoto similarity metric was employed to prevent the generation of molecules that were overly similar to one another. For each candidate molecule, its similarity was calculated against all compounds in the existing database, ensuring that the maximum similarity value remained below 0.5. This threshold can also be adjusted to promote an even greater diversity in the exploration of the chemical space, depending on the specific goals of the study.

Together, these QSAR models collectively predict the properties of the proposed compounds and form the foundation for the filters used to screen molecules, which are explicitly detailed in the section on screening filters.

### Generative Models

To generate new candidate molecules, we primarily relied on pretrained generative models, as these typically require large data sets for effective training. This approach enabled us to generate high-quality candidates despite the limited data availability. Given our specific data set constraints, we repurposed pre-existing, pretrained approaches to suit our needs. Among the models considered, the HGG^31^ proved particularly effective due to its ability to process complex molecular data and produce viable molecular structures that met our stringent criteria for affinity, toxicity, and novelty.

The HGG model is a hierarchical graph encoder-decoder model that constructs molecu-les using structural motifs as building blocks. Initially trained on a data set containing SMILES representations of DYRK1A inhibitors, the model generated five batches of 10,000 molecules, iteratively filtering them for binding affinity, toxicity, and structural similarity to known inhibitors. Molecules passing all filtering criteria were reintegrated into the training data set, enabling the model to retrain and progressively refine the candidate list over the five iterations. This iterative approach improved the quality of the final molecule selection, ensuring that each candidate satisfied stringent standards for chemical properties and drug-likeness. While applying such a recursive process blindly could raise concerns about overfitting, in our case, the limited number of iterations ensured the results remained focused on the relevant regions of the chemical space for this specific task. Furthermore, expert chemists reviewed the resulting structures and deemed them both synthetically feasible and chemically interesting. Subsequent experimental validation confirmed these initial evaluations, reinforcing the effectiveness of the HGG model.

For comparison, we briefly explored other models, including Pocket2Mol,^55^ an *E*(3)-equivariant generative network leveraging protein pocket data, as well as several additional algorithms. These included genetic algorithms like the reinforced genetic algorithm (RGA)^56^ and diffusion models such as DiffSBDD.^57^ Although some methods showed potential (e.g., Pocket2Mol) and should be further explored in extensions of this work, others frequently produced molecules with less desirable chemical properties, making them unsuitable for further development.

Finally, the candidates generated by the HGG model underwent conventional molecular docking calculations, yielding scores that surpassed those of the reference ligand. The top-scoring molecules from this process were filtered and ranked using the QSAR ensemble model, with the top 9 compounds displayed in Table 1.

### Screening Filters

To screen the generated molecules, we apply four different types of filters. When needed, we will refer to the predictive QSAR models for pChEMBL affinity and toxicity as *f*_pch_, *f*_tox_, respectively.

- Affinity filter: Given a molecule *G*, its predicted affinity (pChEMBL) must be higher than the third quartile *Q*_3_ of our primary data set:

$$f_{pch}\left(G\right)>Q_{3}$$

This ensures that the molecules generated are somewhat promising candidates for our affinity target.

- Toxicity filter: *G* must be classified as nontoxic in all 12 toxicity classes:

$$\sum_{i=1}^{12}f_{tox}^{\left(i\right)}\left(G\right)=0,,where\;f_{tox}^{\left(i\right)}\left(G\right)=\{\begin{array}{l} 0\;\mathrm{if}\;p\left(tox_{\left(i\right)}|G\right)<0.5\\ 1\;\mathrm{if}\;p\left(tox_{\left(i\right)}|G\right)\geq 0.5 \end{array}$$

where *p*(tox_(*i*)_|*G*) is the estimated probability that the compound *G* is deemed toxic in the *i*-th category *i* ∈ {1, ..., 12}. As mentioned earlier, this 0.5 threshold can be changed if a more conservative estimate of the toxicity of the compounds is needed.

- Similarity filter: We define the similarity between a molecule *G* and our primary data set *D* through

$$s_{D}\left(G\right)=\max_{G^{\prime }\in D}\left\{T\left(G,G^{\prime }\right)\right\}$$

where *T* is the Tanimoto coefficient between two molecules. To ensure that a molecule *G* is sufficiently different from the known inhibitors of the data set, its similarity will have to be less than a predefined value

$$s_{D}\left(G\right)<0.5$$

Increasing this threshold encourages the model to explore more diverse regions of the chemical space but comes with an increased risk of generating nonchemically viable compounds. Conversely, selecting lower values keeps the model closer to the existing data set, prioritizing chemically sound candidates. Empirically, a threshold of 0.5 provided a good balance between these two behaviors. However, depending on the nature of the task and the available data, alternative threshold values may provide a more suitable exploration of the chemical space.

- Internal consistency filter: Given a molecule *G*, the variance of the predicted affinities in the ensemble model, σ_ens_(*G*), must not exceed a preset threshold. σ_thr_ that is

$${\mathrm{\sigma ̂}}_{ens}\left(G\right)<\sigma_{thr}$$

We consider σ_thr_ = 1 for our experiments, although further tests suggest that larger and more permissive thresholds may also work well. This helps ensure certain stability regarding the proposed candidate molecules so no single candidate presents structures that exploit particular parts of the ensemble model. This highlights one of the key strengths of the ensemble model in this context. By incorporating diverse methods within the ensemble, enforcing this condition makes it challenging for the generative process to propose a candidate compound that exploits the specific formulation of any single algorithm. Instead, the compound must perform well across the other components of the ensemble. Therefore, we consider the diversity and collective performance of the ensemble model to be a crucial aspect of our generative process.

### Molecular Docking

Molecular docking was implemented with the following pipeline was applied.

- Ligand Preparation: The conversion from SMILES to SD format was performed using the structconvert tool in the Schrödinger module.^58^ Ligand preparation was conducted using the LigPrep tool included in the Maestro package.^59,60^ Progressive levels were generated, encompassing possible ionization states at physiological pH and potential tautomers. Final energy minimization was carried out using the OPLS4 force field, with default parameters set for stereoisomers.
- Protein Preparation: Human DYRK1A (PDB code 4YLL)^32^ underwent preparation for subsequent computational analyses utilizing Protein Preparation Wizard,^61,62^ a tool integrated into Maestro.^60^ As part of the protocol, the protein structure underwent preprocessing, including bond order assignment and structural adjustments using Prime.^63–65^ Additionally, protonation at pH 7 ± 2 was generated using Epik.^66,67^ Subsequently, optimization of the hydrogen-bonding network and calculation of residue protonation states at pH 7 were performed using PROPKA,^68^ followed by a final restrained minimization employing the OPLS4 force field.
- Ligand Docking: The centroid of the crystallized ligand in the catalytic pocket served as the grid center. During grid generation, a van der Waals radius scaling factor of 1.0 and a partial charge cutoff of 0.25 were applied. Docking was carried out using the Glide extra precision mode (XP) within the Schrodinger software suite,^69–73^ with no constraints applied. Default parameters were utilized for ligand settings, including flexible ligand sampling and the addition of epik state penalties to the docking score. The final step involved postdocking minimization using default settings.
- Docking Validation Protocol: To validate the docking protocol for DYRK1A using the Glide program, we redocked the ligand 4E3 (5t) into the binding site of the crystal structure 4YLL (Table S6 in Supporting Information).

### DFT Calculations

Theoretical calculations were conducted at the DFT level of theory using the Gaussian 16 software. The structures of all intermediates were optimized in gas phase at 298 K and 1 atm. The B3LYP functional, combined with Grimme’s D3 dispersion correction, was used for these optimizations. The basis set was applied, consisting of the 6-31+G­(d,p) basis set for main-group elements. Geometry optimizations were performed without imposing constraints, and the nature of the stationary points was further assessed through vibrational frequency analysis. As expected, all the energy minima were confirmed to display only real vibrational frequencies. Additionally, the software Morfeus (MD) adapted to operate at the GFN2-xTB level of theory, was utilized to enhance descriptor collection, ensuring a robust data set for subsequent analyses (see Supporting Information for references).

### Chemistry

#### Chemistry

Melting points were determined using an MP70 (Mettler Toledo) apparatus and were uncorrected. ^1^H NMR spectra (400 or 500 MHz) and ^13^C NMR spectra (100 or 125 MHz) were recorded on BRUKER AVANCE III HD-400 (400 MHz) and VARIAN SYSTEM-500 (500 MHz) spectrometers and are reported in ppm on the δ scale. The signal of the solvent was used as a reference. High-performance liquid chromatography (HPLC) was performed using a Waters 2695 apparatus with a diode array UV/vis detector Waters 2996 and coupled to a Waters micromass ZQ using a Sunfire C18 column (4.6 × 50 mm, 3.5 μm) at 30 °C, with a flow rate of 0.35 mL/min. The mobile phases used were: CH_3_CN and 0.1% formic acid in H_2_O. Electrospray in positive mode was used for ionization. The sample injection volume was set to 3 μL of a solution of 1 mg/mL CH_3_CN. Gradient conditions, time of gradient (gt) and time of retention (rt) are specified for each case and a different gradient elution was specified for each case. Flash chromatography was performed in an Isolera Prime (Biotage) equipment with a variable detector, using silica gel 60 (230–400 mesh) cartridges or KP C18-HS cartridges, both from Biotage. Elemental analyses were performed on a Heraeus CHN-O Rapid analyzer. Reactions heated by microwaves were realized in a Biotage Initiator microwave oven reactor (frequency of 2045 GHz). All compounds are >95% pure by microanalysis (see Supporting Information). In addition, HPLC chromatograms of compounds **1**, **5–11** have been added in Supporting Information. Reagents and solvents were purchased from common commercial suppliers, mostly Scharlau, BLD and FluoroChem, and were used without further purification. The compound 5-bromo-3-iodo-1-tosyl-1*H*-pyrrolo­[2,3-*b*]­pyridine (**15**) was prepared from the procedure reported in Goodfellow et al.^74^

#### General Procedure for the Synthesis of the 5-Bromo-3-(aryl)-1-tosyl-1*H*-pyrrolo­[2,3-*b*]­pyridine Compounds **2**, **12–14**

A microwave vial was charged with 5-bromo-3-iodo-1-tosyl-1*H*-pyrrolo­[2,3-*b*]­pyridine (**15**), the corresponding aryl-4,4,5,5-tetramethyl-1,3,2-dioxaborolane (**4**, **16–18**), potassium carbonate (K_2_CO_3_) and [1,1′-bis­(diphenylphosphino)­ferrocene] dichloropalladium­(II)­(Pd­(dppf)­Cl_2_). The vial was sealed with a septum cap and purged with argon. 1,4-Dioxane and water were added. The mixture was stirred at rt and bubbled with argon during 5 min. The reaction mixture was irradiated in a microwave for 2 h at 100 °C. The crude reaction mixture was diluted with dichloromethane (CH_2_Cl_2_) and filtered. The solvents were evaporated under a vacuum, and the product was purified by flash chromatography (0–15% EtOAc in hexane).

#### 5-Bromo-3-(3-fluorophenyl)-1-tosyl-1*H*-pyrrolo­[2,3-*b*]­pyridine (**2**)

From 2-(3-fluorophenyl)-4,4,5,5-tetramethyl-1,3,2-dioxaborolane (**4**) (53 mg, 0.25 mmol), 5-bromo-3-iodo-1-tosyl-1*H*-pyrrolo­[2,3-*b*]­pyridine (**15**) (102 mg, 0.21 mmol), K_2_CO_3_ (116 mg, 0.84 mmol, 4% equiv) and Pd­(dppf)­Cl_2_ (8.1 mg, 0.011 mmol, 5% equiv), 1,4-dioxane (3 mL) and H_2_O (0.5 mL). Yield: (51 mg, 55%). mp 163.8–164.4 °C. ^1^H NMR: CDCl_3_ (400 MHz): δ 8.50 (d, 1H, 6-H); 8.20 (d, 1H, 4-H); 8.09 (d, 2H, Ts); 7.90 (s, 1H, 2-H); 7.47–7.42 (m, 1H, Ar); 7.34–7.30 (m, 3H, Ar, Ts); 7.26–7.22 (m, 1H, Ar); 7.11–7.06 (m, 1H, Ar); 2.39 (s, 3H, CH_3_). ^13^C NMR: CDCl_3_ (100 MHz): δ 163.3 (d, *J* = 246 Hz; Ph); 146.1 (C-6); 145.9 (Ts); 145.8 (C-7a); 135.0 (Ts); 134.3 (d, *J* = 8 Hz; Ph); 131.1 (C-4); 131.0 (d, *J* = 8 Hz; Ph); 130.0 (2C, Ts); 128.4 (2C, Ts); 124.5 (C-2); 123.2 (d, *J* = 3 Hz; Ph); 122.9 (C-3a); 118.6 (d, *J* = 2 Hz; C-3); 115.8 (C-5); 115.0 (d, *J* = 21 Hz; Ph); 114.4 (d, *J* = 22 Hz; Ph); 21.8 (CH_3_). HPLC-MS (ES^+^): CH_3_CN/H_2_O 60:40–95:5, gt: 5 min; rt: 5.97; [M + H]^+^, 445/447.

#### 5-Bromo-3-(3-methoxyphenyl)-1-tosyl-1*H*-pyrrolo­[2,3-*b*]­pyridine (**12**)

From 2-(3-methoxyphenyl)-4,4,5,5-tetramethyl-1,3,2-dioxaborolane (**16**) (96 mg, 0.42 mmol), 5-bromo-3-iodo-1-tosyl-1*H*-pyrrolo­[2,3-*b*]­pyridine (**15**) (152 mg, 0.32 mmol), K_2_CO_3_ (227 mg, 1.64 mmol, 4% equiv) and Pd­(dppf)­Cl_2_ (12 mg, 0.016 mmol, 5% equiv), 1,4-dioxane (3 mL) and H_2_O (0.5 mL). Yield: (133 mg, 58%). mp 165.8–166.3 °C. ^1^H NMR: CDCl_3_ (400 MHz): δ 8.48 (d, 1H, 6-H); 8.21 (d, 1H, 4-H); 8.09 (d, 2H, Ts); 7.89 (s, 1H, 2-H); 7.38 (t, 1H, Ar); 7.29 (d, 2H, Ts); 7.13–7.11 (m, 1H, Ar); 7.07–7.06 (m, 1H, Ar); 6.94–6.91 (m, 1H, Ar); 3.87 (s, 3H, OCH_3_); 2.38 (s, 3H, CH_3_). ^13^C NMR: CDCl_3_ (100 MHz): δ 163.2 (d, *J* = 246 Hz; Ph); 154.6 (CO); 146.3 (C-7a); 145.5 (Ts); 143.3 (C-6); 136.6 (Ind); 135.2 (Ts); 134.9 (d, *J* = 8 Hz; Ph); 130.8 (d, *J* = 8 Hz; Ph); 129.8 (2C, Ts); 128.2 (2C, Ts); 125.1 (C-4); 124.9 (C-2); 124.0 (C-5); 123.8 (Ind); 123.2 (d, *J* = 3 Hz; Ph); 121.4 (C-3a); 119.7 (Ind); 119.1 (d, *J* = 3 Hz; C-3); 114.6 (d, *J* = 21 Hz; Ph); 114.4 (d, *J* = 22 Hz; Ph); 80.0 (OC); 59.7 (CH); 32.5 (Pip); 28.5 (5C, 3*CH_3_, Pip); 24.9 (Pip); 21.7 (CH_3_). HPLC-MS (ES^+^): CH_3_CN/H_2_O 60:40–95:5, gt: 5 min; rt = 5.84; [M + H]^+^, 457/459.

#### 5-Bromo-3-(3,5-dichlorophenyl)-1-tosyl-1*H*-pyrrolo­[2,3-*b*]­pyridine (**13**)

From 2-(3,5-dichlorophenyl)-4,4,5,5-tetramethyl-1,3,2-dioxaborolane (**17**) (249 mg, 0.91 mmol), 5-bromo-3-iodo-1-tosyl-1*H*-pyrrolo­[2,3-*b*]­pyridine (**15**) (400 mg, 0.83 mmol), K_2_CO_3_ (573 mg, 4.15 mmol, 5 equiv) and Pd­(dppf)­Cl_2_ (32 mg, 0.04 mmol, 5% equiv), 1,4-dioxane (3 mL) and H_2_O (0.5 mL). Yield: (297 mg, 72%). mp 187.6–188.2 °C. ^1^H NMR: CDCl_3_ (400 MHz): δ 8.52 (d, 1H, 6-H); 8.15 (d, 1H, 4-H); 8.10 (d, 2H, Ts); 7.91 (s, 1H, 2-H); 7.42 (d, 2H, Ar); 7.37 (t, 1H, Ph); 7.31 (d, 2H, Ts); 2.40 (s, 3H, CH_3_). ^13^C NMR: CDCl_3_ (100 MHz): δ 146.4 (C-6); 146.0 (Ts); 145.7 (C-7a); 135.9 (2C, Ph); 135.2 (Ts); 134.8 (Ph); 130.7 (C-4); 130.0 (2C, Ts); 128.4 (2C, Ts); 128.0 (Ph); 125.8 (2C, Ph); 125.1 (C-2); 122.4 (C-3a); 117.1 (C-3); 115.9 (C-5); 21.8 (CH_3_). HPLC-MS (ES^+^): CH_3_CN/H_2_O 80:20–95:5, gt: 5 min; rt = 4.27; [M + H]^+^, 497.

#### 5-Bromo-3-(2-fluoro-5-methoxy-4-methylphenyl)-1-tosyl-1*H*-pyrrolo­[2,3-*b*]­pyridine (**14**)

From 2-(2-fluoro-5-methoxy-4-methylphenyl)-4,4,5,5-tetra-methyl-1,3,2-dioxaborolane (**18**) (149 mg, 0.56 mmol), 5-bromo-3-iodo-1-tosyl-1*H*-pyrrolo­[2,3-*b*]­pyridine (**15**) (200 mg, 0.41 mmol), K_2_CO_3_ (283 mg, 2.05 mmol, 4 equiv) and Pd­(dppf)­Cl_2_ (15 mg, 0.02 mmol, 5% equiv), 1,4-dioxane (3 mL) and H_2_O (0.5 mL). Yield: (113 mg, 55%). mp 169.3–169.6 °C. ^1^H NMR: CDCl_3_ (400 MHz): δ 8.48 (d, 1H, 6-H); 8.10 (d, 2H, Ts); 8.06 (t, 1H, 4-H); 7.90 (s, 1H, 2-H); 7.30 (d, 2H, Ts); 7.00 (d, 1H, Ar); 6.85 (d, 1H, Ar); 3.86 (s, 3H, OCH_3_); 2.39 (s, 3H, CH_3_); 2.26 (s, 3H, CH_3_). ^13^C NMR: CDCl_3_ (100 MHz): δ 154.3 (d, *J* = 2 Hz; Ph); 153.7 (d, *J* = 239 Hz; Ph); 145.8 (C-6); 145.8 (Ts); 145.5 (C-7a); 135.0 (Ts); 131.9 (d, *J* = 5 Hz; C-4); 129.9 (2C, Ts); 128.9 (d, *J* = 8 Hz; Ph); 128.4 (2C, Ts); 125.7 (d, *J* = 3 Hz; C-2); 123.6 (C-3a); 118.4 (d, *J* = 24 Hz; Ph); 116.6 (d, *J* = 16 Hz; Ph); 115.6 (C-5); 114.3 (C-3); 110.9 (d, *J* = 4 Hz; Ph); 56.2 (OCH_3_); 21.8 (CH_3_); 16.3 (CH_3_). HPLC-MS (ES^+^): CH_3_CN/H_2_O 60:40–95:5, gt: 5 min; rt = 7.02; [M + H]^+^, 489/491.

#### General Procedure for the Synthesis of the *tert*-Butyl 4-(4-(3-(aryl)-1-tosyl-1*H*-pyrrolo­[2,3-*b*]­pyridin-5-yl)-1*H*-pyrazol-1-yl)­piperidine-1-carboxylate Compounds **19–22**

Ȧ microwave vial was charged with the corresponding 5-bromo derivative (**2**, **12–14**), *tert*-butyl 4-(4-(4,4,5,5-tetramethyl-1,3,2-dioxaborolan-2-yl)-1*H*-pyrazol-1-yl)­piperidine-1-carboxylate, potassium carbonate (K_2_CO_3_) and [1,1′-Bis­(diphenylphosphino)­ferrocene]­di-chloropalladium­(II) (Pd­(dppf)­Cl_2_). The vial was sealed with a septum cap and purged with argon. 1,4-Dioxane and water were added. The mixture was stirred at rt and bubbled with argon during 5 min. The reaction mixture was irradiated in microwave for 2 h at 100 °C. The crude reaction mixture was diluted with dichloromethane (CH_2_Cl_2_) and filtered. The solvents were evaporated under a vacuum, and the product was purified by flash chromatography (0–50% EtOAc in hexane).

#### *tert*-Butyl 4-(4-(3-(3-fluorophenyl)-1-tosyl-1*H*-pyrrolo­[2,3-*b*]­pyridin-5-yl)-1*H*-pyrazol-1-yl)­piperidine-1-carboxylate **(19**)

From 5-bromo-3-(3-fluoro-phenyl)-1-tosyl-1*H*-pyrrolo­[2,3-*b*]­pyridine (**2**) (90 mg, 0.2 mmol), *tert*-butyl 4-(4-(4,4,5,5-tetramethyl-1,3,2-dioxaborolan-2-yl)-1*H*-pyrazol-1-yl)­piperidine-1-carboxylate (**23**) (87 mg, 0.23 mmol), K_2_CO_3_ (126 mg, 0.91 mmol, 4.6 equiv) and Pd­(dppf)­Cl_2_ (8.0 mg, 0.011 mmol, 5% equiv), dioxane (3 mL) and H_2_O (0.5 mL). Yield: (116 mg, 94%). mp 106.8–107.2 °C. ^1^H NMR: CDCl_3_ (400 MHz): δ 8.61 (d, 1H, 6-H); 8.13 (d, 2H, Ts); 8.08 (d, 1H, 4-H); 7.89 (s, 1H, 2-H); 7.79 (d, 1H, Ind); 7.73 (d, 1H, Ind); 7.49–7.43 (m, 1H, Ar); 7.40–7.37 (m, 1H, Ar); 7.32–7.29 (m, 3H, Ar, Ts); 7.11–7.08 (m, 1H, Ar); 4.37–4.28 (m, 3H, Pip); 2.96–2.89 (m, 2H, Pip); 2.38 (s, 3H, CH_3_); 2.19 (d, 2H, Pip); 2.03–1.93 (m, 2H, Pip); 1.49 (s, 9H, CH_3_). ^13^C NMR: CDCl_3_ (100 MHz): δ 163.2 (d, *J* = 246 Hz; Ph); 154.6 (CO); 146.3 (C-7a); 145.5 (Ts); 143.3 (C-6); 136.7 (Ind); 135.4 (Ts); 134.9 (d, *J* = 8 Hz; Ph); 130.8 (d, *J* = 8 Hz; Ph); 129.8 (2C, Ts); 128.2 (2C, Ts); 125.1 (C-4); 124.9 (C-5); 124.0 (Ind); 123.2 (C-2); 123.2 (d, *J* = 3 Hz; Ph); 121.4 (C-3a); 119.7 (Ind); 119.1 (d, *J* = 2 Hz; C-3); 114.6 (d, *J* = 21 Hz; Ph); 114.4 (d, *J* = 22 Hz; Ph); 80.0 (OC); 59.7 (CH); 32.5 (Pip); 24.5 (5C, 3*CH_3_, Pip); 24.9 (Pip); 21.7 (CH_3_). HPLC-MS (ES^+^): CH_3_CN/H_2_O 60:40–95:5, gt: 5 min; rt: 5.77; [M + H]^+^, 616.

#### *tert*-Butyl 4-(4-(3-(3-methoxyphenyl)-1-tosyl-1*H*-pyrrolo­[2,3-*b*]­pyridin-5-yl)-1*H*-pyrazol-1-yl)­piperidine-1-carboxylate (**20**)

From 5-bromo-3-(3-methoxyphenyl)-1-tosyl-1*H*-pyrrolo­[2,3-*b*]­pyridine (**12**) (150 mg, 0.33 mmol), *tert*-butyl 4-(4-(4,4,5,5-tetramethyl-1,3,2-dioxaborolan-2-yl)-1*H*-pyrazol-1-yl)­piperidine-1-carboxylate (**23**) (137 mg, 0.36 mmol), K_2_CO_3_ (137 mg, 0.99 mmol, 3 equiv) and Pd­(dppf)­Cl_2_ (12.1 mg, 0.017 mmol, 5% equiv), dioxane (3 mL) and H_2_O (0.5 mL). Yield: (176 mg, 85%). mp 88.5–89.2 °C. ^1^H NMR: CDCl_3_ (400 MHz): δ 8.59 (d, 1H, 6-H); 8.11 (d, 2H, Ts); 8.08 (d, 1H, 4-H); 7.86 (s, 1H, 2-H); 7.77 (d, 1H, Ind); 7.67 (d, 1H, Ind); 7.41 (t, 1H, Ar); 7.29 (d, 2H, Ts); 7.18 (m, 1H, Ar); 7.11 (m, 1H, Ar); 6.93 (m, 1H, Ar); 4.34–4.26 (m, 3H, Pip); 3.88 (s, 3H, OCH_3_); 2.90 (t, 2H, Pip); 2.37 (s, 3H, CH_3_); 2.18–2.14 (m, 2H, Pip); 2.01–1.90 (m, 2H, Pip); 1.48 (s, 9H, CH_3_). ^13^C NMR: CDCl_3_ (100 MHz) δ:160.3 (Ph); 154.7 (CO); 146.5 (C-7a); 145.4 (Ts); 143.2 (C-6); 136.7 (Ind); 135.5 (Ts); 134.1 (Ph); 130.3 (Ph); 129.8 (2C, Ts); 128.2 (2C, Ts); 125.5 (C-4); 124.8 (C-2); 124.0 (C-5); 123.5 (Ind); 121.8 (C-3a); 120.3 (Ph); 120.1 (Ind); 119.9 (C-3); 113.5 (Ph); 113.1 (Ph); 80.1 (OC); 59.8 (CH); 55.6 (OCH_3_); 32.6 (2C, Pip); 28.6 (5C, 3*CH_3_, Pip); 21.8 (CH_3_). HPLC-MS (ES^+^): CH_3_CN/H_2_O 60:40–95:5, gt: 5 min; rt = 5.61; [M + H]^+^, 628.

#### *tert*-Butyl 4-(4-(3-(3,5-dichlorophenyl)-1-tosyl-1*H*-pyrrolo­[2,3-*b*]­pyridin-5-yl)-1*H*-pyrazol-1-yl)­piperidine-1-carboxylate (**21**)

From 5-bromo-3-(3,5-dichloro phenyl)-1-tosyl-1*H*-pyrrolo­[2,3-*b*]­pyridine (**13**) (182 mg, 0.37 mmol), *tert*-butyl 4-(4-(4,4,5,5-tetramethyl-1,3,2-dioxaborolan-2-yl)-1*H*-pyrazol-1-yl)-piperidine-1-carboxylate (**23**) (153 mg, 0.41 mmol), K_2_CO_3_ (153 mg, 1.11 mmol, 3 equiv) and Pd­(dppf)­Cl_2_ (14 mg, 0.019 mmol, 5% equiv), dioxane (3 mL) and H_2_O (0.5 mL). Yield: (173 mg, 71%). mp 117.2–117.6 °C. ^1^H NMR: CDCl_3_ (400 MHz): δ 8.54 (d, 1H, 6-H); 8.10 (d, 2H, Ts); 8.01 (d, 1H, 4-H); 7.86 (s, 1H, 2-H); 7.79 (s, 1H, Ind); 7.76 (s, 1H, Ind); 7.44 (d, 2H, Ar); 7.27–7.25 (m, 3H, Ts, Ar); 4.34–4.22 (m, 3H, Pip); 2.89 (br s, 2H, Pip); 2.33 (s, 3H, CH_3_); 2.13 (d, 2H, Pip); 2.04–1.90 (m, 2H, Pip); 1.46 (s, 9H, CH_3_). ^13^C NMR: CDCl_3_ (100 MHz): δ 154.2 (CO); 145.7 (C-7a); 145.3 (Ts); 143.2 (C-6); 136.3 (Ind); 135.6 (Ts); 135.3 (2C, Ph); 134.8 (Ph); 129.5 (2C, Ts); 128.0 (2C, Ts); 127.2 (Ph); 125.5 (2C, Ph); 125.5 (C-4); 124.9 (C-2); 124.5 (Ph); 124.2 (C-5); 124.1 (Ind); 121.8 (C-3a); 119.1 (Ind); 117.3 (C-3); 79.5 (OC); 59.3 (CH); 32.1 (2C, Pip); 28.2 (3C, 3*CH_3_); 24.6 (2C, Pip); 21.4 (CH_3_). HPLC-MS (ES^+^): CH_3_CN/H_2_O 80:20–95:5, gt: 5 min; rt = 3.95; [M + H]^+^, 666.

#### *tert*-Butyl 4-(4-(3-(2-fluoro-5-methoxy-4-methylphenyl)-1-tosyl-1*H*-pyrrolo [2,3-*b*]­pyridin-5-yl)-1*H*-pyrazol-1-yl)­piperidine-1-carboxylate (**22**)

From 5-bromo-3-(2-fluoro-5-methoxy-4-methylphenyl)-1-tosyl-1*H*-pyrrolo­[2,3-*b*]­pyridine (**14**) (200 mg, 0.41 mmol), *tert*-butyl 4-(4-(4,4,5,5-tetramethyl-1,3,2-dioxaborolan-2-yl)-1*H*-pyrazol-1-yl)­piperi-dine-1-carboxylate (**23**) (170 mg, 0.45 mmol), K_2_CO_3_ (170 mg, 1.23 mmol, 3 equiv) and Pd­(dppf)­Cl_2_ (14.6 mg, 0.017 mmol, 5% equiv), dioxane (3 mL) and H_2_O (0.5 mL). Yield: (195 mg, 73%). mp 163.1–163.5 °C. ^1^H NMR: CDCl_3_ (400 MHz): δ 8.58 (d, 1H, 6-H); 8.12 (d, 2H, Ts); 7.94 (d, 1H, 4-H); 7.87 (s, 1H, 2-H); 7.75 (s, 1H, Ind); 7.66 (s, 1H, Ind); 7.29 (d, 2H, Ts); 7.00 (d, 1H, Ar); 6.89 (d, 1H, Ar); 4.33–4.24 (m, 3H, Pip); 3.86 (s, 3H, OCH_3_); 2.94–2.86 (m, 2H, Pip); 2.37 (s, 3H, CH_3_); 2.26 (s, 3H, CH_3_); 2.17–2.13 (m, 2H, Pip); 2.00–1.89 (m, 2H, Pip); 1.47 (s, 9H, CH_3_). ^13^C NMR: CDCl_3_ (100 MHz): δ 154.7 (CO); 154.2 (d, *J* = 2 Hz; Ph); 153.8 (d, *J* = 239 Hz; Ph); 146.0 (C-7a); 145.5 (Ts); 143.2 (C-6); 136.7 (Ind); 135.4 (Ts); 129.8 (2C, Ts); 128.6 (d, *J* = 8 Hz; Ph); 128.3 (2C, Ts); 126.1 (d, *J* = 3 Hz; C-4); 124.9 (d, *J* = 3 Hz; C-2); 124.7 (C-5); 123.9 (Ind); 122.2 (C-3a); 120.0 (Ind); 118.3 (d, *J* = 24 Hz, Ph); 117.2 (d, *J* = 16 Hz; Ph); 114.9 (C-3); 111.1 (d, *J* = 4 Hz; Ph); 80.1 (OC); 59.7 (CH); 56.2 (OCH_3_); 32.5 (2C, Pip); 28.5 (5C, 3*CH_3_, Pip); 21.8 (CH_3_); 16.2 (d, *J* = 1 Hz, CH_3_). HPLC-MS (ES^+^): CH_3_CN/H_2_O 60:40–95:5, gt: 5 min; rt = 6.58; [M + H]^+^, 660.

#### General Procedure for the Synthesis of the 3-(Aryl)-5-(1-(piperidin-4-yl)-1*H*-pyrazol-4-yl)-1-tosyl-1*H*-pyrrolo­[2,3-*b*]­pyridine Compounds **24–27**

Trifluoroacetic acid (TFA) was added to a solution of the corresponding Boc-protected compound (**19–22**) in dichloromethane at rt. The reaction is stirred until the end of the reaction. The solvent was evaporated under a vacuum. The residue was suspended in NaHCO_3_ aq 1 M (10 mL) and the resulting suspension was cooled to 4 °C (overnight). The final product was obtained by filtration, washed with water and air-dried.

#### 3-(3-Fluorophenyl)-5-(1-(piperidin-4-yl)-1*H*-pyrazol-4-yl)-1-tosyl-1*H*-pyrrolo [2,3-*b*]­pyridine (**24**)

From *tert*-butyl 4-(4-(3-(3-fluorophenyl)-1-tosyl-1*H*-pyrrolo­[2,3-*b*]­pyridin-5-yl)-1*H*-pyrazol-1-yl)­piperidine-1-carboxylate (**19**) (106 mg, 0.17 mmol) in CH_2_Cl_2_ (10 mL) and TFA (2 mL). Yield: (68 mg, 78%). mp 141.8–142.6 °C. ^1^H NMR: CDCl_3_ (400 MHz): δ 8.61 (d, 1H, 6-H); 8.12 (d, 2H, Tos); 8.06 (d, 1H, 4-H); 7.88 (s, 1H, 2-H); 7.77 (s, 1H, Ind); 7.70 (s, 1H, Ind); 7.47–7.43 (m, 1H, Ar); 7.39–7.37 (m, 1H, Ar); 7.31–7.28 (m, 3H, Ar, Tos); 7.11–7.06 (m, 1H, Ar); 4.29–4.24 (m, 1H, CH); 3.29–3.24 (m, 2H, Pip); 2.79 (t, 2H, Pip); 2.38 (s, 3H, CH_3_); 2.22–2.18 (m, 2H, Pip); 1.98–1.88 (m, 2H, Pip). ^13^C NMR: Acetone-*d*_6_ (100 MHz): δ 164.0 (d, *J* = 243 Hz; Ph); 146.9 (C-7a); 145.6 (Ts); 143.6 (C-6); 136.9 (Ind); 136.2 (Ts); 136.0 (d, *J* = 8 Hz; Ph); 131.7 (d, *J* = 9 Hz; Ph); 130.6 (2C, Ts); 129.0 (2C, Ts); 126.5 (C-5); 125.7 (C-4); 125.6 (C-2); 125.0 (Ind); 124.3 (d, *J* = 2 Hz; Ph); 121.9 (C-3a); 119.8 (Ind); 119.7 (d, *J* = 2 Hz; C-3); 115.0 (d, *J* = 21 Hz; Ph); 114.6 (d, *J* = 21 Hz; Ph); 60.2 (CH); 45.6 (2C, Pip); 33.9 (2C, Pip); 21.5 (CH_3_). HPLC-MS (ES^+^): CH_3_CN/H_2_O 15:85–95:5, gt: 5 min; rt: 5.09; [M + H]^+^, 516.

#### 3-(3-Methoxyphenyl)-5-(1-(piperidin-4-yl)-1*H*-pyrazol-4-yl)-1-tosyl-1*H*-pyrrolo [2,3-*b*]­pyridine (**25**)

From *tert*-butyl 4-(4-(3-(3-methoxyphenyl)-1-tosyl-1*H*-pyrrolo­[2,3-*b*]­pyridin-5-yl)-1*H*-pyrazol-1-yl)­piperidine-1-carboxylate (**20**) (230 mg, 0.37 mmol) in CH_2_Cl_2_ (10 mL) and TFA (2 mL). Yield: (192 mg, 98%). mp 121.8–122.3 °C. ^1^H NMR: CDCl_3_ (400 MHz): δ 8.59 (d, 1H, 6-H); 8.12–8.09 (m, 3H, Ts, 4-H); 7.86 (s, 1H, 2-H); 7.76 (d, 1H, Ind); 7.69 (d, 1H, Ind); 7.41 (t, 1H, Ar); 7.28 (d, 2H, Ts); 7.18 (d, 1H, Ar); 7.12–7.11 (m, 1H, Ar); 6.95–6.92 (m, 1H, Ar); 4.28–4.22 (m, 1H, Pip); 3.88 (s, 3H, OCH_3_); 3.28–3.23 (m, 2H, Pip); 2.81–2.74 (m, 2H, Pip); 2.37 (s, 3H, CH_3_); 2.21–2.16 (d, 2H, Pip); 1.98–1.87 (m, 2H, Pip). ^13^C NMR: CDCl_3_ (100 MHz): δ 160.3 (Ph); 146.4 (C-7a); 145.4 (Ts); 143.3 (C-6); 136.5 (Ind); 135.5 (Ts); 134.1 (Ph); 130.3 (Ph); 129.8 (2C, Ts); 128.2 (2C, Ts); 125.5 (C-4); 125.0 (C-2); 123.8 (C-5); 123.5 (Ind); 121.8 (C-3a); 120.3 (Ind); 120.1 (C-3); 119.8 (C-3); 113.5 (Ph); 113.1 (Ph); 60.1 (CH); 55.5 (OCH_3_); 45.8 (2C, Pip); 34.1 (2C, Pip); 21.8 (CH_3_). HPLC-MS (ES^+^): CH_3_CN/H_2_O 15:85–95:5, gt: 5 min; rt = 4.97; [M + H]^+^, 528.

#### 3-(3,5-Dichlorophenyl)-5-(1-(piperidin-4-yl)-1*H*-pyrazol-4-yl)-1-tosyl-1*H*-py-rrolo­[2,3-*b*]­pyridine (**26**)

From *tert*-butyl 4-(4-(3-(3,5-dichlorophenyl)-1-tosyl-1*H*-pyrro-lo­[2,3-*b*]­pyridin-5-yl)-1*H*-pyrazol-1-yl)­piperidine-1-carboxylate (**21**) (306 mg, 0.46 mmol) in CH_2_Cl_2_ (10 mL) and TFA (2 mL). Yield: (213 mg, 82%). mp 132.9–133.5 °C. ^1^H NMR: CDCl_3_ (400 MHz): δ 8.62 (d, 1H, 6-H); 8.12 (d, 2H, Ts); 8.01 (d, 1H, 4-H); 7.88 (s, 1H, 2-H); 7.78 (s, 1H, Ind); 7.71 (s, 1H, Ind); 7.46 (s, 2H, Ar); 7.37 (s, 1H, Ar); 7.30 (d, 2H, Ts); 4.31–4.23 (m, 1H, Pip); 3.28–3.23 (m, 2H, Pip); 2.78 (t, 2H, Pip); 2.38 (s, 3H, CH_3_); 2.21–2.17 (m, 2H, Pip); 1.99–1.90 (m, 2H, Pip). ^13^C NMR: CDCl_3_ (100 MHz): δ 146.2 (C-7a); 145.7 (Ts); 143.8 (C-6); 136.5 (Ind); 135.9 (Ts); 135.8 (2C, Ph); 135.2 (Ph); 129.9 (2C, Ts); 128.4 (2C, Ts); 127.8 (Ph); 125.9 (2C, Ph); 125.3 (C-4); 124.9 (C-2); 124.3 (C-5); 123.9 (Ind); 121.0 (C-3a); 119.5 (Ind); 117.8 (C-3); 60.2 (CH); 45.8 (2C, Pip); 34.1 (2C, Pip); 21.8 (CH_3_). HPLC-MS (ES^+^): CH_3_CN/H_2_O 15:85–95:5, gt: 5 min; rt = 5.41; [M + H]^+^, 566/568.

#### 3-(2-Fluoro-5-methoxy-4-methylphenyl)-5-(1-(piperidin-4-yl)-1*H*-pyrazol-4-yl)-1-tosyl-1*H*-pyrro-lo­[2,3-*b*]­pyridine (**27**)

From *tert*-butyl 4-(4-(3-(2-fluoro-5-methoxy-4-methylphenyl)-1-tosyl-1*H*-pyrrolo­[2,3-*b*]­pyridin-5-yl)-1*H*-pyrazol-1-yl)­piperidine-1-carboxy-late (**22**) (246 mg, 0.37 mmol) in CH_2_Cl_2_ (10 mL) and TFA (2 mL). Yield: (203 mg, 98%). mp 117.9–118.6 °C. ^1^H NMR: CDCl_3_ (400 MHz): δ 8.59 (d, 1H, 6-H); 8.12 (d, 2H, Ts); 7.95 (t, 1H, 4-H); 7.88 (s, 1H, 2-H); 7.74 (s, 1H, Ind); 7.68 (s, 1H, Ind); 7.29 (d, 2H, Ts); 7.01 (d, 1H, Ar); 6.90 (d, 1H, Ar); 4.29–4.21 (m, 1H, CH); 3.86 (s, 3H, OCH_3_); 3.28–3.24 (m, 2H, Pip); 2.78 (td, 2H, Pip); 2.37 (s, 3H, CH_3_); 2.27 (s, 3H, CH_3_); 2.20–2.16 (m, 2H, Pip); 1.95–1.91 (m, 2H, Pip). ^13^C NMR: CDCl_3_ (100 MHz): δ 154.2 (d, *J* = 2 Hz; Ph); 153.8 (d, *J* = 239 Hz; Ph); 146.0 (C-7a); 145.4 (Ts); 143.2 (C-6); 136.4 (Ind); 135.4 (Ts); 129.8 (2C, Ts); 128.6 (d, *J* = 8 Hz; Ph); 128.3 (2C, Ts); 126.1 (d, *J* = 4 Hz; C-4); 124.9 (d, *J* = 3 Hz; C-2); 124.8 (C-5); 123.7 (Ind); 122.2 (C-3a); 119.8 (Ind); 118.4 (d, *J* = 23 Hz, Ph); 117.2 (d, *J* = 16 Hz; Ph); 115.0 (C-3); 111.2 (d, *J* = 5 Hz; Ph); 60.0 (CH); 56.2 (OCH_3_); 45.8 (2C, Pip); 34.0 (2C, Pip); 21.8 (CH_3_); 16.3 (CH_3_). HPLC-MS (ES^+^): CH_3_CN/H_2_O 15:85–95:5, gt: 5 min; rt: 5.26; [M + H]^+^, 560.

#### General Procedure for the Synthesis of the 3-(Aryl)-5-(1-(1-methyl-piperidin-4-yl)-1*H*-pyrazol-4-yl)-1-tosyl-1*H*-pyrrolo­[2,3-*b*]­pyridine Compounds **28–31**

Over a solution of the corresponding piperidine derivative (**24–27**) in formic acid at rt, was added, dropwise, formaldehyde 37% aqueous. The mixture was stirred and heated to 70 °C until the end of the reaction. The solvent was evaporated under a vacuum. A saturated aqueous solution of sodium carbonate (Na_2_CO_3_) was added (to pH = 10) and the obtained suspension was cooled to 4 °C (overnight). The final product was obtained by filtration, washed with water and air-dried.

#### 3-(3-Fluorophenyl)-5-(1-(1-methylpiperidin-4-yl)-1*H*-pyrazol-4-yl)-1-tosyl-1*H*-pyrrolo­[2,3-*b*]­pyridine (**28**)

From 3-(3-fluorophenyl)-5-(1-(piperidin-4-yl)-1*H*-pyrazol-4-yl)-1-tosyl-1*H*-pyrrolo­[2,3-*b*]­pyridine (24) (120 mg, 0.23 mmol), formic acid (2 mL) and formaldehyde 37% aqueous (0.7 mL, 4.4 mmol). Time of reaction: 24 h. Yield: (111 mg, 89%). mp 191.7–192.3 °C. ^1^H NMR: CDCl_3_ (400 MHz): δ 8.62 (d, 1H, 6-H); 8.13 (d, 2H, Tos); 8.08 (d, 1H, 4-H); 7.89 (s, 1H, 2-H); 7.79 (s, 1H, Ind); 7.73 (s, 1H, Ind); 7.49–7.43 (m, 1H, Ar); 7.40–7.36 (m, 1H, Ar); 7.32–7.29 (m, 3H, Ar, Tos); 7.11–7.06 (m, 1H, Ar); 4.20–4.14 (m, 1H, CH); 3.00 (d, 2H, Pip); 2.38 (s, CH_3_); 2.34 (s, NCH_3_); 2.22–2.08 (m, 6H, Pip). ^13^C NMR: CDCl_3_ (100 MHz): δ 163.2 (d, *J* = 245 Hz; Ph); 146.2 (C-7a); 145.4 (Ts); 143.3 (C-6); 136.3 (Ind); 135.2 (Ts); 134.9 (d, *J* = 8 Hz; Ph); 130.8 (d, *J* = 9 Hz; Ph); 129.8 (2C, Ts); 128.2 (2C, Ts); 125.1 (C-4); 125.0 (C-2); 123.7 (C-5); 123.6 (Ind); 123.2 (d, *J* = 3 Hz; Ph); 121.4 (C-3a); 119.6 (Ind); 119.1 (d, *J* = 3 Hz; C-3); 114.6 (d, *J* = 21 Hz; Ph); 114.3 (d, *J* = 22 Hz; Ph); 59.3 (CH); 54.7 (2C, Pip); 46.0 (NCH_3_); 32.6 (2C, Pip); 21.7 (CH_3_). HPLC-MS (ES^+^): CH_3_CN/H_2_O 15:85–95:5, gt: 5 min; rt = 5.09; [M + H]^+^, 530.

#### 3-(3-Methoxyphenyl)-5-(1-(1-methylpiperidin-4-yl)-1*H*-pyrazol-4-yl)-1-tosyl-1*H*-pyrrolo­[2,3-*b*]­pyridine (**29**)

From 3-(3-methoxyphenyl)-5-(1-(piperidin-4-yl)-1*H*-pyrazol-4-yl)-1-tosyl-1*H*-pyrrolo­[2,3-*b*]­pyridine (**25**) (120 mg, 0.23 mmol), formic acid (2 mL), and formaldehyde 37% aqueous (0.7 mL, 4.4 mmol). Time of reaction: 18 h. Yield: (111 mg, 89%). mp 147.9–148.6 °C. ^1^H NMR: CDCl_3_ (400 MHz): δ 8.60 (d, 1H, 6-H); 8.13–8.08 (m, 3H, Ts, 4-H); 7.87 (s, 1H, 2-H); 7.77 (s, 1H, Ind); 7.71 (s, 1H, Ind); 7.41 (t, 1H, Ar); 7.29 (d, 2H, Ts); 7.19 (d, 1H, Ar); 7.13 (s, 1H, Ar); 6.94 (d, 1H, Ar); 4.19–4.13 (m, 1H, Pip); 3.88 (s, 3H, OCH_3_); 3.01–2.98 (m, 2H, Pip); 2.37 (s, 3H, CH_3_); 2.34 (s, NCH_3_); 2.22–2.07 (m, 6H, Pip). ^13^C NMR: CDCl_3_ (100 MHz): δ 160.2 (Ph); 146.4 (C-7a); 145.3 (Ts); 143.2 (C-6); 136.4 (Ind); 135.4 (Ts); 134.0 (Ph); 130.2 (Ph); 129.8 (2C, Ts); 128.2 (2C, Ts); 125.4 (C-4); 124.9 (C-2); 123.7 (C-5); 123.4 (Ind); 121.8 (C-3a); 120.3 (Ph); 120.0 (Ind); 119.8 (C-3); 113.4 (Ph); 113.1 (Ph); 59.3 (CH); 55.4 (OCH_3_); 54.7 (2C, Pip); 46.1 (NCH_3_); 32.7 (2C, Pip); 21.7 (CH_3_). HPLC-MS (ES^+^): CH_3_CN/H_2_O 15:85–95:5, gt: 5 min; rt = 5.11; [M + H]^+^, 542.

#### 3-(3,5-Dichlorophenyl)-5-(1-(1-methylpiperidin-4-yl)-1*H*-pyrazol-4-yl)-1-tosyl-1*H*-pyrrolo­[2,3-*b*]­pyridine (**30**)

From 3-(3,5-dichlorophenyl)-5-(1-(piperidin-4-yl)-1*H*-pyrazol-4-yl)-1-tosyl-1*H*-pyrrolo­[2,3-*b*]­pyridine (**26**) (120 mg, 0.21 mmol), formic acid (5 mL), and formaldehyde 37% aqueous (2 mL, 4.4 mmol). Time of reaction: 28 h. Yield: (103 mg, 84%). mp 113.8–114.5 °C. ^1^H NMR: CDCl_3_ (400 MHz): δ 8.61 (d, 1H, 6-H); 8.12 (d, 2H, Ts); 8.01 (d, 1H, 4-H); 7.88 (s, 1H, 2-H); 7.77 (s, 1H, Ind); 7.70 (s, 1H, Ind); 7.46 (d, 2H, Ar); 7.37 (t, 1H, Ar); 7.30 (d, 2H, Ts); 4.22–4.13 (m, 1H, Pip); 3.01–2.98 (m, 2H, Pip); 2.38 (s, 3H, CH_3_); 2.34 (s, 3H, NCH_3_); 2.22–2.10 (m, 6H, Pip). ^13^C NMR: CDCl_3_ (100 MHz): δ 146.2 (C-7a); 145.7 (Ts); 143.8 (C-6); 136.5 (Ind); 135.9 (Ts); 135.8 (2C, Ph); 135.2 (Ph); 129.9 (2C, Ts); 128.4 (2C, Ts); 127.8 (Ph); 125.9 (2C, Ph); 125.4 (C-4); 124.9 (C-2); 124.3 (C-5); 124.0 (Ind); 121.0 (C-3a); 119.5 (Ind) 117.8 (C-3); 59.5 (CH); 54.8 (2C, Pip); 46.2 (NCH_3_); 32.8 (2C, Pip); 21.8 (CH_3_). HPLC-MS (ES^+^): CH_3_CN/H_2_O 15:85–95:5, gt: 5 min; rt = 5.47; [M + H]^+^, 580/582.

#### 3-(2-Fluoro-5-methoxy-4-methylphenyl)-5-(1-(1-methylpiperidin-4-yl)-1*H*-py-razol-4-yl)-1-tosyl-1*H*-pyrrolo­[2,3-*b*]­pyridine **(31**)

From 3-(2-fluoro-5-methoxy-4-methylphenyl)-5-(1-(piperidin-4-yl)-1*H*-pyrazol-4-yl)-1-tosyl-1*H*-pyrrolo­[2,3-*b*]­pyridine (**27**) (103 mg, 0.18 mmol), formic acid (5 mL) and formaldehyde 37% aqueous (2 mL, 4.4 mmol). Time of reaction: 18 h. Yield: (90 mg, 87%). mp 113.9–114.6 °C. ^1^H NMR: CDCl_3_ (400 MHz): δ 8.58 (d, 1H, 6-H); 8.12 (d, 2H, Ts); 7.94 (t, 1H, 4-H); 7.88 (s, 1H, 2-H); 7.73 (s, 1H, Ind); 7.66 (s, 1H, Ind); 7.29 (d, 2H, Ts); 7.01 (d, 1H, Ar); 6.90 (d, 1H, Ar); 4.17–4.12 (m, 1H, CH); 3.86 (s, 3H, OCH_3_); 2.99–2.96 (m, 2H, Pip); 2.37 (s, 3H, CH_3_); 2.33 (s, NCH_3_); 2.27 (s, 3H, CH_3_); 2.19–2.07 (m, 6H, Pip). ^13^C NMR: CDCl_3_ (100 MHz): δ 154.2 (d, *J* = 1 Hz; Ph); 153.8 (d, *J* = 239 Hz; Ph); 146.0 (C-7a); 145.4 (Ts); 143.2 (C-6); 136.4 (Ind); 135.4 (Ts); 129.9 (2C, Ts); 128.6 (d, *J* = 8 Hz; Ph); 128.3 (2C, Ts); 126.1 (d, *J* = 5 Hz; C-4); 124.9 (d, *J* = 3 Hz; C-2); 124.8 (C-5); 123.6 (Ind); 122.2 (C-3a); 119.9 (Ind); 118.4 (d, *J* = 24 Hz, Ph); 117.3 (d, *J* = 16 Hz; Ph); 115.0 (C-3); 111.2 (d, *J* = 5 Hz; Ph); 59.4 (CH); 56.2 (OCH_3_); 54.8 (2C, Pip); 46.2 (NCH_3_); 32.8 (2C, Pip); 21.8 (CH_3_); 16.3 (CH_3_). HPLC-MS (ES^+^): CH_3_CN/H_2_O 15:85–95:5, gt: 5 min; rt = 5.28; [M + H]^+^, 574.

#### General Procedure for the Synthesis of the 3-(Aryl)-5-(1-(1-methylpipe-ridin-4-yl)-1*H*-pyrazol-4-yl)-1*H*-pyrrolo­[2,3-*b*]­pyridine Compounds **1**, **5–11**

A solution of the corresponding tosyl derivative (**24–31**) in 0.4 M NaOH methanolic solution was stirred at room temperature until the end of the reaction. The solvent was evaporated under a vacuum. Water was added and the obtained suspension was cooled to 4 °C (overnight). The final product was obtained by filtration, washed with water and air-dried.

#### 3-(3-Fluorophenyl)-5-(1-(1-methylpiperidin-4-yl)-1*H*-pyrazol-4-yl)-1*H*-pyrro-lo­[2,3-*b*]­pyridine (**1**)

From 3-(3-fluorophenyl)-5-(1-(1-methylpiperidin-4-yl)-1*H*-pyrazol-4-yl)-1-tosyl-1*H*-pyrrolo­[2,3-*b*]­pyridine (**28**) (55 mg, 0.1 mmol) and 0.4 M NaOH methanolic solution (20 mL). Time of reaction: 2 h. Yield: (32 mg, 90%). mp 234.6–235.8 °C. ^1^H NMR: DMSO-*d*_6_ (400 MHz): δ 11.99 (br s, 1H, NH); 8.56 (d, 1H, 6-H); 8.41 (d, 1H, 4-H); 8.38 (s, 1H, 2-H); 8.00 (s, 1H, Ind); 7.96 (s, 1H, Ind); 7.59–7.47 (m, 3H, Ar); 7.07 (t, 1H, Ar); 4.14–4.10 (m, 1H, CH); 2.87 (d, 2H, Pip); 2.21–2.31 (s, 3H, CH_3_); 2.08–1.98 (m, 6H, Pip). ^13^C NMR: DMSO-*d*_6_ (100 MHz): δ 162.8 (d, *J* = 242 Hz; Ph); 147.9 (C-7a); 141.0 (C-6); 137.6 (d, *J* = 8 Hz; Ph); 135.7 (Ind); 130.7 (d, *J* = 9 Hz; Ph); 125.2 (C-2); 124.9 (Ind); 123.3 (C-4); 122.2 (d, *J* = 1 Hz; Ph); 121.7 (C-5); 119.8 (Ind); 117.1 (C-3a); 113.1 (d, *J* = 2 Hz; C-3); 112.6 (d, *J* = 21 Hz; Ph); 112.1 (d, *J* = 21 Hz; Ph); 58.3 (CH); 54.2 (2C, Pip); 45.8 (NCH_3_); 32.1 (2C, Pip). HPLC-MS (ES^+^): CH_3_CN/H_2_O 20:80–95:5, gt: 5 min; rt = 4.15; [M + H]^+^, 376.

#### 3-(3-Methoxyphenyl)-5-(1-(1-methylpiperidin-4-yl)-1*H*-pyrazol-4-yl)-1*H*-pyrro-lo­[2,3-*b*]­pyridine (**5**)

From 3-(3-methoxyphenyl)-5-(1-(1-methylpiperidin-4-yl)-1*H*-pyrazol-4-yl)-1-tosyl-1*H*-pyrrolo­[2,3-*b*]­pyridine (**29**) (75 mg, 0.14 mmol) and 0.4 M NaOH methanolic solution (40 mL). Time of reaction: 4 h. Yield: (42 mg, 85%). mp 134.9–135.4 °C. ^1^H NMR: CDCl_3_ (400 MHz): δ 11.73 (br s, 1H, NH); 8.71 (d, 1H, 6-H); 8.32 (d, 1H, 4-H); 8.28 (d, 1H, Ind); 7.83 (d, 1H, 2-H); 7.51 (d, 1H, Ind); 7.40 (t, 1H, Ar); 7.27 (d, 1H, Ar); 7.21 (s, 1H, Ar); 6.88 (d, 1H, Ar); 4.36–4.28 (m, 1H, Pip); 3.89 (s, 3H, OCH_3_); 3.15–3.11 (m, 2H, Pip); 2.55–2.45 (m, 5H, NCH_3_, Pip); 2.31–2.20 (m, 4H, Pip). ^13^C NMR: CDCl_3_ (100 MHz): δ 160.2 (Ph); 148.6 (C-7a); 141.6 (C-6); 136.6 (Ph); 135.9 (Ind); 130.1 (Ph); 124.7 (C-2); 123.8 (Ind); 123.4 (C-4); 122.2 (C-5); 121.3 (Ph); 119.8 (Ind); 118.6 (C-3a); 116.3 (Ph); 111.6 (C-3); 113.1 (Ph); 111.6 (Ph); 59.7 (CH); 55.5 (OCH_3_); 55.1 (2C, Pip); 46.0 (CH_3_); 32.6 (2C, Pip). HPLC-MS (ES^+^): CH_3_CN/H_2_O 20:80–95:5, gt: 5 min; rt = 4.12; [M + H]^+^, 388.

#### 3-(3,5-Dichlorophenyl)-5-(1-(1-methylpiperidin-4-yl)-1*H*-pyrazol-4-yl)-1*H*-py-rrolo­[2,3-*b*]­pyridine (**6**)

From 3-(3,5-dichlorophenyl)-5-(1-(1-methylpiperidin-4-yl)-1*H*-pyrazol-4-yl)-1-tosyl-1*H*-pyrrolo­[2,3-*b*]­pyridine (**30**) (62 mg, 0.11 mmol) and 0.4 M NaOH methanolic solution (20 mL). Time of reaction: 4 h. Yield: (28 mg, 64%). mp 219.8–220.5 °C. ^1^H NMR: DMSO-*d*_6_ (400 MHz): δ 12.13 (br s, 1H, NH); 8.56 (br s, 1H, 6-H); 8.36 (br s, 2H, 4-H, Ind); 8.07 (s, 1H, 2-H); 7.99 (s, 1H, Ind); 7.78 (br s, 2H, Ar); 7.44 (s, 1H, Ar); 4.13 (br s, 1H, CH); 3.32 (br s, 2H, Pip); 2.87 (br s, 2H, Pip); 2.21 (br s, 2H, Pip); 2.03 (br s, 5H, Pip, NCH_3_). ^13^C NMR: DMSO-*d*_6_ (100 MHz): δ 147.9 (C-7a); 141.3 (C-6); 138.9 (Ph); 135.8 (Ind); 134.6 (2C, Ph); 126.4 (C-2); 125.1 (Ph); 124.8 (Ind); 124.4 (2C, Ph); 123.2 (C-4); 122.0 (C-5); 119.6 (Ind); 116.8 (C-3a); 111.6 (C-3); 58.3 (CH); 54.2 (2C, Pip); 45.8 (NCH_3_); 32.1 (2C, Pip). HPLC-MS (ES^+^): CH_3_CN/H_2_O 15:85–95:5, gt: 5 min; rt = 4.71; [M + H]^+^, 426/428.

#### 3-(2-Fluoro-5-methoxy-4-methylphenyl)-5-(1-(1-methylpiperidin-4-yl)-1*H*-py-razol-4-yl)-1*H*-pyrrolo­[2,3-*b*]­pyridine (**7**)

From 3-(2-fluoro-5-methoxy-4-methylphenyl)-5-(1-(1-methylpiperidin-4-yl)-1*H*-pyrazol-4-yl)-1-tosyl-1*H*-pyrrolo­[2,3-*b*]­pyridine (**31**) (66 mg, 0.12 mmol) and 0.4 M NaOH methanolic solution (20 mL). Time of reaction: 4 h. Yield: (40 mg, 83%). mp 226.8–227.5 °C. ^1^H NMR: DMSO-*d*_6_ (400 MHz): δ 11.93 (br s, 1H, NH); 8.55 (d, 1H, 6-H); 8.31 (s, 1H, Ind); 8.17 (t, 1H, 4-H); 7.93 (s, 1H, Ind); 7.72 (s, 1H, 2-H); 7.15–7.11 (m, 2H, Ar); 4.15–4.07 (m, 1H, CH); 3.87 (s, 3H, OCH_3_); 2.86 (d, 2H, Pip); 2.20 (s, 3H, CH_3_); 2.20 (NCH_3_); 2.08–1.96 (m, 6H, Pip). ^13^C NMR: DMSO-*d*_6_ (100 MHz): δ 153.6 (d, *J* = 1 Hz; Ph); 152.8 (d, *J* = 235 Hz; Ph); 147.4 (C-7a); 140.9 (C-6); 135.5 (Ind); 125.9 (d, *J* = 5 Hz; C-2); 125.1 (d, *J* = 5 Hz; Ph); 124.7 (Ind); 123.6 (d, *J* = 4 Hz; C-4); 121.3 (C-5); 119.8 (Ind); 119.6 (d, *J* = 7 Hz; Ph); 118.0 (C-3a); 117.6 (d, *J* = 14 Hz; Ph); 111.2 (d, *J* = 4 Hz; Ph); 108.5 (C-3); 59.3 (CH); 55.8 (OCH_3_); 54.2 (2C, Pip); 45.8 (NCH_3_); 32.1 (2C, Pip); 15.7 (CH_3_). HPLC-MS (ES^+^): CH_3_CN/H_2_O 15:85–95:5, gt: 5 min; rt = 4.52; [M + H]^+^, 420.

#### 3-(3-Fluorophenyl)-5-(1-(piperidin-4-yl)-1*H*-pyrazol-4-yl)-1*H*-pyrrolo­[2,3-*b*]­py-ridine (**8**)

From 3-(3-fluorophenyl)-5-(1-(piperidin-4-yl)-1*H*-pyrazol-4-yl)-1-tosyl-1*H*-pyrrolo [2,3-*b*]­pyridine (**24**) (48 mg, 0.09 mmol) and 0.4 M NaOH in MeOH (20 mL). Time of reaction: 70 min. Yield: (28 mg, 89%). mp 146.8–147.6 °C. ^1^H NMR: DMSO-*d*_6_ (400 MHz): δ 12.02 (br s, 1H, NH); 8.56 (d, 1H, 6-H); 8.41 (d, 1H, 4-H); 8.36 (s, 1H, Ind); 7.99 (s, 1H, Ind); 7.96 (s, 1H, 2-H); 7.65 (d, 1H, Ar); 7.60–7.57 (m, 1H, Ar); 7.51–7.45 (m, 1H, Ar); 7.09–7.04 (m, 1H, Ar); 4.24–4.16 (m, 1H, CH); 3.07–3.03 (m, 2H, Pip); 2.63–2.56 (m, 2H, Pip); 2.02–1.98 (m, 2H, Pip); 1.88–1.78 (m, 2H, Pip). ^13^C NMR: DMSO-*d*_6_ (100 MHz): δ 162.8 (d, *J* = 244 Hz; Ph); 147.9 (C-7a); 141.0 (C-6); 137.6 (d, *J* = 8 Hz; Ph); 135.6 (Ind); 130.7 (d, *J* = 9 Hz; Ph); 125.2 (C-2); 124.7 (Ind); 123.3 (C-4); 122.2 (d, *J* = 2 Hz; Ph); 121.8 (C-5); 119.7 (Ind); 117.1 (C-3a); 113.0 (d, *J* = 3 Hz; C-3); 112.5 (d, *J* = 22 Hz; Ph); 112.1 (d, *J* = 21 Hz; C-3); 59.4 (CH); 45.2 (2C, Pip); 33.7 (2C, Pip). HPLC-MS (ES^+^): CH_3_CN/H_2_O 20:80–95:5, gt: 5 min; rt = 1.68; [M + H]^+^, 362.

#### 3-(3-Methoxyphenyl)-5-(1-(piperidin-4-yl)-1*H*-pyrazol-4-yl)-1*H*-pyrrolo­[2,3-*b*]­pyridine (**9**)

From 3-(3-methoxyphenyl)-5-(1-(piperidin-4-yl)-1*H*-pyrazol-4-yl)-1-tosyl-1*H*-pyrrolo­[2,3-*b*]­pyridine (**25**) (50 mg, 0.09 mmol) and 0.4 M NaOH in MeOH (20 mL). Time of reaction: 70 min. Yield: (25 mg, 74%). mp 246.8–247.3 °C. ^1^H NMR: DMSO-*d*_6_ (400 MHz): δ 11.91 (br s, 1H, NH); 8.55 (d, 1H, 6-H); 8.40–8.34 (m, 2H, 4-H, Ind); 7.97 (m, 1H, 2-H); 7.87 (s, 1H, Ind); 7.38–7.36 (m, 2H, Ar); 7.26 (s, 1H, Ar); 6.86–6.83 (m, 1H, Ar); 4.23–4.16 (m, 1H, CH); 3.89 (s, 3H, CH_3_); 3.15–3.03 (m, 2H, Pip); 2.63–2.60 (m, 2H, Pip); 2.12–1.96 (m, 4H, Pip). ^13^C NMR: DMSO-*d*_6_ (100 MHz): δ 159.9 (Ph); 147.9 (C-7a); 141.2 (C-6); 136.6 (Ph); 135.7 (Ind); 130.2 (Ph); 124.9 (C-2); 124.6 (Ind); 123.6 (Ph); 121.8 (C-4); 119.9 (C-5); 119.1 (Ind); 117.6 (C-3a); 114.5 (Ph); 111.9 (Ph); 111.6 (C-3); 59.4 (CH); 55.3 (OCH_3_); 45.1 (2C, Pip); 33.6 (2C, Pip). HPLC-MS (ES^+^): CH_3_CN/H_2_O 20:80–95:5, gt: 5 min; rt = 4.03; [M + H]^+^, 374.

#### 3-(3,5-Dichlorophenyl)-5-(1-(piperidin-4-yl)-1*H*-pyrazol-4-yl)-1*H*-pyrrolo­[2,3-*b*]­pyridine (**10**)

From 3-(3,5-dichlorophenyl)-5-(1-(piperidin-4-yl)-1*H*-pyrazol-4-yl)-1-tosyl-1*H*-pyrrolo­[2,3-*b*]­pyridine (**26**) (80 mg, 0.14 mmol) and 0.4 M NaOH in MeOH (20 mL). Time of reaction: 4 h. Yield: (31 mg, 71%). mp 271.1–271.9 °C. ^1^H NMR: DMSO-*d*_6_ (400 MHz): δ 12.08 (br s, 1H, NH); 8.56 (d, 1H, 6-H); 8.36 (d, 1H, 4-H); 8.34 (s, 1H, Ind); 8.07 (s, 1H, 2-H); 7.99 (s, 1H, Ind); 7.78 (d, 2H, Ar); 7.44 (s, 1H, Ar); 4.23–4.17 (m, 1H, CH); 3.05 (d, 2H, Pip); 2.59 (t, 2H, Pip); 1.99 (d, 2H, Pip); 1.84–1.80 (m, 2H, Pip). ^13^C NMR: DMSO-*d*_6_ (100 MHz): δ 148.0 (C-7a); 141.3 (C-6); 138.9 (Ph); 135.7 (Ind); 134.6 (2C, Ph); 126.5 (C-2); 124.9 (Ph); 124.7 (Ind); 124.4 (2C, Ph); 123.2 (C-4); 122.0 (C-5); 119.6 (Ind); 116.9 (C-3a); 111.5 (C-3); 59.4 (CH); 45.2 (2C, Pip); 33.7 (2C, Pip). HPLC-MS (ES^+^): CH_3_CN/H_2_O 15:85–95:5, gt: 5 min; rt = 4.69; [M + H]^+^, 412/414.

#### 3-(2-Fluoro-5-methoxy-4-methylphenyl)-5-(1-(piperidin-4-yl)-1*H*-pyrazol-4-yl)-1*H*-pyrrolo­[2,3-*b*]­pyridine (**11**)

From 3-(2-fluoro-5-methoxy-4-methyl phenyl)-5-(1-(piperidin-4-yl)-1*H*-pyrazol-4-yl)-1-tosyl-1*H*-pyrrolo­[2,3-*b*]­pyridine (**27**) (70 mg, 0.12 mmol) and 0.4 M NaOH in MeOH (20 mL). Time of reaction: 4 h. Yield: (37 mg, 75%). mp 247.9–248.5 °C. ^1^H NMR: DMSO-*d*_6_ (400 MHz): δ 11.96 (br s, 1H, NH); 8.56 (d, 1H, 6-H); 8.29 (s, 1H, Ind); 8.17 (t, 1H, 4-H); 7.92 (s, 1H, Ind); 7.72 (s, 1H, 2-H); 7.15–7.13 (m, 2H, Ar); 4.22–4.14 (m, 1H, CH); 3.87 (s, 3H, OCH_3_); 3.04 (d, 2H, Pip); 2.59 (t, 2H, Pip); 2.20 (s, 3H, CH_3_); 1.98 (d, 2H, Pip); 1.86–1.76 (m, 2H, Pip). ^13^C NMR: DMSO-*d*_6_ (100 MHz): δ 153.6 (d, *J* = 1 Hz; Ph); 152.8 (d, *J* = 235 Hz; Ph); 147.4 (C-7a); 141.0 (C-6); 135.4 (Ind); 125.8 (d, *J* = 4 Hz; C-2); 125.1 (d, *J* = 8 Hz; Ph); 124.5 (Ind); 123.6 (d, *J* = 4 Hz; C-4); 121.4 (C-5); 119.7 (Ind); 119.6 (d, *J* = 15 Hz, Ph); 118.0 (C-3a); 117.6 (d, *J* = 14 Hz; Ph); 111.2 (d, *J* = 4 Hz; Ph); 108.6 (C-3); 59.3 (CH); 55.8 (OCH_3_); 45.2 (2C, Pip); 33.6 (2C, Pip); 15.7 (CH_3_). HPLC-MS (ES^+^): CH_3_CN/H_2_O 15:85–95:5, gt: 5 min; rt = 4.49; [M + H]^+^, 406.

### Biological Studies

#### Inhibition of Human DYRK1A Kinase

The ADP-Glo+ DYRK1A/DYRK1B Kinase Enzyme Systems from Promega (no. catalog VA7425 AND VA7428, respectively) was used to screen compounds for activity against DYRK1A AND DYRK1B. ATP and other reagents were purchased from Sigma-Aldrich (St. Louis, MO). The assays were performed in a buffer solution using 96-well plates. The compound to be tested (5 μL, 40 μM dissolved in 4% DMSO) was added to each well followed by ATP (5 μL, final concentration in the well 10 μM), DYRKtidE (5 μL, 4 μg/well) and the enzyme (5 μL, 25 ng/well). It was then allowed to incubate for 60 min at room temperature and ADP-Gloreagent (20 μL) was added allowing it to incubate again for 40 min at room temperature. After the incubation, the kinase detection agent (40 μL) was added and allowed to incubate for 30 min at room temperature. Finally, the luminescence was recorded using a FLUOstar Optima (BMG Labtechnologies GmbH, Offenburg, Germany) multimode reader. The inhibition activities were calculated based on the maximum activity measured in the absence of an inhibitor. Experiments were performed in triplicate. Dose–response curves for IC_50_ determination of DYRK1A and DYRK1B of harmine and compounds **1**, **5–11** are shown in the Supporting Information.

#### Cell Culture

The mouse microglial BV2 cell line was propagated using DMEM, 10% FBS, 1% streptomycin–penicillin, under humidified 5% CO_2_ and 95% air. On attaining semiconfluence, cells were treated with 400 ng/mL of LPS for 24 h. Some cultures were pretreated for 1 h with the different compounds at several concentrations ranging from 1 to 10 μM. After treatment, cultures were processed for cell viability and nitrite production. Experiments were performed in triplicate. BV2 cells, microglial cells derived from C57/BL6 murine. These cells were immortalized using the v-raf/v-myc carrying J2 retrovirus, resulting in a stable cell line. The cells are commercially available from https://www.cytion.com/BV2-Cells/305156.

#### Cell Viability

Cell viability was determined using the MTT assay, which measures the reduction of 3-(4,5-dimethylthiazol-2-yl)-2,5-diphenyltetrazolium bromide (MTT) to formazan crystals. Briefly, the MTT solution (2 mg/mL) was added to each well and incubated at 37 °C for 2 h. After removing the culture medium, 100 μL of dimethyl sulfoxide was added to each well to dissolve the formazan. The optical density was measured at 532 nm using a microplate reader. The absorbance of the control group was considered as 100% of the cell viability.

#### Nitrite Determination

The assessment of NO production involved quantifying nitrite levels, one of the end products of NO oxidation, through a procedure based on the diazotization of nitrite by sulfanilic acid (Griess reaction). Upon reaching semiconfluence, cells were exposed to 400 ng/mL of LPS for 24 h. Before this, certain cultures were pretreated with various compounds at concentrations ranging from 0.5 to 10 μM. Following a 24-h incubation period, 50 μL aliquots of the samples were combined with 50 μL of Griess reagent in 96-well plates, and the mixture was allowed to incubate at room temperature for 10 min. The absorbance of the resulting mixture was then measured at 520 nm using a microplate reader.

#### Measurement of the Antioxidant Effect of the Compounds

The antioxidant activity of the newly synthesized compounds was assessed using the oxygen radical absorbance capacity (ORAC) in vitro assay^75^. The FLUOstar Optima plate reader (BMG Labtech GmbH, Offenburg, Germany) was employed, with excitation at 485 nm and emission at 520 nm. 2,2′-Azobis­(amidinopropane) dihydrochloride (AAPH), (±)-6-hydroxy-2,5,7,8-tetramethylchromane-2-carboxylic acid (trolox), and fluorescein (FL) were procured from Sigma-Aldrich. The assay was conducted in 75 mM phosphate buffer (pH = 7.4) with a final reaction volume of 200 μL. Each well of black 96-well plates contained 25 μL of the antioxidant sample and 150 μL of fluorescein (10 nM). After preincubation at 37 °C for 30 min, 25 μL of a 240 mM AAPH solution was rapidly added using a multichannel pipet. Fluorescence measurements were taken every 90 s for 90 min, with the plate agitated before each reading. The compounds were tested at four concentrations (10–1 μM). A blank containing FL and AAPH in phosphate buffer, as well as four concentrations of trolox (10–1 μM) served as controls. All reactions were performed in duplicate, with at least three independent tests per compound. Data were exported for analysis, plotting absorbance versus time. The area under the fluorescence decay curve (AUC) was calculated for each sample. ORAC values were derived from the AUC values and expressed as Trolox equivalents.^75^

#### PAMPA–BBB Assay

The brain penetration of active compounds was assessed using a parallel artificial membrane permeability assay (PAMPA).^47^ Eleven drugs with known blood–brain barrier (BBB) permeability—Hydrocortisone, testosterone, imipramine, piroxicam, promazine, clonidine, desipramine, ofloxacin, aldosterone, verapamil, and caffeine—were included in each experiment to validate the analysis.

The compounds were dissolved in a 70/30 PBS pH = 7.4 buffer/ethanol solution at a concentration that ensured appropriate absorbance values in the UV–vis light spectrum. A 5 mL volume of these solutions was filtered using PDVF membrane filters (30 mm diameter, 0.45 μ m pore size).

The acceptor 96-well microplate (MultiScreen 96-well Culture Tray clear, Merck Millipore) was filled with 200 μL of PBS/ethanol (70/30). The donor 96-well filtrate plate (Multiscreen IP Sterile Plate PDVF membrane, 0.45 μ m pore size, Merck Millipore) was coated with 4 μL of porcine brain lipid (Spectra 2000) in dodecane (20 mg mL^–1^).

After 5 min, 200 μL of each compound solution were added. The donor plate was then carefully placed onto the acceptor plate to form a “sandwich,” which was left undisturbed for 3 h at 25 °C. During this time, the compounds diffused from the donor plate through the brain lipid membrane into the acceptor plate.

After incubation, the donor plate was removed. The concentrations of the compounds and commercial drugs were determined by measuring absorbance in the donor wells (before incubation) and the acceptor wells (after incubation) using a CLARIOstar microplate reader (BMG LABTECH). Each sample was analyzed at five wavelengths in four replicates and two independent experiments.

The permeability coefficient (*P*_e_) of each drug, in centimeters per second, was calculated using the following formula

$$P_{e}=\frac{V_{d}\cdot V_{r}}{\left(V_{d}-V_{r}\right)\cdot s\cdot t}\cdot \frac{100\cdot V_{d}}{100\cdot V_{d}-\%\;T\left(V_{d}-V_{r}\right)}$$

$$\%\;T=\frac{V_{r}\cdot A_{r}}{A_{d}\cdot V_{d}}\cdot 100$$

where *V*_d_ and *V*_r_ are the volumes of the donor and receptor solutions (0.2 cm^3^), *s* is the membrane area (0.2642 cm^2^), *t* is the incubation time (3 h = 10,800 s), *A*_r_ is the absorbance of the receptor plate after the experiment, and *A*_d_ is the absorbance in the donor compartment before incubation. The results obtained for quality control drugs were compared with permeability data from the literature. The linear correlation between experimental and literature permeability values was used to classify compounds as those capable of crossing the BBB by passive permeation (CNS+, correlating with a bibliographic *P*_e_ > 4) and those that cannot (CNS–, correlating with a bibliographic *P*_e_ < 2). Compounds that correlate with reported *P*_e_ values between 2 and 4 × 10^–6^ cm s^–1^ are classified as CNS+/–.

## Supplementary Material



## Notes

The top 50 *de novo* molecules designed with AI. Virtual chemical library of derivatives of compound **1**. Structural characterization and purity of the compounds: ^1^H NMR and ^13^C NMR spectral information, elemental analysis data, HPLC chromatograms. Biological assays computational studies: ADME prediction, docking studies and DFT analysis. Molecular formula strings (ZIP)

## Abbreviations

AI: artificial intelligence
AD: Alzheimer disease
ATP: adenosine triphosphate
CADD: computer-aided drug design
DDR1: discoidin domain receptor 1 kinase
DMPNN: directed message-passing neural network
DYRK1A: dual-specificity tyrosine phos-phorylation-regulated kinase 1 A
EC_50_: half maximal effective concentration
EVS: explained variance score
FDA: Food and Drug Administration
GNNs: graph neural networks
GPs: Gaussian processes
HGG: hierarchical graph generation
HPLC: high performance liquid chromatography
IC_50_: half-maximal inhibitory concentration
*K*_i_: inhibitory constant
*K*_d_: dissociation constant
KNN: K-nearest neighbors
MAE: mean absolute error
M3CMLP: multilayer perceptron
QSAR: quantitative structure–activity relationship
PDB: protein Database
QED: quantitative estimate of druglikeness
*R* ^2^: coefficient of determination or *R*-squared
RFs: random forest
RMSE: root-mean-square error
RGA: reinforced genetic algorithm
SBVS: structure-based virtual screening
SPGNN: subgraph pattern GNN
SVR: support vector regressor
VS: virtual screeening
SARS CoV 2: severe acute respiratory syndrome coronavirus 2
XGBoost: extreme gradient boosting
