# Correction to “AI-Driven *De Novo* Design and Development of Nontoxic DYRK1A Inhibitors”

**DOI:** 10.1021/acs.jmedchem.5c02889

**Published:** 2025-12-08

**Authors:** Eduardo González García, Pablo Varas Pardo, Pedro González-Naranjo, Eugenia Ulzurrun, Guillermo Marcos-Ayuso, Concepción Pérez, Juan A. Páez, David Rios Insua, Simón Rodríguez Santana, Nuria E. Campillo

**Affiliations:** † Instituto de Ciencias Matemáticas (ICMAT-CSIC), C/Nicolás Cabrera, 13-15, 28049 Madrid, Spain; ‡ Altenea Biotech S.L., C/Alfonso XII, 46, 28014 Madrid, Spain; § Instituto de Química Médica (IQM-CSIC), C/Juan de la Cierva, 3, 28006 Madrid, Spain; ∥ Centro de Investigaciones Biológicas Margarita Salas (CIB Margarita Salas-CSIC), C/Ramiro de Maeztu, 9, 28040 Madrid, Spain; ⊥ Institute for Research in Technology (IIT), ICAI School of Engineering, Universidad Pontificia Comillas (ICAI) - IIT, C/Alberto Aguilera, 25, 28015 Madrid, Spain; ∇ Escuela de Doctorado, Universidad Autónoma de Madrid, 28049 Madrid, Spain

This Addition and Correction updates the authors name Pablo Varas (as originally published) to Pablo Varas Pardo, reflecting the author’s full legal name.

It also updates the author’s affiliation information to include the Escuela de Doctorado, Universidad Autónoma de Madrid, Madrid, Spain.

The affiliation for Simón Rodríguez Santana has also been updated.

No other authors’ names or affiliations are affected.

