# Supplementary material for: AI-Driven De Novo Design and Development of Nontoxic DYRK1A Inhibitors
Source: J Med Chem. 2025 May 3;68(10):10346–64. doi: 10.1021/acs.jmedchem.5c00512 (PMC12105006; doi:10.1021/acs.jmedchem.5c00512)
Supplement: Supplementary file 1 [file jm5c00512_si_001.zip › Supporting Information/SI_all.pdf]

Supporting Information

AI-driven *de-novo* design and development of non-toxic DYRK1A inhibitors

Eduardo González García,[†,⊥] Pablo Varas,[‡,†,⊥] Pedro González-Naranjo,[¶] Eugenia Ulzurrun,[§,†] Guillermo Marcos- Ayuso,[‡] Concepción Pérez,[¶] Juan A. Páez,[¶] David Rios Insua,[†] Simón Rodríguez Santana,[*,||] and Nuria E. Campillo[*,§]

†Instituto de Ciencias Matemáticas (ICMAT-CSIC). C/ Nicolás Cabrera, 13-15, 28049 Madrid, Spain.

‡Altenea Biotech S.L. C/ Alfonso XII, 46, 28014 Madrid, Spain.

¶Instituto de Química Médica (IQM-CSIC). C/ Juan de la Cierva, 3, 28006 Madrid, Spain.

§Centro de Investigaciones Biológicas Margarita Salas (CIB Margarita Salas-CSIC). C/Ramiro de Maeztu, 9, 28040 Madrid, Spain.

||Universidad Pontificia Comillas (ICAI-UPC). C/ Alberto Aguilera, 25, 28015 Madrid, Spain.

⊥These authors contributed equally.

E-mail: srsantana@icai.comillas.edu; nuria.campillo@csic.es

Table of Contents

The top 50 de-novo molecules designed with AI

Virtual chemical library of derivatives of compound 1

^{1}H-NMR and ^{13}C-NMR Spectral Information

Elemental Analysis

HPLC Chromatograms

Biological Assays

ADMET/Tox Properties

Docking studies

DFT studies

Table S1. The top 50 de-novo molecules designed with A

Ranking	Smiles	Estimated pChEMBL Value	Estimated IC_{50} Value	Docking Score (kcal/mol)
I	CN1CCC(n2cc(-c3cnc4[nH]cc(-c5cccc(F)c5)c4c3)cn2)CC1	7,917	0,01	-13,99
II	COCCN(C)Cc1cncc(C(=O)Nc2c[nH]nc2C(=O)Nc2ccc(CNCCNC(C)C)nc2)c1	8,297	0,01	-13,23
III	CN1CCN(Cc2ccc(Nc3ncc4ccc(-c5cccnc5)cc4n3)nc2)CC1	7,781	0,02	-12,74
IV	CC(C)C(=O)Nc1cncc(-c2cncc(-c3cncc(C4(O)CCN(C)C4)c3)c2)c1	8,170	0,01	-12,54
V	CN1CCN(Cc2cncc(-c3cncc(C(=O)Nc4cncc(-c5cc(F)cc(-c6nn[nH]n6)c5)c4)c3)c2)CCC1=O	8,087	0,01	-12,38
VI	O=C(Nc1cc(CN2CCOCC2)ccn1)c1n[nH]c2cc(F)ccc12	7,863	0,01	-12,00
VII	CCN1CCN(Cc2ccc(C(=O)Nc3cncc(-c4c[nH]c5ccc(C(=O)O)cc45)c3)cc2)CC1	8,229	0,01	-11,75
VIII	CN1CCN(Cc2csc(C(=O)Nc3cccc(C(=O)NC(CCN)c4ccccc4)c3)c2)CC1	8,408	0,00	-11,69
IX	CN(C)CCCNc1cccc(C(=O)Nc2cncc(-c3cccc(CN4CCC(F)(F)C4)c3)c2)c1	8,001	0,01	-11,46
X	CN1CCC(c2ccc(Nc3ccnc4[nH]c(-c5cnn(C6CCN(C)CC6)c5)cc34)cn2)CC1	8,074	0,01	-11,34
XI	CN1CCC(c2cc(C(=O)Nc3cncc(-c4cccc(F)c4)c3)n[nH]2)CC1	8,175	0,01	-11,22
XII	CN(C)CCNCc1cc(F)cc(-c2cnc3[nH]c(C4CCCCN4)nc3c2)c1	7,888	0,01	-10,99
XIII	O=C(Nc1cccnc1)c1cc(C(=O)Nc2c(O)ccc(C(=O)O)c2O)[nH]n1	7,817	0,02	-10,92
XIV	FC1(F)CCN(Cc2cncc(-c3cncc(-c4cncc(CN5CCC(F)(F)C5)c4)c3)c2)C1	7,914	0,01	-10,85
XV	CC(C)N(Cc1cncc(-c2cncc(-c3cncc(C(=O)Nc4cccc(C(=O)NC5CCN(C)CC5)c4)c3)c2)c1)C(C)C	7,827	0,01	-10,80
XVI	CC(C)N(C)Cc1cncc(-c2cncc(NC(=O)c3cnn(C4CCN(C(C)C)CC4)c3)c2)c1	7,852	0,01	-10,72
XVII	CN1CCN(CC(=O)Nc2ncc3ccc(-c4cn[nH]c4)cc3n2)CC1	7,893	0,01	-10,70
XVIII	CCC1(Cn2cc(C(=O)Nc3cc4cc(-c5cccnc5)cnc4cn3)cn2)COC1	8,383	0,00	-10,66
XIX	CN1CCN(c2cncc(-c3n[nH]c4ncc(-c5cnccn5)cc34)n2)CC1	8,236	0,01	-10,64
XX	CC1CN(Cc2cc(F)cc(-c3cnc4c(C=CC5(O)CNC5)cnn4c3)c2)CC(C)O1	8,465	0,00	-10,52
XXI	CN1CCC(c2cc(C(=O)Nc3cncc(-c4ccccc4F)c3)ccn2)CC1	8,221	0,01	-10,40
XXII	CCCCc1ncc(-c2ccnc(Nc3cncc(-c4cccc(CN5CCCC5)c4)c3)c2)n1C	8,222	0,01	-10,38
XXIII	CN(C)CCC(NC(=O)c1ccc(NC(=O)c2cc(C3CCN(C)CC3)[nH]n2)cn1)c1ccccc1	7,893	0,01	-10,32
XXIV	CN1CCN(c2cc(-c3c[nH]c4ncc(-c5ccoc5)cc34)ccn2)CC1	8,044	0,01	-10,06
XXV	CN1CCC(n2cc(-c3csc(-c4cncc(-c5cncc(CN6CCCCC6)c5)c4)c3)cn2)CC1	8,070	0,01	-9,98
XXVI	CN1CCC(n2cc(C(=O)Nc3ccnc4cc(Cl)ccc34)cn2)CC1	8,172	0,01	-9,91
XXVII	CN1CCN(c2cc(-c3n[nH]c4c(-c5ccncc5)cncc34)ccn2)CC1	8,158	0,01	-9,91
XXVIII	CC(=O)Nc1cc(-c2ccc3cncc(C4CCN(C)CC4)c3c2)ccn1	7,742	0,02	-9,74

XXIX	CN1CCC(n2cc(C(=O)Nc3cc(C(=O)Nc4ccc(F)cc4F)ccc3CN3CCCC3)cn2)CC1	8,081	0,01	-9,56
XXX	CN1CCN(CCNC(=O)c2cc(C(=O)Nc3cccc(-c4cc(F)cc(F)c4)c3)[nH]n2)CC1	8,115	0,01	-9,44
XXXI	CN1CCN(C(=O)Nc2cc3cc(-c4cnccn4)cnc3cn2)CC1	8,007	0,01	-9,37
XXXII	COC(=O)c1cccc(NC(=O)c2cnc3ccc(-c4cncc(CN5CCCCC5)c4)cn23)c1	8,147	0,01	-9,26
XXXIII	Cc1ncccc1-c1ccc2cnc(NC(=O)C3CCN(C)CC3)cc2c1	8,060	0,01	-9,03
XXXIV	O=C(Nc1cc(-c2ccc3[nH]ncc3c2)ccn1)c1ccc2ncn(CC3CC3)c2c1	NA	NA	-8,99
XXXV	CN1CCC(n2cc(-c3n[nH]c4c(-c5cncc(N)c5)cncc34)cn2)CC1	8,137	0,01	-8,97
XXXVI	Cc1nc2ccc(-c3ccncc3)nc2n1-c1cc(F)cc(C(=O)O)c1	8,460	0,00	-8,94
XXXVII	CC(C)Nc1cnc(C(=O)Nc2cn[nH]c2C(=O)Nc2cc(F)cc(CN3CCOCC3)c2)cn1	8,204	0,01	-8,87
XXXVIII	CC(=O)Nc1c(C(=O)Nc2cncc(-<br>c3cc(CN4CCC(C)(C)CC4)cs3)c2)cnc2[nH]cnc12	NA	NA	-8,86
XXXIX	COC(=O)c1cccc(-n2cnc3ccc(-c4cnc(C)n4C)cc32)c1	7,774	0,02	-8,85
XL	CN(C)C1CCN(Cc2cccc(C(=O)Nc3cccc(F)c3)c2)C1	8,073	0,01	-8,75
XLI	CN(C)CCN(C)Cc1cncc(-c2cncc(-c3cncc(NC(=O)C4CCCC4)c3)c2)c1	7,732	0,02	-8,70
XLII	CCNC(=O)c1cncc(-c2cncc(-c3cncc(CN4CCCC4)c3)c2)c1	7,830	0,01	-8,61
XLIII	CN1CCN(c2cc(C(=O)Nc3cncc(-c4cncc(CN5CCCCC5)c4)c3)ccn2)CC1	8,436	0,00	-8,40
XLIV	Cc1[nH]c(C(=O)Nc2cncc(-c3cncc(CN4CCC(F)(F)CC4)c3)c2)c(C)c1C	8,270	0,01	-8,24
XLV	Cc1nc(C(=O)O)c2[nH]c(-c3cnn(C4CCNCC4)c3)cc2n1	8,791	0,00	-8,21
XLVI	CC1CN(c2cc(C(=O)Nc3cncc(C4CCNCC4F)c3)ccn2)CC(C)O1	8,128	0,01	-7,98
XLVII	CN(C)CC(=O)Nc1ccc(-n2cc(C(=O)Nc3cccc(CN4CCCC4)c3)cn2)cc1	7,831	0,01	-7,74
XLVIII	O=C(Nc1cc(-c2nnn[nH]2)ccc1C(=O)O)c1cnccn1	8,124	0,01	-7,50
XLIX	CN1CCN(c2cncc(C(=O)Nc3ccc(C(=O)NC(CCN)c4ccccc4)cc3)n2)CC1	8,468	0,00	-7,40
L	Cc1ncc(-c2cc3c(C(=O)NC(CCCNC(=N)N)C(=O)Nc4cc5cc(-<br>c6cnn(C)c6)ccc5cn4)n[nH]c3cn2)cn1	8,001	0,01	-7,30

Table S2. Virtual chemical library of derivatives of compound 1

Comp.	SMILES	IC_{50}
1	Fc1cc(C2=CNc3c2cc(C4=CN(N=C4)C5CCN(C)CC5)cn3)ccc1	0,01
5	CN(CC1)CCC1N2N=CC(c3cnc(NC=C4c5cccc(OC)c5)c4c3)=C2	0,07
	Clc1cccc(C2=CNc3ncc(C4=CN(C5CCN(C)CC5)N=C4)cc32)c1	0,08
	Cc1cccc(C2=CNc3ncc(C4=CN(C5CCN(C)CC5)N=C4)cc32)c1	0,08
	CN(CC1)CCC1N2N=CC(c3cnc(NC=C4c5cccc(N(C)C)c5)c4c3)=C2	0,15
	CN(CC1)CCC1N2N=CC(c3cnc(NC=C4c5cccc([N+]([O-])=O)c5)c4c3)=C2	0,12
	CN(CC1)CCC1N2N=CC(c3cnc(NC=C4c5cccc(C(F)(F)F)c5)c4c3)=C2	0,14
	Fc1cccc(C2=CNc3ncc(C4=CN(C5CCN(C)CC5)N=C4)cc32)c1Cl	0,25
	Fc1cccc(C2=CNc3ncc(C4=CN(C5CCN(C)CC5)N=C4)cc32)c1F	0,13
	Fc1cccc(C2=CNc3ncc(C4=CN(C5CCN(C)CC5)N=C4)cc32)c1OC	0,22
	Fc1cccc(C2=CNc3ncc(C4=CN(C5CCN(C)CC5)N=C4)cc32)c1C	0,49
	Fc1cccc(C2=CNc3ncc(C4=CN(C5CCN(C)CC5)N=C4)cc32)c1C(F)(F)F	0,49
	Fc1c(OC)ccc(C2=CNc3ncc(C4=CN(C5CCN(C)CC5)N=C4)cc32)c1	0,46
	Fc1c(F)ccc(C2=CNc3ncc(C4=CN(C5CCN(C)CC5)N=C4)cc32)c1	0,12
	Fc1c(C)ccc(C2=CNc3ncc(C4=CN(C5CCN(C)CC5)N=C4)cc32)c1	0,24
	Fc1c(Cl)ccc(C2=CNc3ncc(C4=CN(C5CCN(C)CC5)N=C4)cc32)c1	0,28
	Fc1c([N+]([O-])=O)ccc(C2=CNc3ncc(C4=CN(C5CCN(C)CC5)N=C4)cc32)c1	0,14
	Fc1c(C(F)(F)F)ccc(C2=CNc3ncc(C4=CN(C5CCN(C)CC5)N=C4)cc32)c1	0,20
	Fc1cc(OC)cc(C2=CNc3ncc(C4=CN(C5CCN(C)CC5)N=C4)cc32)c1	0,12
	Fc1cc(F)cc(C2=CNc3ncc(C4=CN(C5CCN(C)CC5)N=C4)cc32)c1	0,12
	Fc1cc(N(C)C)cc(C2=CNc3ncc(C4=CN(C5CCN(C)CC5)N=C4)cc32)c1	0,16
	Fc1cc(C(F)(F)F)cc(C2=CNc3ncc(C4=CN(C5CCN(C)CC5)N=C4)cc32)c1	0,28
	Fc1ccc(Cl)c(C2=CNc3ncc(C4=CN(C5CCN(C)CC5)N=C4)cc32)c1	0,11
	Fc1ccc(F)c(C2=CNc3ncc(C4=CN(C5CCN(C)CC5)N=C4)cc32)c1	0,11
	Fc1ccc(OC)c(C2=CNc3ncc(C4=CN(C5CCN(C)CC5)N=C4)cc32)c1	0,34
	Fc1ccc(C)c(C2=CNc3ncc(C4=CN(C5CCN(C)CC5)N=C4)cc32)c1	0,35
	Fc1ccc(C(F)(F)F)c(C2=CNc3ncc(C4=CN(C5CCN(C)CC5)N=C4)cc32)c1	0,41
	Fc1cc(C)cc(C2=CNc3ncc(C4=CN(C5CCN(C)CC5)N=C4)cc32)c1	0,16
	Clc1cccc(C2=CNc3ncc(C4=CN(C5CCN(C)CC5)N=C4)cc32)c1F	0,23
	Clc1cccc(C2=CNc3ncc(C4=CN(C5CCN(C)CC5)N=C4)cc32)c1Cl	0,15
	Clc1cccc(C2=CNc3ncc(C4=CN(C5CCN(C)CC5)N=C4)cc32)c1C	0,59
	Clc1cccc(C2=CNc3ncc(C4=CN(C5CCN(C)CC5)N=C4)cc32)c1C(F)(F)F	0,72
	Clc1cccc(C2=CNc3ncc(C4=CN(C5CCN(C)CC5)N=C4)cc32)c1OC	0,17
	Clc1c(F)ccc(C2=CNc3ncc(C4=CN(C5CCN(C)CC5)N=C4)cc32)c1	0,78

	Clc1c(Cl)ccc(C2=CNc3ncc(C4=CN(C5CCN(C)CC5)N=C4)cc32)c1	0,34
	Clc1c(C)ccc(C2=CNc3ncc(C4=CN(C5CCN(C)CC5)N=C4)cc32)c1	0,32
	Clc1c(C(F)(F)F)ccc(C2=CNc3ncc(C4=CN(C5CCN(C)CC5)N=C4)cc32)c1	0,73
	Clc1c(OC)ccc(C2=CNc3ncc(C4=CN(C5CCN(C)CC5)N=C4)cc32)c1	0,57
	Clc1c(N(C)C)ccc(C2=CNc3ncc(C4=CN(C5CCN(C)CC5)N=C4)cc32)c1	0,32
	Clc1cc(F)cc(C2=CNc3ncc(C4=CN(C5CCN(C)CC5)N=C4)cc32)c1	0,29
6	Clc1cc(Cl)cc(C2=CNc3ncc(C4=CN(C5CCN(C)CC5)N=C4)cc32)c1	0,06
	Clc1cc(C)cc(C2=CNc3ncc(C4=CN(C5CCN(C)CC5)N=C4)cc32)c1	0,11
	Clc1cc(C(F)(F)F)cc(C2=CNc3ncc(C4=CN(C5CCN(C)CC5)N=C4)cc32)c1	0,24
	Clc1cc(OC)cc(C2=CNc3ncc(C4=CN(C5CCN(C)CC5)N=C4)cc32)c1	0,10
	Cc1cccc(C2=CNc3ncc(C4=CN(C5CCN(C)CC5)N=C4)cc32)c1F	0,09
	Cc1cccc(C2=CNc3ncc(C4=CN(C5CCN(C)CC5)N=C4)cc32)c1C	0,23
	Cc1c(F)ccc(C2=CNc3ncc(C4=CN(C5CCN(C)CC5)N=C4)cc32)c1	0,13
	Cc1c(Cl)ccc(C2=CNc3ncc(C4=CN(C5CCN(C)CC5)N=C4)cc32)c1	0,18
	Cc1c(C)ccc(C2=CNc3ncc(C4=CN(C5CCN(C)CC5)N=C4)cc32)c1	0,10
	Cc1c(C(F)(F)F)ccc(C2=CNc3ncc(C4=CN(C5CCN(C)CC5)N=C4)cc32)c1	0,17
	Cc1c(OC)ccc(C2=CNc3ncc(C4=CN(C5CCN(C)CC5)N=C4)cc32)c1	0,15
	Cc1c([N+]([O-])=O)ccc(C2=CNc3ncc(C4=CN(C5CCN(C)CC5)N=C4)cc32)c1	0,11
	CN(CC1)CCC1N2N=CC(c3cnc(NC=C4c5cc(C)cc(C)c5)c4c3)=C2	0,07
	CN(CC1)CCC1N2N=CC(c3cnc(NC=C4c5cc(C)cc(OC)c5)c4c3)=C2	0,08
	CN(CC1)CCC1N2N=CC(c3cnc(NC=C4c5cc(C)ccc5F)c4c3)=C2	0,08
	CN(CC1)CCC1N2N=CC(c3cnc(NC=C4c5cc(C)ccc5Cl)c4c3)=C2	0,09
	CN(CC1)CCC1N2N=CC(c3cnc(NC=C4c5cc(C)ccc5C)c4c3)=C2	0,18
	CN(CC1)CCC1N2N=CC(c3cnc(NC=C4c5cc(C)ccc5OC)c4c3)=C2	0,17
	CN(CC1)CCC1N2N=CC(c3cnc(NC=C4c5cccc(C(F)(F)F)c5F)c4c3)=C2	0,12
	CN(CC1)CCC1N2N=CC(c3cnc(NC=C4c5cccc(C(F)(F)F)c5Cl)c4c3)=C2	0,25
	CN(CC1)CCC1N2N=CC(c3cnc(NC=C4c5cccc(C(F)(F)F)c5C)c4c3)=C2	0,58
	CN(CC1)CCC1N2N=CC(c3cnc(NC=C4c5ccc(F)c(C(F)(F)F)c5)c4c3)=C2	0,18
	CN(CC1)CCC1N2N=CC(c3cnc(NC=C4c5ccc(C(F)(F)F)c(C(F)(F)F)c5)c4c3)=C2	0,19
	CN(CC1)CCC1N2N=CC(c3cnc(NC=C4c5ccc(OC)c(C(F)(F)F)c5)c4c3)=C2	0,25
	CN(CC1)CCC1N2N=CC(c3cnc(NC=C4c5cc(C(F)(F)F)cc(C)c5)c4c3)=C2	0,20
	CN(CC1)CCC1N2N=CC(c3cnc(NC=C4c5cc(C(F)(F)F)cc(C(F)(F)F)c5)c4c3)=C2	0,30
	CN(CC1)CCC1N2N=CC(c3cnc(NC=C4c5cc(C(F)(F)F)ccc5F)c4c3)=C2	0,80
	CN(CC1)CCC1N2N=CC(c3cnc(NC=C4c5cc(C(F)(F)F)ccc5Cl)c4c3)=C2	0,70
	CN(CC1)CCC1N2N=CC(c3cnc(NC=C4c5cc(C(F)(F)F)ccc5C)c4c3)=C2	1,44
	CN(CC1)CCC1N2N=CC(c3cnc(NC=C4c5cc(C(F)(F)F)ccc5C(F)(F)F)c4c3)=C2	1,53
	CN(CC1)CCC1N2N=CC(c3cnc(NC=C4c5cc(C(F)(F)F)ccc5[N+]([O-])=O)c4c3)=C2	1,58

	CN(CC1)CCC1N2N=CC(c3cnc(NC=C4c5cccc(OC)c5F)c4c3)=C2	0,09
	CN(CC1)CCC1N2N=CC(c3cnc(NC=C4c5cccc(OC)c5Cl)c4c3)=C2	0,11
	CN(CC1)CCC1N2N=CC(c3cnc(NC=C4c5cccc(OC)c5C)c4c3)=C2	0,24
	CN(CC1)CCC1N2N=CC(c3cnc(NC=C4c5ccc(F)c(OC)c5)c4c3)=C2	0,14
	CN(CC1)CCC1N2N=CC(c3cnc(NC=C4c5ccc(Cl)c(OC)c5)c4c3)=C2	0,13
	CN(CC1)CCC1N2N=CC(c3cnc(NC=C4c5ccc(C)c(OC)c5)c4c3)=C2	0,12
	CN(CC1)CCC1N2N=CC(c3cnc(NC=C4c5ccc([N+]([O-])=O)c(OC)c5)c4c3)=C2	0,13
	CN(CC1)CCC1N2N=CC(c3cnc(NC=C4c5cc(C(F)(F)F)cc(OC)c5)c4c3)=C2	0,25
	CN(CC1)CCC1N2N=CC(c3cnc(NC=C4c5cccc([N+]([O-])=O)c5F)c4c3)=C2	0,16
	CN(CC1)CCC1N2N=CC(c3cnc(NC=C4c5cccc([N+]([O-])=O)c5C)c4c3)=C2	0,36
	CN(CC1)CCC1N2N=CC(c3cnc(NC=C4c5cccc([N+]([O-])=O)c5OC)c4c3)=C2	0,13
	CN(CC1)CCC1N2N=CC(c3cnc(NC=C4c5ccc(F)c([N+]([O-])=O)c5)c4c3)=C2	0,21
	CN(CC1)CCC1N2N=CC(c3cnc(NC=C4c5ccc(Cl)c([N+]([O-])=O)c5)c4c3)=C2	0,07
	CN(CC1)CCC1N2N=CC(c3cnc(NC=C4c5ccc(C)c([N+]([O-])=O)c5)c4c3)=C2	0,10
	CN(CC1)CCC1N2N=CC(c3cnc(NC=C4c5ccc(OC)c([N+]([O-])=O)c5)c4c3)=C2	0,13
	CN(CC1)CCC1N2N=CC(c3cnc(NC=C4c5ccc(N(C)C)c([N+]([O-<br>])=O)c5)c4c3)=C2	0,10
	CN(CC1)CCC1N2N=CC(c3cnc(NC=C4c5cc(C)cc([N+]([O-])=O)c5)c4c3)=C2	0,11
	CN(CC1)CCC1N2N=CC(c3cnc(NC=C4c5cc(C(F)(F)F)cc([N+]([O-<br>])=O)c5)c4c3)=C2	0,22
	CN(CC1)CCC1N2N=CC(c3cnc(NC=C4c5cc([N+]([O-])=O)cc([N+]([O-<br>])=O)c5)c4c3)=C2	0,20
	CN(CC1)CCC1N2N=CC(c3cnc(NC=C4c5ccc(F)cc5)c4c3)=C2	0,07
	CN(CC1)CCC1N2N=CC(c3cnc(NC=C4c5ccc(Cl)cc5)c4c3)=C2	0,08
	CN(CC1)CCC1N2N=CC(c3cnc(NC=C4c5ccc(C)cc5)c4c3)=C2	0,08
	CN(CC1)CCC1N2N=CC(c3cnc(NC=C4c5ccc(C(F)(F)F)cc5)c4c3)=C2	0,11
	CN(CC1)CCC1N2N=CC(c3cnc(NC=C4c5ccc(OC)cc5)c4c3)=C2	0,06
	CN(CC1)CCC1N2N=CC(c3cnc(NC=C4c5ccc([N+]([O-])=O)cc5)c4c3)=C2	0,10
	CN(CC1)CCC1N2N=CC(c3cnc(NC=C4c5ccc(N(C)C)cc5)c4c3)=C2	0,07
	CN(CC1)CCC1N2N=CC(c3cnc(NC=C4c5ccc(F)cc5F)c4c3)=C2	0,08
	CN(CC1)CCC1N2N=CC(c3cnc(NC=C4c5ccc(F)cc5Cl)c4c3)=C2	0,06
	CN(CC1)CCC1N2N=CC(c3cnc(NC=C4c5ccc(F)cc5C)c4c3)=C2	0,10
	CN(CC1)CCC1N2N=CC(c3cnc(NC=C4c5ccc(F)cc5C(F)(F)F)c4c3)=C2	0,15
	CN(CC1)CCC1N2N=CC(c3cnc(NC=C4c5ccc(F)cc5OC)c4c3)=C2	0,09
	CN(CC1)CCC1N2N=CC(c3cnc(NC=C4c5ccc(F)cc5[N+]([O-])=O)c4c3)=C2	0,13
	CN(CC1)CCC1N2N=CC(c3cnc(NC=C4c5ccc(Cl)cc5F)c4c3)=C2	0,09
	CN(CC1)CCC1N2N=CC(c3cnc(NC=C4c5ccc(Cl)cc5Cl)c4c3)=C2	0,05
	CN(CC1)CCC1N2N=CC(c3cnc(NC=C4c5ccc(Cl)cc5C)c4c3)=C2	0,11

	CN(CC1)CCC1N2N=CC(c3cnc(NC=C4c5ccc(Cl)cc5C(F)(F)F)c4c3)=C2	0,18
	CN(CC1)CCC1N2N=CC(c3cnc(NC=C4c5ccc(Cl)cc5OC)c4c3)=C2	0,10
	CN(CC1)CCC1N2N=CC(c3cnc(NC=C4c5ccc(Cl)cc5[N+]([O-])=O)c4c3)=C2	0,14
	CN(CC1)CCC1N2N=CC(c3cnc(NC=C4c5ccc(C)cc5F)c4c3)=C2	0,09
	CN(CC1)CCC1N2N=CC(c3cnc(NC=C4c5ccc(C)cc5Cl)c4c3)=C2	0,06
	CN(CC1)CCC1N2N=CC(c3cnc(NC=C4c5ccc(C)cc5C)c4c3)=C2	0,11
	CN(CC1)CCC1N2N=CC(c3cnc(NC=C4c5ccc(C)cc5C(F)(F)F)c4c3)=C2	0,19
	CN(CC1)CCC1N2N=CC(c3cnc(NC=C4c5ccc(C)cc5OC)c4c3)=C2	0,10
	CN(CC1)CCC1N2N=CC(c3cnc(NC=C4c5ccc(C)cc5[N+]([O-])=O)c4c3)=C2	0,13
	CN(CC1)CCC1N2N=CC(c3cnc(NC=C4c5ccc(C(F)(F)F)cc5F)c4c3)=C2	0,12
	CN(CC1)CCC1N2N=CC(c3cnc(NC=C4c5ccc(C(F)(F)F)cc5Cl)c4c3)=C2	0,09
	CN(CC1)CCC1N2N=CC(c3cnc(NC=C4c5ccc(C(F)(F)F)cc5C)c4c3)=C2	0,17
	CN(CC1)CCC1N2N=CC(c3cnc(NC=C4c5ccc(C(F)(F)F)cc5C(F)(F)F)c4c3)=C2	0,20
	CN(CC1)CCC1N2N=CC(c3cnc(NC=C4c5ccc(C(F)(F)F)cc5OC)c4c3)=C2	0,15
	CN(CC1)CCC1N2N=CC(c3cnc(NC=C4c5ccc(OC)cc5F)c4c3)=C2	0,07
	CN(CC1)CCC1N2N=CC(c3cnc(NC=C4c5ccc(OC)cc5Cl)c4c3)=C2	0,05
	CN(CC1)CCC1N2N=CC(c3cnc(NC=C4c5ccc(OC)cc5C)c4c3)=C2	0,10
	CN(CC1)CCC1N2N=CC(c3cnc(NC=C4c5ccc(OC)cc5C(F)(F)F)c4c3)=C2	0,16
	CN(CC1)CCC1N2N=CC(c3cnc(NC=C4c5ccc(OC)cc5OC)c4c3)=C2	0,09
	CN(CC1)CCC1N2N=CC(c3cnc(NC=C4c5ccc(OC)cc5[N+]([O-])=O)c4c3)=C2	0,11
	CN(CC1)CCC1N2N=CC(c3cnc(NC=C4c5ccc([N+]([O-])=O)cc5F)c4c3)=C2	0,10
	CN(CC1)CCC1N2N=CC(c3cnc(NC=C4c5ccc([N+]([O-])=O)cc5Cl)c4c3)=C2	0,08
	CN(CC1)CCC1N2N=CC(c3cnc(NC=C4c5ccc([N+]([O-])=O)cc5C)c4c3)=C2	0,13
	CN(CC1)CCC1N2N=CC(c3cnc(NC=C4c5ccc([N+]([O-])=O)cc5C(F)(F)F)c4c3)=C2	0,18
	CN(CC1)CCC1N2N=CC(c3cnc(NC=C4c5ccc([N+]([O-])=O)cc5OC)c4c3)=C2	0,12
	CN(CC1)CCC1N2N=CC(c3cnc(NC=C4c5cc(F)c(F)c(F)c5)c4c3)=C2	0,25
	CN(CC1)CCC1N2N=CC(c3cnc(NC=C4c5cc(F)c(Cl)c(F)c5)c4c3)=C2	0,36
	CN(CC1)CCC1N2N=CC(c3cnc(NC=C4c5cc(F)c(Cl)c(Cl)c5)c4c3)=C2	0,65
	CN(CC1)CCC1N2N=CC(c3cnc(NC=C4c5cc(F)c(Cl)cc5F)c4c3)=C2	0,27
	CN(CC1)CCC1N2N=CC(c3cnc(NC=C4c5cc(F)c(F)cc5Cl)c4c3)=C2	0,29
	CN(CC1)CCC1N2N=CC(c3cnc(NC=C4c5cc(F)cc(F)c5F)c4c3)=C2	0,17
	CN(CC1)CCC1N2N=CC(c3cnc(NC=C4c5cc(F)cc(Cl)c5Cl)c4c3)=C2	0,25
	CN(CC1)CCC1N2N=CC(c3cnc(NC=C4c5c(F)c(F)c(F)cc5)c4c3)=C2	0,21
	CN(CC1)CCC1N2N=CC(c3cnc(NC=C4c5c(F)c(F)ccc5F)c4c3)=C2	0,25
	CN(CC1)CCC1N2N=CC(c3cnc(NC=C4c5c(F)c(F)ccc5Cl)c4c3)=C2	0,46
	CN(CC1)CCC1N2N=CC(c3cnc(NC=C4c5c(F)c(F)cc(Cl)c5)c4c3)=C2	0,32
	CN(CC1)CCC1N2N=CC(c3cnc(NC=C4c5c(Cl)c(F)c(Cl)cc5)c4c3)=C2	0,16

	CN(CC1)CCC1N2N=CC(c3cnc(NC=C4c5c(Cl)c(F)c(F)cc5)c4c3)=C2	0,30
	CN(CC1)CCC1N2N=CC(c3cnc(NC=C4c5c(Cl)c(F)cc(Cl)c5)c4c3)=C2	0,35
	CN(CC1)CCC1N2N=CC(c3cnc(NC=C4c5c(C)c(F)c(F)cc5)c4c3)=C2	0,38
	CN(CC1)CCC1N2N=CC(c3cnc(NC=C4c5c(C)c(F)c(Cl)cc5)c4c3)=C2	0,68
	CN(CC1)CCC1N2N=CC(c3cnc(NC=C4c5c(F)c(F)c(C)cc5)c4c3)=C2	0,18
	CN(CC1)CCC1N2N=CC(c3cnc(NC=C4c5cc(F)c(C)c(F)c5)c4c3)=C2	0,17
	CN(CC1)CCC1N2N=CC(c3cnc(NC=C4c5cc(F)c(Cl)cc5C)c4c3)=C2	0,66
	CN(CC1)CCC1N2N=CC(c3cnc(NC=C4c5cc(F)c(C)cc5F)c4c3)=C2	0,18
	CN(CC1)CCC1N2N=CC(c3cnc(NC=C4c5cc(F)cc(C)c5Cl)c4c3)=C2	0,10
	CN(CC1)CCC1N2N=CC(c3cnc(NC=C4c5c(C)c(F)ccc5F)c4c3)=C2	0,41
	CN(CC1)CCC1N2N=CC(c3cnc(NC=C4c5c(F)c(F)c(Cl)c(OC)c5)c4c3)=C2	0,04
	CN(CC1)CCC1N2N=CC(c3cnc(NC=C4c5cc(F)c(F)c(OC)c5)c4c3)=C2	0,17
	CN(CC1)CCC1N2N=CC(c3cnc(NC=C4c5c(F)c(F)cc(OC)c5)c4c3)=C2	0,13
	CN(CC1)CCC1N2N=CC(c3cnc(NC=C4c5cc(F)c(F)cc5OC)c4c3)=C2	0,23
	CN(CC1)CCC1N2N=CC(c3cnc(NC=C4c5cc(F)cc(Cl)c5OC)c4c3)=C2	0,31
	CN(CC1)CCC1N2N=CC(c3cnc(NC=C4c5c(F)c(F)ccc5OC)c4c3)=C2	0,37
	CN(CC1)CCC1N2N=CC(c3cnc(NC=C4c5c(OC)c(F)c(F)cc5)c4c3)=C2	1,19
	CN(CC1)CCC1N2N=CC(c3cnc(NC=C4c5c(OC)c(F)ccc5F)c4c3)=C2	0,18
	CN(CC1)CCC1N2N=CC(c3cnc(NC=C4c5c(OC)c(F)cc(F)c5)c4c3)=C2	0,20
	CN(CC1)CCC1N2N=CC(c3cnc(NC=C4c5c(OC)c(F)c(Cl)cc5)c4c3)=C2	0,38
	CN(CC1)CCC1N2N=CC(c3cnc(NC=C4c5c(Cl)c(F)c(OC)cc5)c4c3)=C2	0,51
	CN(CC1)CCC1N2N=CC(c3cnc(NC=C4c5cc(F)c(OC)cc5F)c4c3)=C2	0,19
	CN(CC1)CCC1N2N=CC(c3cnc(NC=C4c5c(F)c(F)c(OC)cc5)c4c3)=C2	0,31
	CN(CC1)CCC1N2N=CC(c3cnc(NC=C4c5cc(F)c(OC)c(F)c5)c4c3)=C2	0,23
	CN(CC1)CCC1N2N=CC(c3cnc(NC=C4c5c(OC)c(F)c(OC)cc5)c4c3)=C2	0,45
	CN(CC1)CCC1N2N=CC(c3cnc(NC=C4c5cc(F)c(F)cc5[N+]([O-])=O)c4c3)=C2	0,28
	CN(CC1)CCC1N2N=CC(c3cnc(NC=C4c5cc(F)c([N+]([O-])=O)c(F)c5)c4c3)=C2	0,34
	CN(CC1)CCC1N2N=CC(c3cnc(NC=C4c5cc(F)c([N+]([O-])=O)cc5F)c4c3)=C2	0,16
	CN(CC1)CCC1N2N=CC(c3cnc(NC=C4c5c(F)c(F)cc([N+]([O-])=O)c5)c4c3)=C2	0,23
	CN(CC1)CCC1N2N=CC(c3cnc(NC=C4c5cc(F)c(F)c([N+]([O-])=O)c5)c4c3)=C2	0,22
	CN(CC1)CCC1N2N=CC(c3cnc(NC=C4c5cc(F)cc([N+]([O-])=O)c5)c4c3)=C2	0,26
	CN(CC1)CCC1N2N=CC(c3cnc(NC=C4c5cc(F)c(C(F)(F)F)cc5[N+]([O-<br>])=O)c4c3)=C2	0,64
	CN(CC1)CCC1N2N=CC(c3cnc(NC=C4c5cc(F)c(C)cc5[N+]([O-])=O)c4c3)=C2	0,32
	CN(CC1)CCC1N2N=CC(c3cnc(NC=C4c5cc(F)c(OC)c(C(F)(F)F)c5)c4c3)=C2	0,25
	CN(CC1)CCC1N2N=CC(c3cnc(NC=C4c5c(C)c(Cl)c(C)cc5)c4c3)=C2	0,38
	CN(CC1)CCC1N2N=CC(c3cnc(NC=C4c5c(Cl)c(Cl)c(C)cc5)c4c3)=C2	0,25
	CN(CC1)CCC1N2N=CC(c3cnc(NC=C4c5c(F)c(Cl)c(F)cc5)c4c3)=C2	0,21

	CN(CC1)CCC1N2N=CC(c3cnc(NC=C4c5c(F)c(Cl)c(Cl)cc5)c4c3)=C2	0,18
	CN(CC1)CCC1N2N=CC(c3cnc(NC=C4c5cc(Cl)c(C)c(Cl)c5)c4c3)=C2	0,22
	CN(CC1)CCC1N2N=CC(c3cnc(NC=C4c5cc(Cl)c(F)c(Cl)c5)c4c3)=C2	0,39
	CN(CC1)CCC1N2N=CC(c3cnc(NC=C4c5cc(Cl)c(F)c(C)c5)c4c3)=C2	0,43
	CN(CC1)CCC1N2N=CC(c3cnc(NC=C4c5cc(Cl)c(Cl)c(C)c5)c4c3)=C2	0,14
	CN(CC1)CCC1N2N=CC(c3cnc(NC=C4c5cc(Cl)c(Cl)c(Cl)c5)c4c3)=C2	0,14
	CN(CC1)CCC1N2N=CC(c3cnc(NC=C4c5cc(Cl)c(Cl)cc5C)c4c3)=C2	0,53
	CN(CC1)CCC1N2N=CC(c3cnc(NC=C4c5cc(Cl)c(Cl)cc5F)c4c3)=C2	0,22
	CN(CC1)CCC1N2N=CC(c3cnc(NC=C4c5c(F)c(Cl)ccc5F)c4c3)=C2	0,36
	CN(CC1)CCC1N2N=CC(c3cnc(NC=C4c5c(F)c(Cl)cc(Cl)c5)c4c3)=C2	0,18
	CN(CC1)CCC1N2N=CC(c3cnc(NC=C4c5c(F)c(Cl)c(OC)cc5)c4c3)=C2	0,30
	CN(CC1)CCC1N2N=CC(c3cnc(NC=C4c5c(Cl)c(Cl)ccc5OC)c4c3)=C2	0,43
	CN(CC1)CCC1N2N=CC(c3cnc(NC=C4c5c(OC)c(Cl)c(C)cc5)c4c3)=C2	0,33
	CN(CC1)CCC1N2N=CC(c3cnc(NC=C4c5c(OC)c(Cl)ccc5F)c4c3)=C2	0,17
	CN(CC1)CCC1N2N=CC(c3cnc(NC=C4c5c(OC)c(Cl)c(Cl)cc5)c4c3)=C2	0,10
	CN(CC1)CCC1N2N=CC(c3cnc(NC=C4c5cc(Cl)c(OC)cc5OC)c4c3)=C2	0,26
	CN(CC1)CCC1N2N=CC(c3cnc(NC=C4c5cc(Cl)c(OC)cc5F)c4c3)=C2	0,22
	CN(CC1)CCC1N2N=CC(c3cnc(NC=C4c5cc(Cl)c(OC)c(F)c5)c4c3)=C2	0,53
	CN(CC1)CCC1N2N=CC(c3cnc(NC=C4c5cc(Cl)c(OC)c(Cl)c5)c4c3)=C2	0,22
	CN(CC1)CCC1N2N=CC(c3cnc(NC=C4c5cc(Cl)cc([N+]([O-])=O)c5C)c4c3)=C2	0,83
	CN(CC1)CCC1N2N=CC(c3cnc(NC=C4c5cc(Cl)c(Cl)c(OC)c5)c4c3)=C2	0,21
	CN(CC1)CCC1N2N=CC(c3cnc(NC=C4c5cc(Cl)c(Cl)cc5[N+]([O-])=O)c4c3)=C2	0,33
	CN(CC1)CCC1N2N=CC(c3cnc(NC=C4c5c(F)c(C)c(Cl)cc5)c4c3)=C2	0,12
	CN(CC1)CCC1N2N=CC(c3cnc(NC=C4c5c(F)c(C)ccc5OC)c4c3)=C2	0,19
	CN(CC1)CCC1N2N=CC(c3cnc(NC=C4c5c(F)c(C)ccc5Cl)c4c3)=C2	0,21
	CN(CC1)CCC1N2N=CC(c3cnc(NC=C4c5c(Cl)c(C)ccc5F)c4c3)=C2	0,18
	CN(CC1)CCC1N2N=CC(c3cnc(NC=C4c5c(Cl)c(C)c(Cl)cc5)c4c3)=C2	0,16
	CN(CC1)CCC1N2N=CC(c3cnc(NC=C4c5c(F)c(C(F)(F)F)c(F)cc5)c4c3)=C2	0,10
	CN(CC1)CCC1N2N=CC(c3cnc(NC=C4c5c(F)c(OC)cc(OC)c5F)c4c3)=C2	0,19
	CN(CC1)CCC1N2N=CC(c3cnc(NC=C4c5c(F)c(OC)c(F)cc5)c4c3)=C2	0,11
	CN(CC1)CCC1N2N=CC(c3cnc(NC=C4c5c(F)c(OC)ccc5F)c4c3)=C2	0,27
	CN(CC1)CCC1N2N=CC(c3cnc(NC=C4c5c(F)c(OC)ccc5Cl)c4c3)=C2	0,51
	CN(CC1)CCC1N2N=CC(c3cnc(NC=C4c5c(F)c(OC)ccc5[N+]([O-])=O)c4c3)=C2	0,70
	CN(CC1)CCC1N2N=CC(c3cnc(NC=C4c5c(Cl)c(OC)ccc5F)c4c3)=C2	0,45
	CN(CC1)CCC1N2N=CC(c3cnc(NC=C4c5c(Cl)c(OC)cc(OC)c5)c4c3)=C2	0,17
	CN(CC1)CCC1N2N=CC(c3cnc(NC=C4c5c(Cl)c(OC)c(Cl)cc5)c4c3)=C2	0,16
	CN(CC1)CCC1N2N=CC(c3cnc(NC=C4c5c(Cl)c(OC)cc(Cl)c5)c4c3)=C2	0,11
	CN(CC1)CCC1N2N=CC(c3cnc(NC=C4c5cc(OC)c(F)c(C)c5)c4c3)=C2	0,09

	CN(CC1)CCC1N2N=CC(c3cnc(NC=C4c5cc(OC)c(C)c(C)c5)c4c3)=C2	0,06
7	CN(CC1)CCC1N2N=CC(c3cnc(NC=C4c5cc(OC)c(C)cc5F)c4c3)=C2	0,04
	CN(CC1)CCC1N2N=CC(c3cnc(NC=C4c5cc(OC)c(C)c(OC)c5)c4c3)=C2	0,17
	CN(CC1)CCC1N2N=CC(c3cnc(NC=C4c5cc(OC)c(C)cc5OC)c4c3)=C2	0,07
	CN(CC1)CCC1N2N=CC(c3cnc(NC=C4c5c(OC)c(OC)c(F)cc5)c4c3)=C2	0,12
	CN(CC1)CCC1N2N=CC(c3cnc(NC=C4c5cc([N+]([O-])=O)c(C)cc5C)c4c3)=C2	0,36
	CN(CC1)CCC1N2N=CC(c3cnc(NC=C4c5c(F)c([N+]([O-])=O)ccc5F)c4c3)=C2	0,31
	CN(CC1)CCC1N2N=CC(c3cnc(NC=C4c5cc([N+]([O-])=O)cc(OC)c5)c4c3)=C2	0,12
	CN(CC1)CCC1N2N=CC(c3cnc(NC=C4c5cc([N+]([O-<br>])=O)cc(C(F)(F)F)c5F)c4c3)=C2	0,23
	CN(CC1)CCC1N2N=CC(c3cnc(NC=C4c5cc([N+]([O-])=O)c(Cl)cc5F)c4c3)=C2	0,18
	CN(CC1)CCC1N2N=CC(c3cnc(NC=C4c5c(Cl)c([N+]([O-])=O)ccc5Cl)c4c3)=C2	0,08

Table S3. ^{1}H-NMR spectral data (δ, DMSO-δ$_6$ or $CDCl_3$) of compounds **1**, **2**, **5** – **14**, **19** – **22**, **24** – **31**.

Compd.	2-H	4-H	6-H	N1-H	Other signals
**1**[1]	7.96	8.41	8.56	11.99	8.38; 8.00; 7.59 – 7.47; 7.07; 4.14 – 4.10; 2.87; 2.21; 2.31; 2.08 – 1.98.
**2**[2]	7.90	8.20	8.50	-	8.09; 7.47 – 7.42; 7.34 – 7.30; 7.26 – 7.22; 7.11 – 7.06; 2.39.
**5**[2]	7.83	8.32	8.71	11.73	8.28; 7.51; 7.40; 7.27; 7.21; 6.88; 4.36 – 4.28; 3.89; 3.15 – 3.11; 2.55 – 2.45; 2.31 – 2.20.
**6**[1]	8.07	8.36	8.56	12.13	8.36; 7.99; 7.78; 7.44; 4.13; 3.32; 2.87; 2.21; 2.03.
**7**[1]	7.72	8.17	8.55	11.93	8.31; 7.93; 7.15 – 7.11; 4.15 – 4.07; 3.87; 2.86; 2.20; 2.20; 2.08 – 1.96.
**8**[1]	7.96	8.41	8.56	12.02	8.36; 7.99; 7.65; 7.60 – 7.57; 7.51 – 7.45; 7.09 – 7.04; 4.24 – 4.16; 3.07 – 3.03; 2.63 – 2.56; 2.02 – 1.98; 1.88 – 1.78.
**9**[1]	7.97	8.34	8.55	11.91	8.38; 7.87; 7.38 – 7.36; 7.26; 6.86 – 6.83; 4.23 – 4.16; 3.89, 3.15 – 3.03; 2.63 – 2.60; 2.12 – 1.96.
**10**[1]	8.07	8.36	8.56	12.08	8.34; 7.99; 7.78; 7.44; 4.23 – 4.17; 3.05; 2.59; 1.99; 1.84 – 1.80.
**11**[1]	7.72	8.17	8.56	11.96	8.29; 7.92; 7.15 – 7.13; 4.22 – 4.14; 3.87; 3.04; 2.59; 2.20; 1.98; 1.86 – 1.76.
**12**[2]	7.89	8.21	8.48	-	8.09; 7.38; 7.29; 7.13 – 7.11; 7.07 – 7.06; 6.94 – 6.91; 3.87; 2.38.
**13**[2]	7.91	8.15	8.52	-	8.10; 7.42; 7.37; 7.31; 2.40.
**14**[2]	7.90	8.06	8.48	-	8.10; 7.30; 7.00; 6.85; 3.86; 2.39; 2.26.

**19**[2]	7.89	8.08	8.61	-	8.13; 7.79; 7.73; 7.49 – 7.43; 7.40 - 7.37; 7.32 – 7.29; 7.11 – 7.08; 4.37 – 4.28; 2.96 - 2.89; 2.38; 2.19; 2.03 – 1.93; 1.49.
**20**[2]	7.86	8.08	8.59	-	8.11; 7.77; 7.67; 7.41; 7.29; 7.18; 7.11; 6.93; 4.34 – 4.26; 3.88; 2.90; 2.37; 2.18 – 2.14; 2.01 – 1.90; 1.48.
**21**[2]	7.86	8.01	8.54	-	8.10; 7.79; 7.76; 7.44; 7.27 – 7.25; 4.34 – 4.22; 2.89; 2.33; 2.13; 2.04 – 1.90; 1.46.
**22**[2]	7.87	7.94	8.58	-	8.12; 7.75; 7.66; 7.29; 7.00; 6.89; 4.33 – 4.24; 3.86; 2.94 – 2.86; 2.37; 2.26; 2.17 – 2.13; 2.00 – 1.89; 1.47.
**24**[2]	7.88	8.06	8.61	-	8.12; 7.77; 7.70; 7.47 – 7.43; 7.39 - 7.37; 7.31 – 7.28; 7.11 – 7.06; 4.29 – 4.24; 3.29 – 3.24; 2.79; 2.38; 2.22 – 2.18; 1.98 – 1.88.
**25**[2]	7.86	8.09	8.59	-	8.12 – 8.09; 7.76; 7.69; 7.41; 7.28; 7.18; 7.12 – 7.11; 6.95 – 6.92; 4.28 – 4.22; 3.88; 3.28 – 3.23; 2.81 – 2.74; 2.37; 2.21 – 2.16; 1.98 – 1.87.
**26**[2]	7.88	8.01	8.62		8.12; 7.78; 7.71; 7.46; 7.37; 7.30; 4.31 – 4.23; 3.28 – 3.23; 2.78; 2.38; 2.21 – 2.17; 1.99 – 1.90.
**27**[2]	7.88	7.95	8.59		8.12; 7.74; 7.68; 7.29; 7.01; 6.90; 4.29 – 4.21; 3.86; 3.28 – 3.24; 2.78; 2.37; 2.27; 2.20 – 2.16; 1.95 – 1.91.
**28**[2]	7.89	8.08	8.62	-	8.13; 7.79; 7.73; 7.49 – 7.43; 7.40 – 7.36; 7.32 – 7.29; 7.11 – 7.06; 4.20 – 4.14; 3.00; 2.38; 2.34; 2.22 – 2.08.
**29**[2]	7.87	8.09	8.60	-	8.11; 7.77; 7.71; 7.41; 7.29; 7.19; 7.13; 6.94; 4.19 – 4.13; 3.88; 3.01 – 2.98; 2.37; 2.34; 2.22 – 2.07.
**30**[2]	7.88	8.01	8.61		8.12; 7.77; 7.70; 7.46; 7.37; 7.30; 4.22 – 4.13; 3.01 – 2.98; 2.38; 2.34; 2.22 – 2.10.
**31**[2]	7.88	7.94	8.58		8.12; 7.73; 7.66; 7.29; 7.01; 6.90; 4.17 – 4.12; 3.86; 2.99 – 2.96; 2.37; 2.33; 2.27; 2.19 – 2.07.

[1]: DMSO-d_6, [2]: $CDCl_3$

Table S4. ^{13}C-NMR spectral data (δ, DMSO-δ_6 or $CDCl_3$) of compounds **1**, **2**, **5** – **14**, **19** – **22**, **24** – **31**.

Compd.	C-2	C-3	C-3a	C-4	C-5	C-6	C-7a	Other signals
**1**[1]	125.2	113.1 (d, *J* = 3 Hz)	117.1	123.3	121.7	141.0	147.9	162.8 (d, *J = 242 Hz*); 137.6 (d, *J = 8 Hz*); 135.7; 130.7 (d, *J = 9 Hz*); 124.9; 122.2 (d, *J = 1 Hz*); 119.2; 112.6 (d, *J = 21 Hz*); 112.1 (d, *J = 21 Hz*); 58.3; 54.2 (2C); 45.8; 32.1 (2C).
**2**[2]	124.5	118.6 (d, J = 2 Hz)	122.9	131.1	115.8	146.1	145.8	163.3 (d, *J = 246 Hz*); 145.9; 135.0; 134.3 (d, *J = 8 Hz*); 131.0 (d, *J = 8 Hz*); 130.0 (2C); 128.4 (2C); 123.2 (d, *J = 3 Hz*); 115.0 (d, *J = 21 Hz*); 114.4 (d, *J = 22 Hz*); 21.8.
**5**[2]	124.7	111.6	118.6	123.4	122.2	141.6	148.6	160.2; 136.6; 135.9; 130.1; 123.8; 121.3; 119.8; 116.3; 113.1; 59.7; 55.5; 55.1 (2C); 46.0; 32.6 (2C).
**6**[1]	126.4	111.6	116.8	123.2	122.0	141.3	147.9	138.9; 135.8; 134.6 (2C); 125.1; 124.8; 124.4 (2C); 119.6; 58.3; 54.2 (2C); 45.8; 32.1 (2C).
**7**[1]	125.9 (d, *J* = 5 Hz)	108.5	118.0	123.6 (d, *J* = 4 Hz)	121.3	140.9	147.4	153.6 (d, *J = 1 Hz*); 152.8 (d, *J = 235 Hz*); 135.5; 125.1 (d, *J = 8 Hz*); 124.7; 119.8; 119.6 (d, J = 7 Hz); 117.6 (d, *J = 14 Hz*); 111.2 (d, *J = 5 Hz*); 59.3; 55.8; 54.2 (2C); 45.8; 32.1 (2C); 15.7.
**8**[1]	125.2	113.0 (d, *J* = 3 Hz)	117.1	123.3	121.8	141.0	147.9	162.8 (d, *J = 241 Hz*); 137.6 (d, *J = 8 Hz*); 135.6; 130.7 (d, *J = 9 Hz*); 124.7; 122.2 (d, *J = 2 Hz*); 119.7; 112.5 (d, *J = 22 Hz*); 112.1 (d, *J = 21 Hz*); 59.4; 45.2 (2C); 33.7 (2C).
**9**[1]	124.9	111.6	117.6	121.8	119.9	141.2	147.9	159.9; 136.6; 135.7; 130.2; 124.6; 123.6; 119.1; 114.5; 111.9; 59.4; 55.3; 45.1 (2C); 33.6 (2C).
**10**[1]	126.5	111.5	116.9	123.2	122.0	141.3	148.0	138.9; 135.7; 134.6 (2C); 124.9, 124.7, 124.4 (2C); 119.6; 59.4; 45.2 (2C); 33.7 (2C).
**11**[1]	125.8 (d, *J* = 4 Hz)	108.6	118.0	123.6 (d, *J* = 4 Hz)	121.4	141.0	147.4	153.6 (d, *J = 1 Hz*); 152.8 (d, *J = 235 Hz*); 135.4; 125.1 (d, *J = 8 Hz*); 124.5; 123.6 (d, *J = 4 Hz*); 119.7; 119.6 (d, J = 14 Hz); 117.6 (d, *J = 14 Hz*); 111.3 (d, *J = 5 Hz*); 59.3; 55.8; 45.2 (2C); 33.6 (2C); 15.7.
**12**[2]	124.2	119.6	123.2	131.1	115.6	145.8	145.9	160.3; 145.7; 135.1; 133.4; 130.4; 129.9 (2C); 128.3 (2C); 119.9; 113.4; 113.3; 55.5; 21.8.
**13**[2]	125.1	117.1	122.4	130.7	115.9	146.4	145.7	146.0; 135.9 (2C); 135.2; 134.8; 130.0 (2C); 128.4 (2C); 128.0; 125.8 (2C); 21.8.
**14**[2]	125.7 (d, *J* = 3 Hz)	114.3	123.6	131.9 (d, *J* = 5 Hz)	115.6	145.8	145.5	154.3 (d, *J = 2 Hz*); 153.7 (d, *J = 239 Hz*); 145.8; 135.0; 129.9 (2C); 128.9 (d, *J = 8 Hz*); 128.4 (2C); 118.4 (d, *J = 24 Hz*); 116.6 (d, *J = 16 Hz*); 110.9 (d, *J = 4 Hz*); 56.2; 21.8; 16.3.

**19**[2]	124.9	119.1 (d, *J* = 3 Hz)	121.4	125.1	124.0	143.3	146.3	163.2 (d, *J* = 246 Hz); 154.6; 145.5; 136.6; 135.2; 134.9 (d, *J* = 8 Hz); 130.8 (d, *J* = 7 Hz); 129.8 (2C); 128.2 (2C); 123.8; 123.2 (d, *J* = 2 Hz); 119.7; 114.6 (d, *J* = 21 Hz); 114.4 (d, *J* = 22 Hz); 80.0; 59.7; 32.5 (CH_2); 28.5 (5C); 24.9; 21.7.
**20**[2]	124.8	119.9	121.8	125.5	124.0	143.2	146.5	160.3; 154.7; 145.4; 136.7; 135.5; 134.1; 130.3; 129.8 (2C); 128.2 (2C); 123.5; 120.3; 120.1; 113.5; 113.1; 80.1; 59.8; 55.6; 32.6 (2C); 28.6 (5C); 21.8.
**21**[2]	124.9	117.3	120.5	127.2	124.2	143.2	145.7	154.2; 145.3; 136.3; 135.6; 135.3 (2C); 134.8; 129.5 (2C); 128.0 (2C); 125.5 (2C); 124.5; 124.1; 119.1; 79.5; 59.3; 32.1 (2C); 28.2 (3C); 24.6 (2C); 21.3.
**22**[2]	124.9 (d, *J* = 3 Hz)	114.9	122.2	126.1 (d, *J* = 5 Hz)	124.7	143.2	146.0	154.7; 154.2 (d, *J* = 2 Hz); 153.8 (d, *J* = 239 Hz); 145.5; 136.7; 135.4; 129.8 (2C); 128.6 (d, *J* = 8 Hz); 128.3 (2C); 123.9; 120.0; 118.3 (d, J = 24 Hz); 117.2 (d, *J* = 16 Hz); 111.1 (d, *J* = 4 Hz); 80.1; 59.7; 56.2; 32.5 (2C); 28.5 (5C); 21.8; 16.2 (d, *J* = 1 Hz).
**24**[2]	125.6	119.7 (d, J = 2 Hz)	121.9	125.7	126.5	143.6	146.9	164.0 (d, *J* = 243 Hz); 145.6; 136.9; 136.2; 136.0 (d, *J* = 8 Hz); 131.7 (d, *J* = 9 Hz); 130.6 (2C); 129.0 (2C); 125.0; 124.3 (d, *J* = 2 Hz); 119.8; 115.0 (d, *J* = 21 Hz); 114.9 (d, *J* = 21 Hz); 60.2; 45.6 (2C) 33.9 (2C); 21.5.
**25**[2]	125.0	119.8	121.8	125.5	123.8	143.3	146.4	160.3; 145.4; 136.5; 135.5; 134.1; 130.3; 129.8 (2C); 128.2 (2C); 123.5; 120.3; 120.1; 113.5; 113.1; 60.1; 55.5; 45.8 (2C; 34.1 (2C); 21.8.
**26**[2]	124.9	117.8	121.0	125.3	124.3	143.8	146.2	145.7; 136.5; 135.9; 135.8 (2C); 135.2; 129.9 (2C); 128.4 (2C); 127.8; 125.9 (2C);<br>123.9; 119.5; 60.2; 45.8 (2C); 34.1 (2C); 21.8.
**27**[2]	124.9 (d, *J* = 3 Hz)	115.0	122.2	126.1 (d, *J* = 4 Hz)	124.8	143.2	146.0	154.2 (d, *J* = 2 Hz); 153.8 (d, *J* = 239 Hz); 145.4; 136.4; 135.4; 129.8 (2C); 128.6 (d, *J* = 8 Hz); 128.3 (2C); 123.7; 119.8; 118.4 (d, J = 24 Hz); 117.2 (d, *J* = 16 Hz); 111.2 (d, *J* = 5 Hz); 60.0; 56.2; 45.8 (2C); 32.8 (2C); 21.8; 16.3.
**28**[2]	125.0	119.1 (d, J = 3 Hz)	121.4	125.1	123.7	143.3	146.2	163.2 (d, *J* = 245 Hz); 145.4; 136.3; 135.2; 134.9 (d, *J* = 8 Hz); 130.8 (d, *J* = 9 Hz); 129.8 (2C); 128.2 (2C); 123.6; 123.2 (d, *J* = 3Hz); 119.6; 114.6 (d, *J* = 21 Hz); 114.3 (d, *J* = 22 Hz); 59.3; 54.7 (2C); 46.0; 32.6 (2C); 21.7.
**29**[2]	124.9	119.8	121.8	125.4	123.7	143.2	146.4	160.2; 145.3; 136.4; 135.4; 134.0; 130.2; 129.8 (2C); 128.2 (2C); 123.4; 120.3; 120.0; 113.4; 113.1; 59.3; 55.4; 54.7 (2C); 46.1;

								32.7 (2C); 21.7.
**30**[2]	124.9	117.8	121.0	125.9	124.3	143.8	146.2	145.7; 136.5; 135.9; 135.8 (2C); 135.2; 129.9 (2C); 128.4 (2C); 127.8; 125.9 (2C); 124.0; 119.5; 59.5; 54.8 (2C); 46.2; 32.8 (2C); 21.8.
**31**[2]	124.9 (d, *J* = 3 Hz)	115.0	122.2	126.1 (d, *J* = 5 Hz)	124.8	143.2	146.0	154.2 (d, *J = 1 Hz*); 153.8 (d, *J = 239 Hz*); 145.4; 136.4; 135.4; 129.9 (2C); 128.6 (d, *J = 8 Hz*); 128.3 (2C); 123.6; 119.9; 118.4 (d, J = 24 Hz); 117.3 (d, J = 17 Hz); 111.2 (d, *J = 5 Hz*); 59.4; 56.2; 54.8 (2C); 46.2; 32.8 (2C); 21.8; 16.3.

[1]: DMSO-d_6, [2]: $CDCl_3$

Figures S1-S16: ^{1}H and ^{13}C NMR spectra of compounds **1**, **5 – 11.**

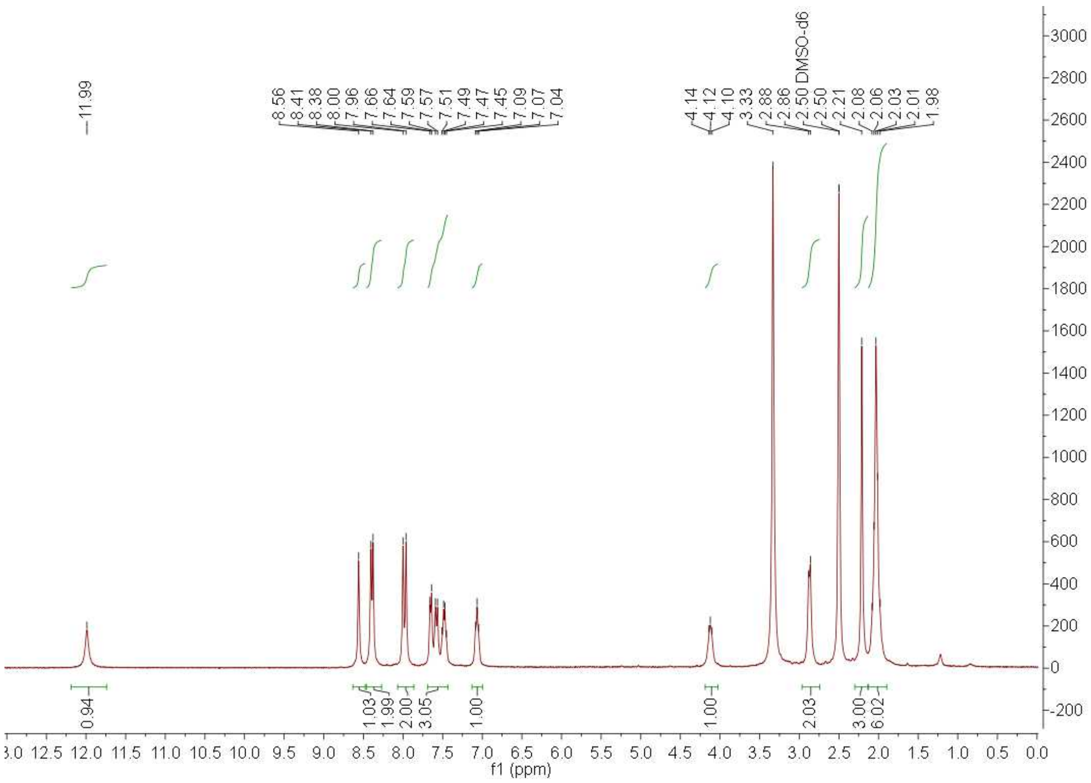

Figure S1. ^{1}H-NMR spectrum of **1** (400 MHz, DMSO-d_6).

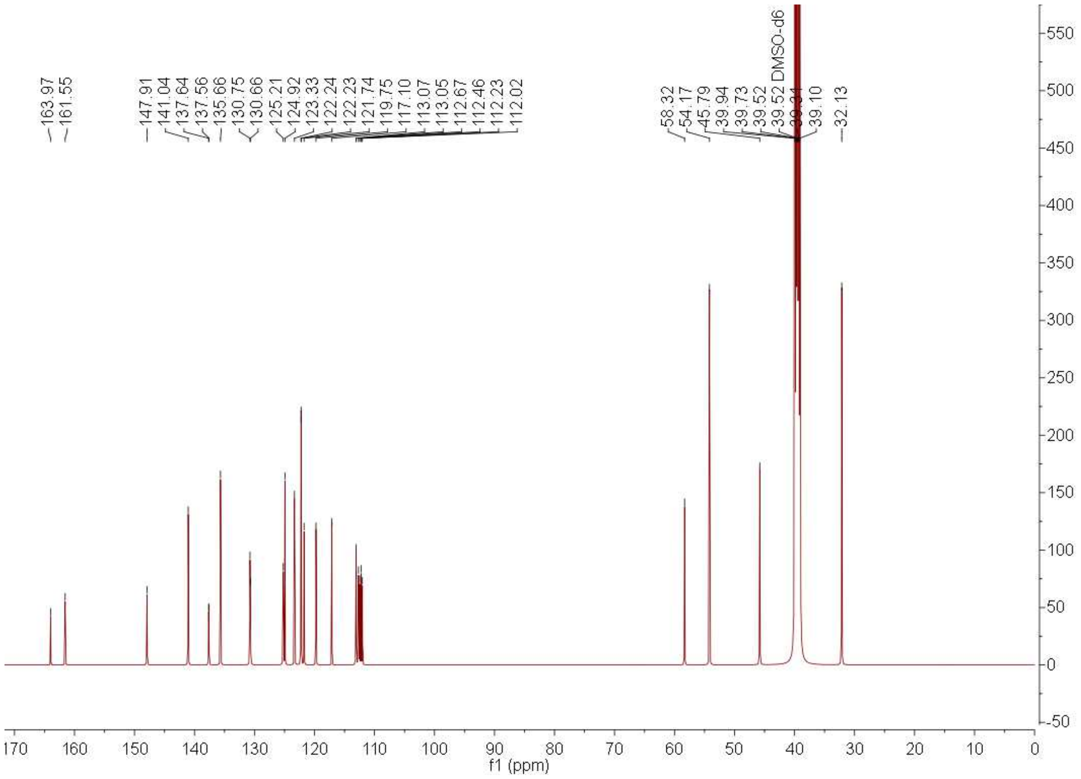

Figure S2. ^{13}C-NMR spectrum of **1** (100 MHz, DMSO-d_6).

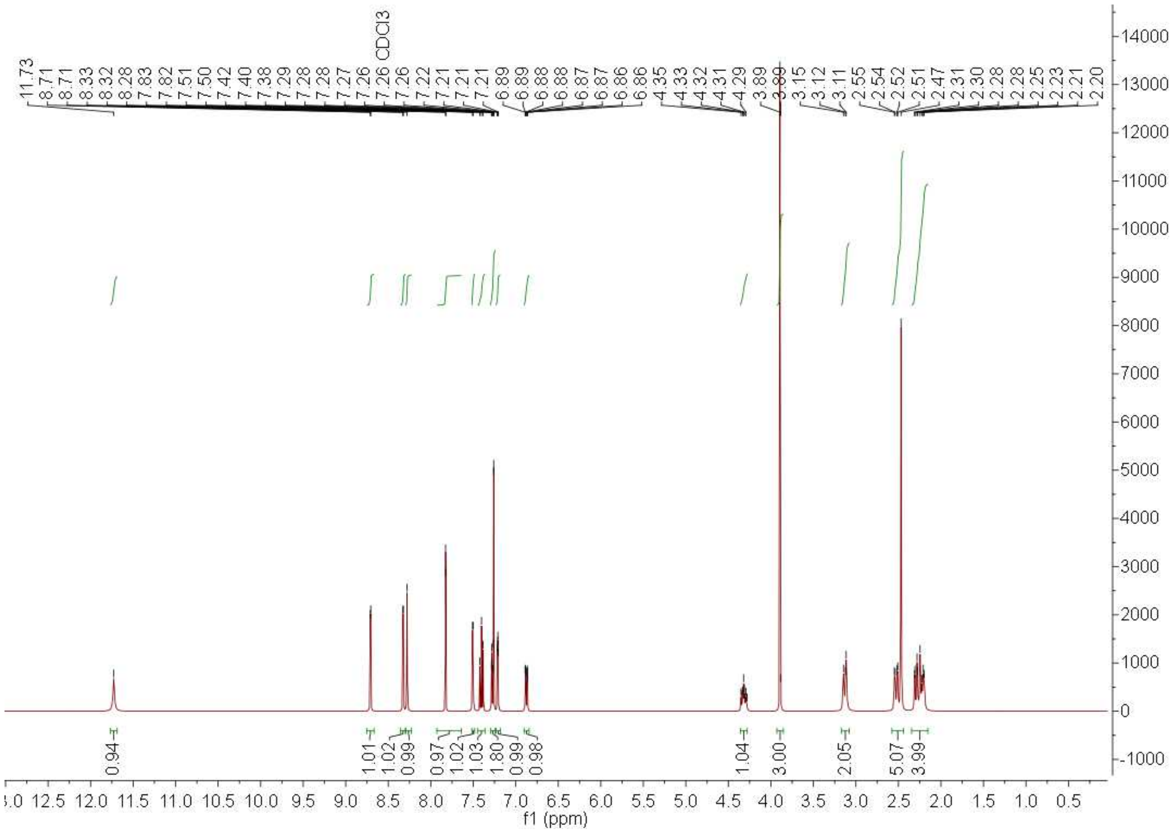

Figure S3. ^{1}H-NMR spectrum of **5** (400 MHz, $CDCl_3$).

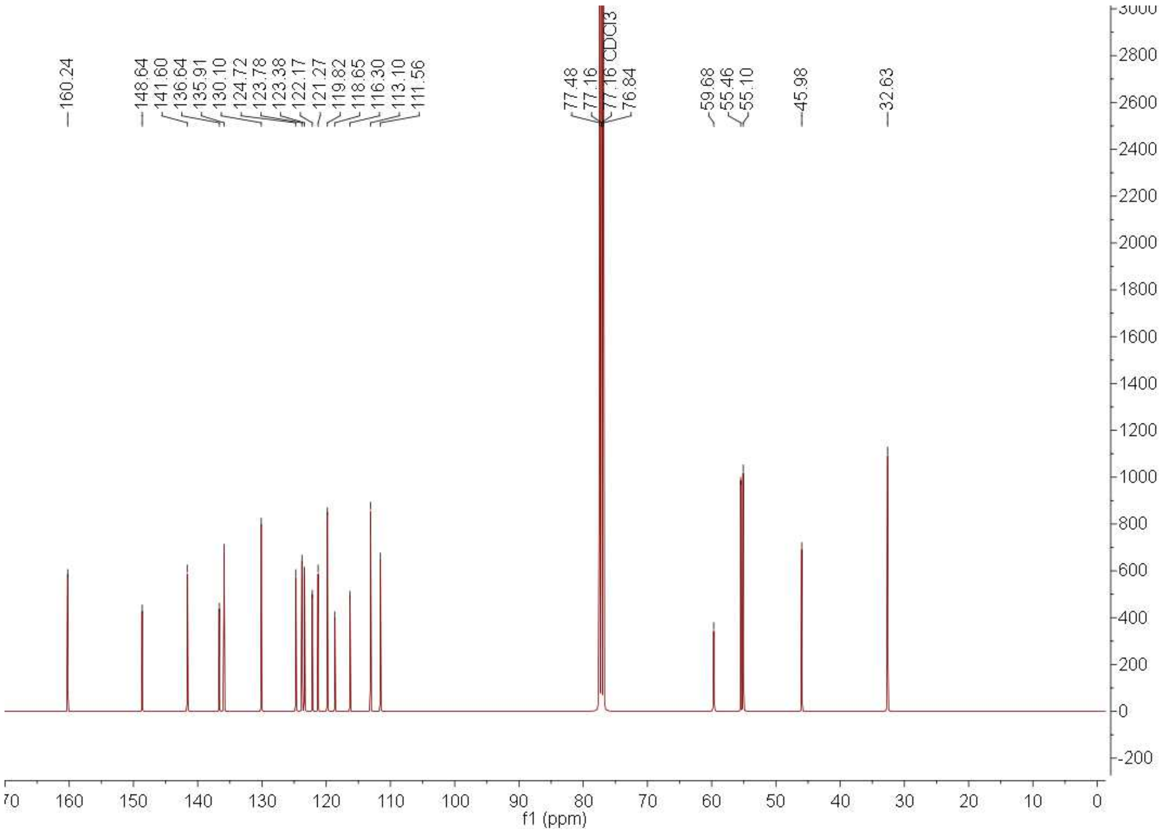

Figure S4. ^{13}C-NMR spectrum of **5** (100 MHz, $CDCl_3$).

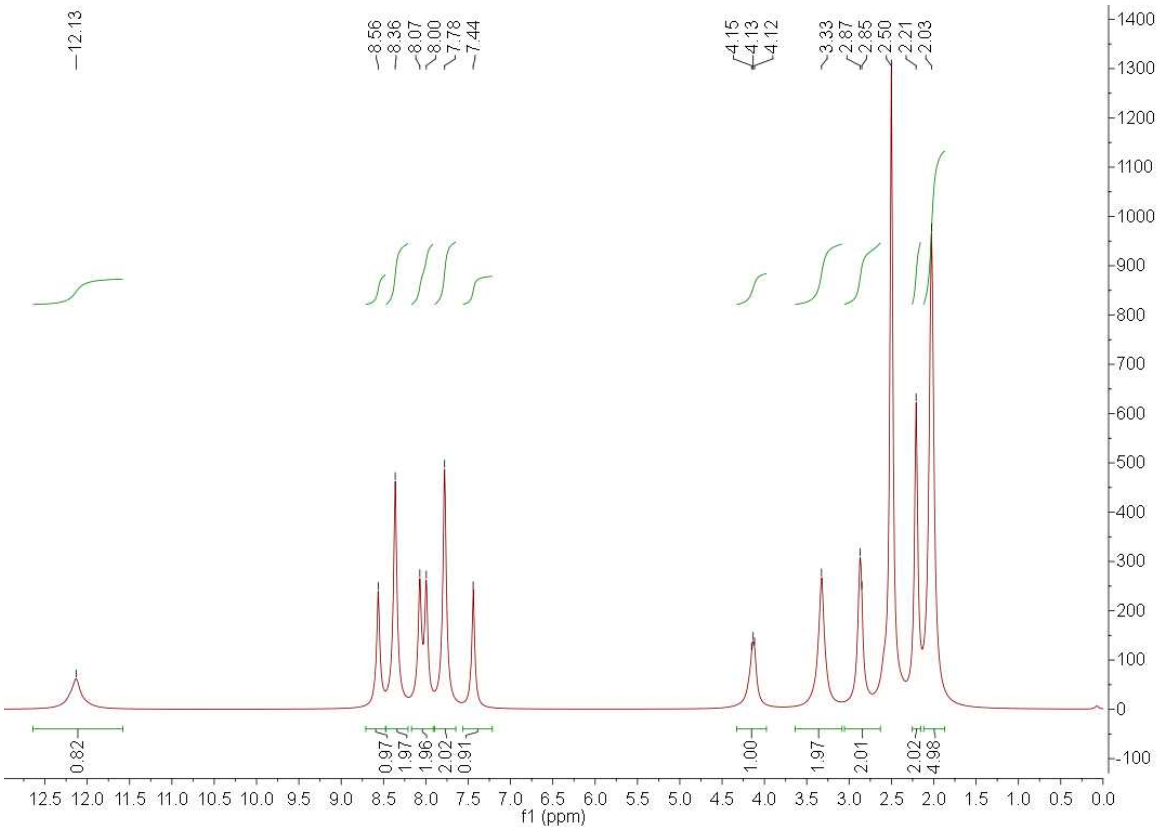

Figure S5. ¹H-NMR spectrum of **6** (400 MHz, DMSO-d_6).

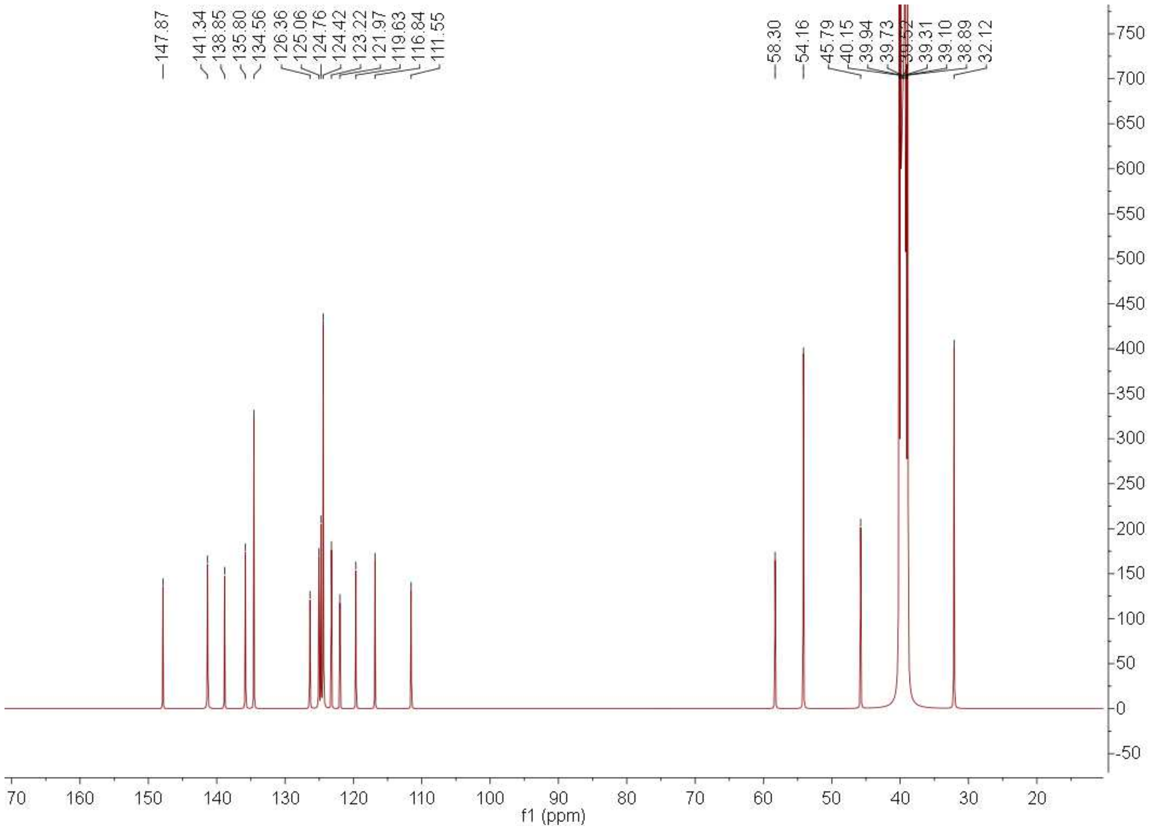

Figure S6. ¹³C-NMR spectrum of **6** (100 MHz, DMSO-d_6).

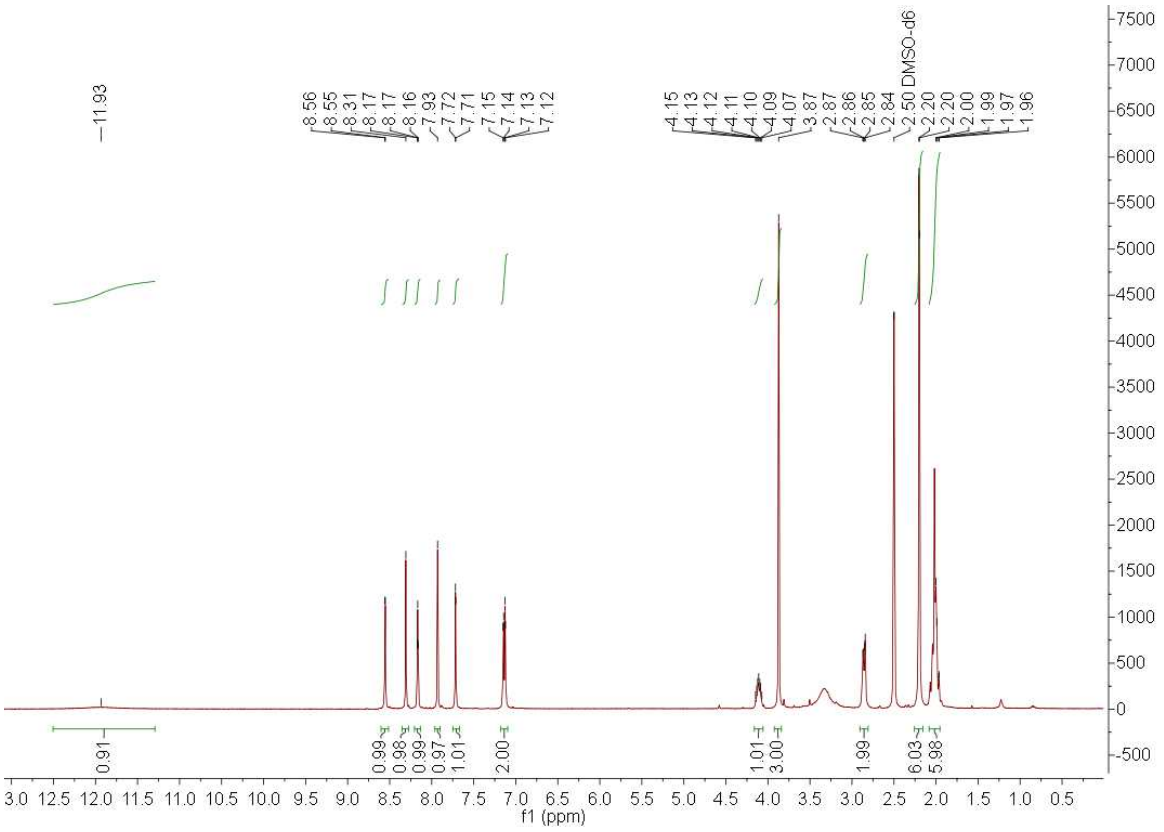

Figure S7. ^{1}H-NMR spectrum of **7** (400 MHz, DMSO-d$_6$).

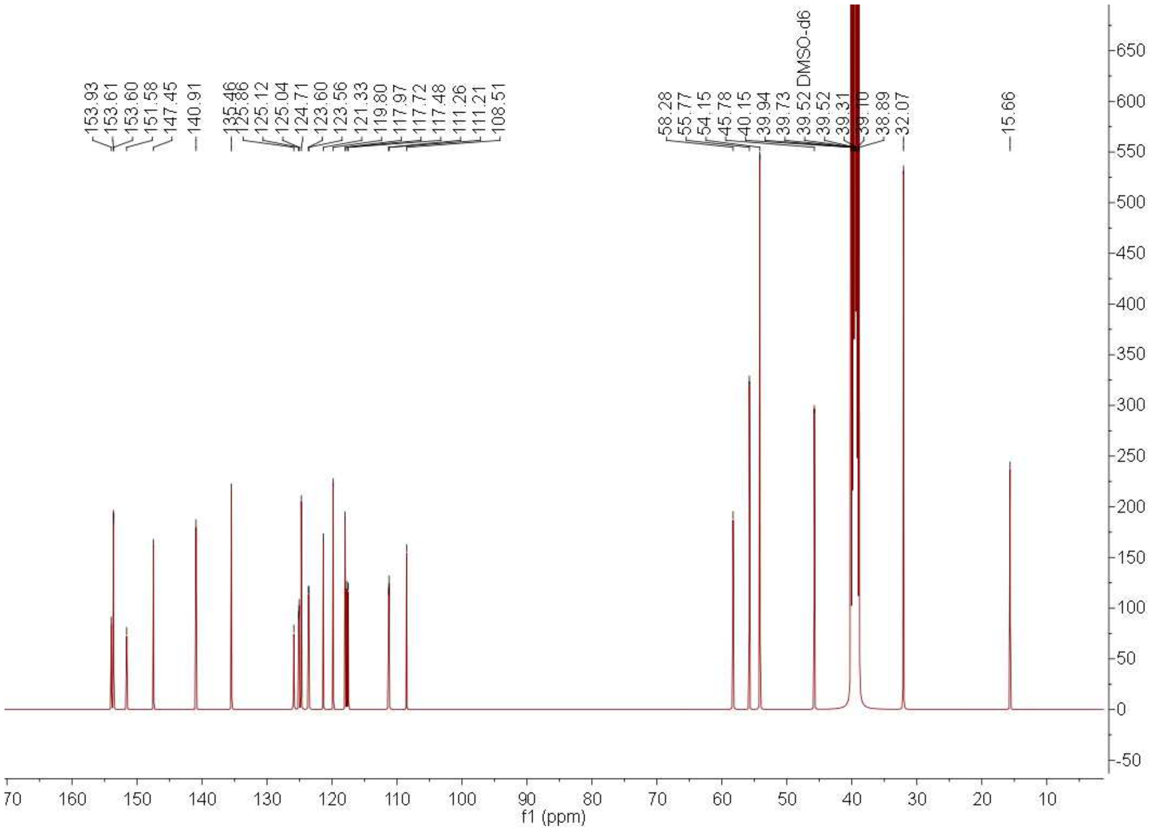

Figure S8. ^{13}C-NMR spectrum of **7** (100 MHz, DMSO-d$_6$).

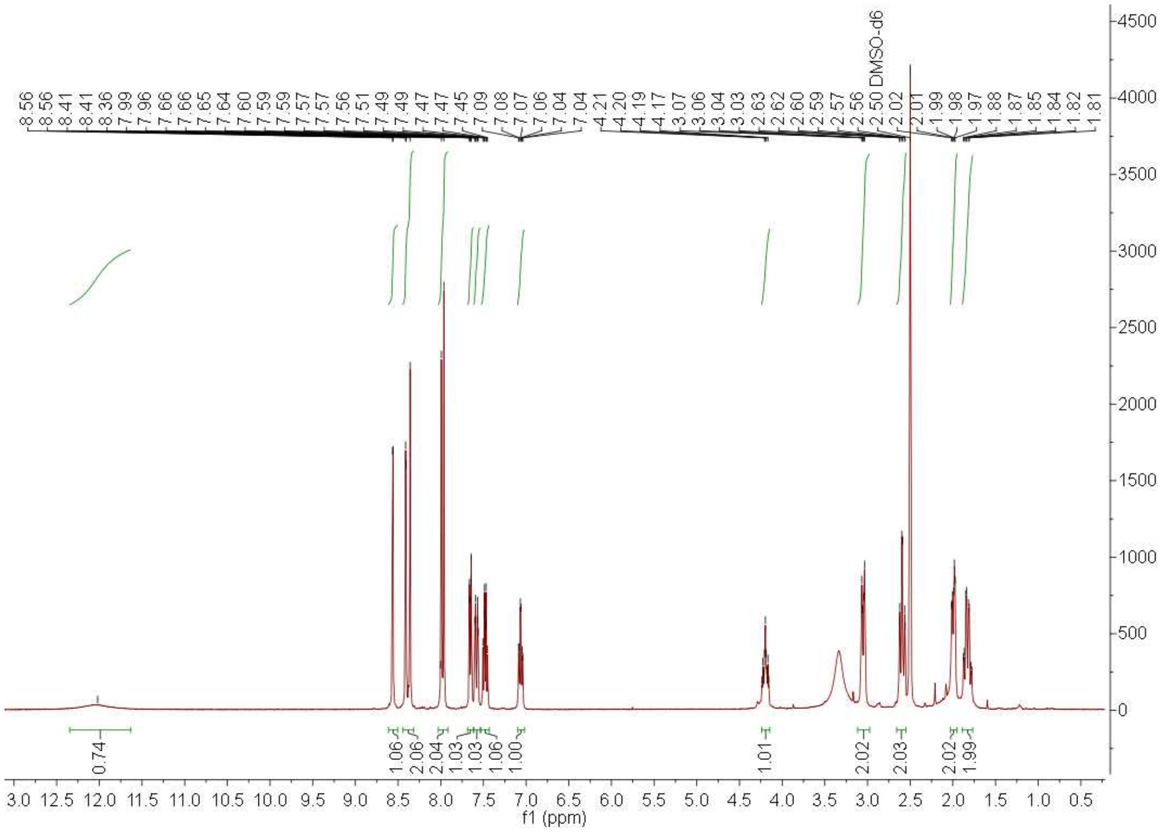

Figure S9. ^{1}H-NMR spectrum of **8** (400 MHz, DMSO-d_6).

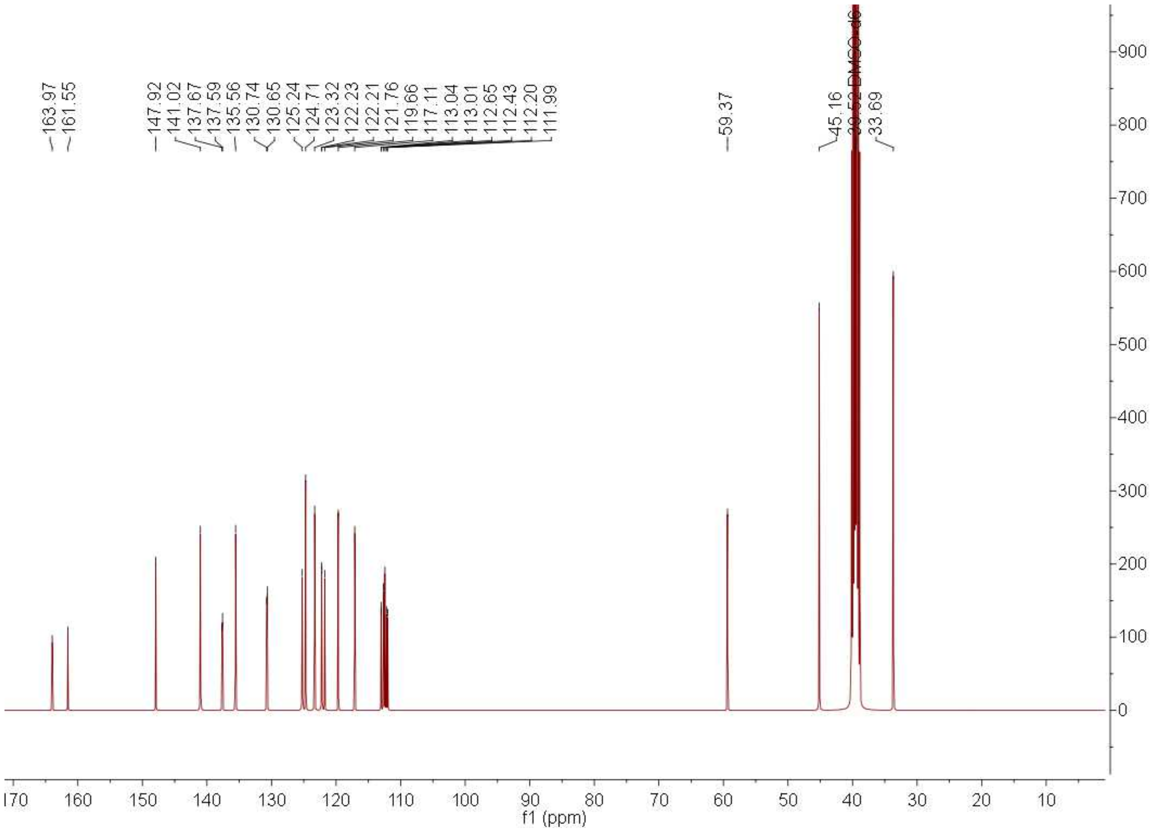

Figure S10. ^{13}C-NMR spectrum of **8** (100 MHz, DMSO-d_6).

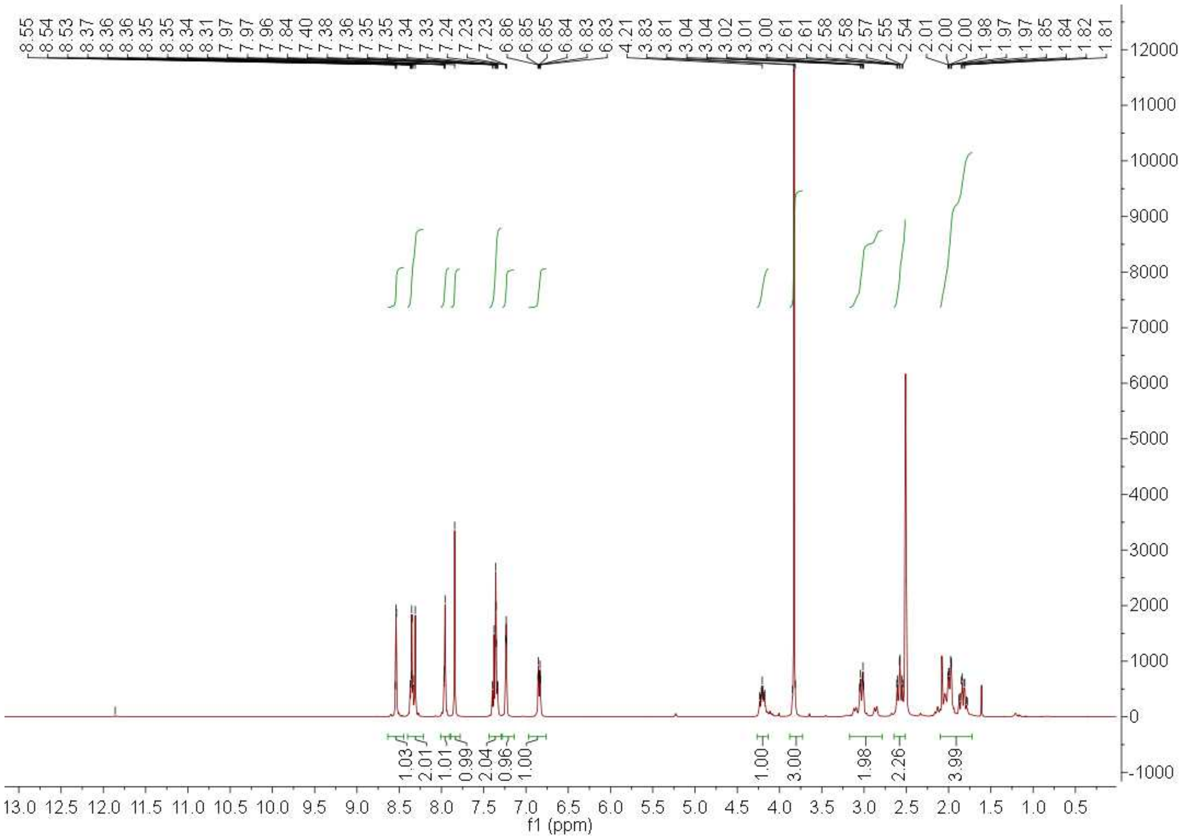

Figure S11. ^{1}H-NMR spectrum of **9** (400 MHz, DMSO-d$_6$).

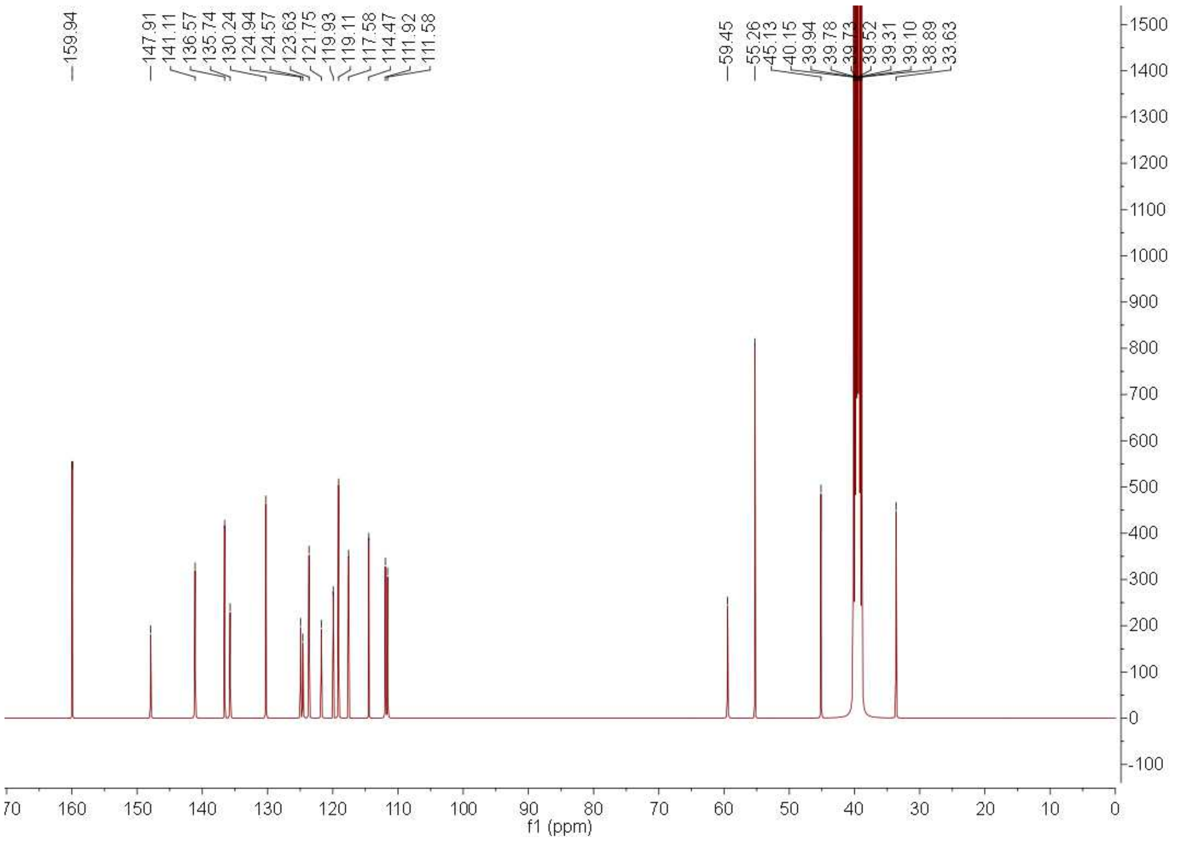

Figure S12. ^{13}C-NMR spectrum of **9** (100 MHz, DMSO-d$_6$).

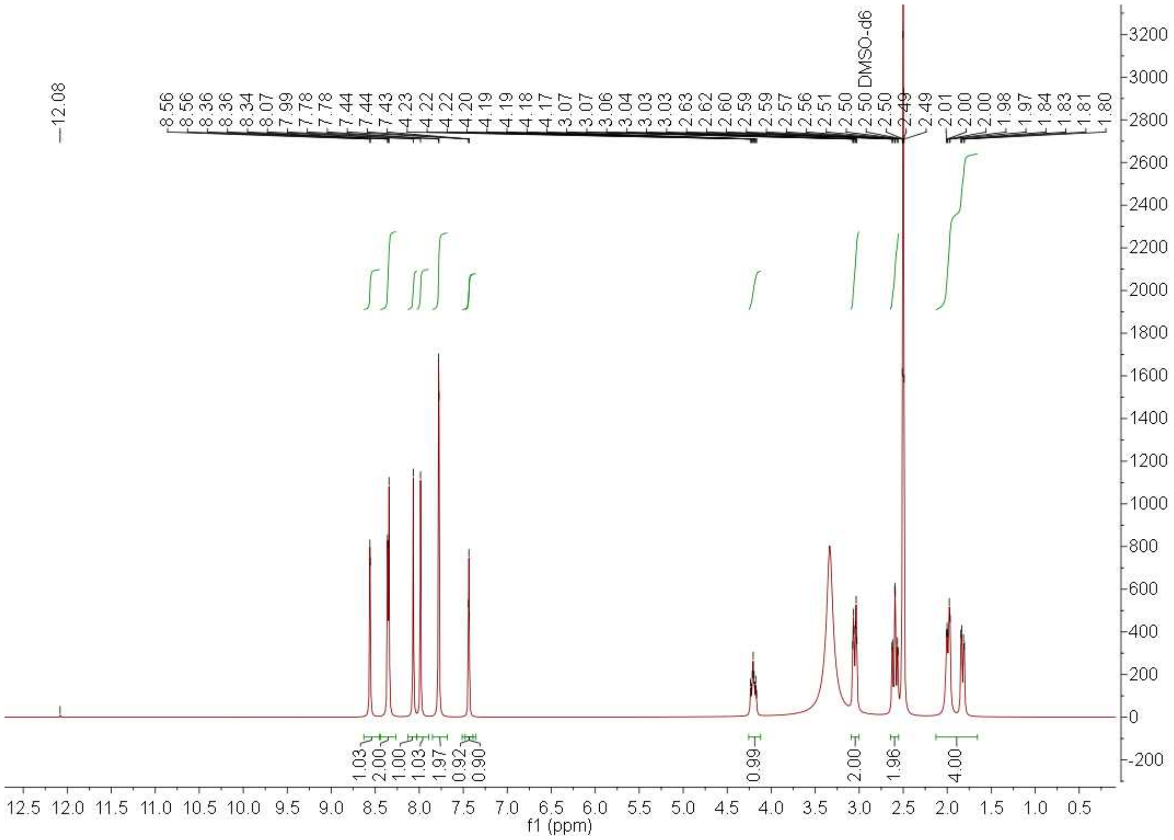

Figure S13. ^{1}H-NMR spectrum of **10** (400 MHz, DMSO-d_6).

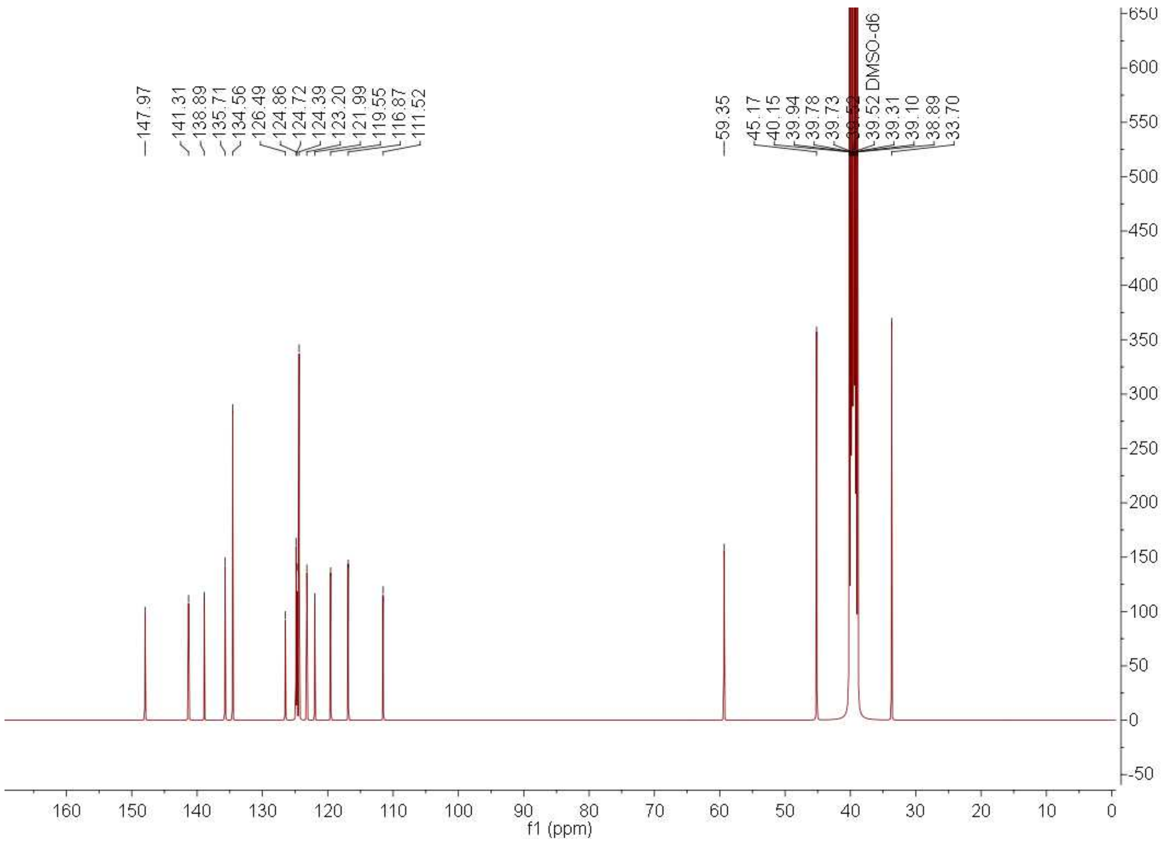

Figure S14. ^{13}C-NMR spectrum of **10** (100 MHz, DMSO-d_6).

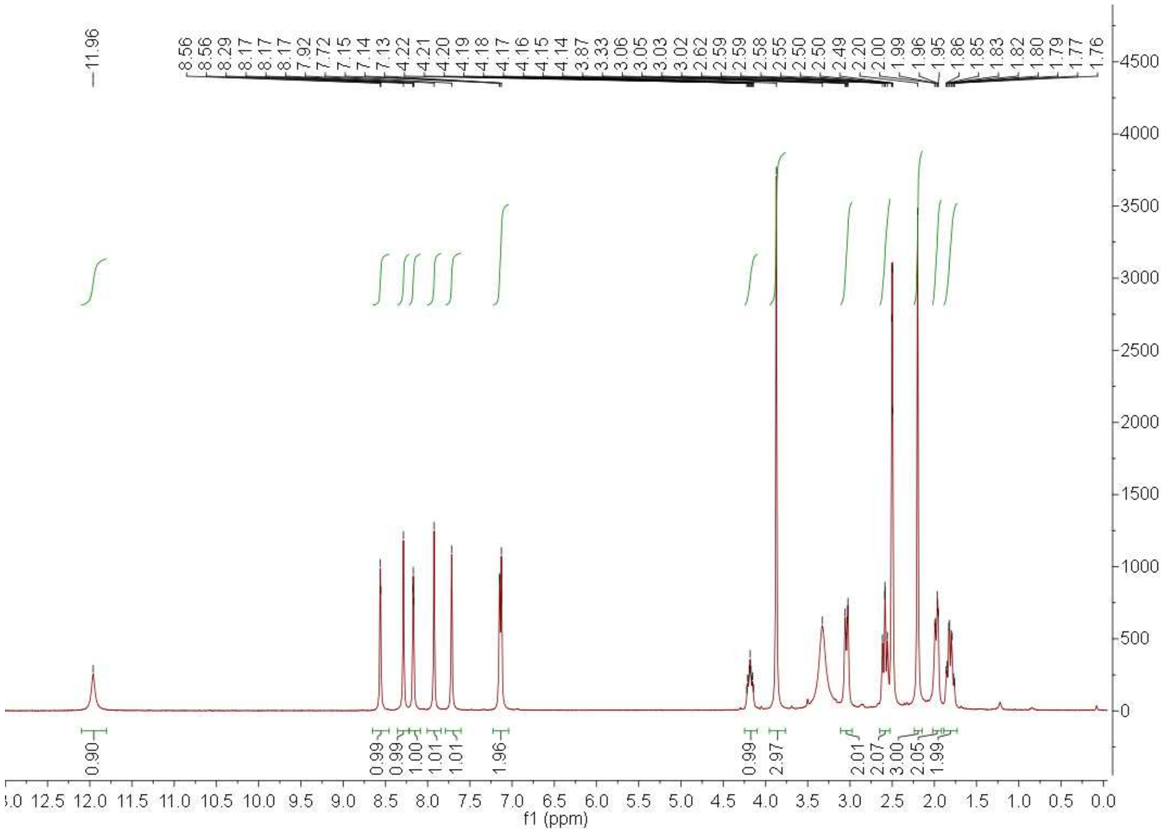

Figure S15. ^{1}H-NMR spectrum of **11** (400 MHz, DMSO-d$_6$).

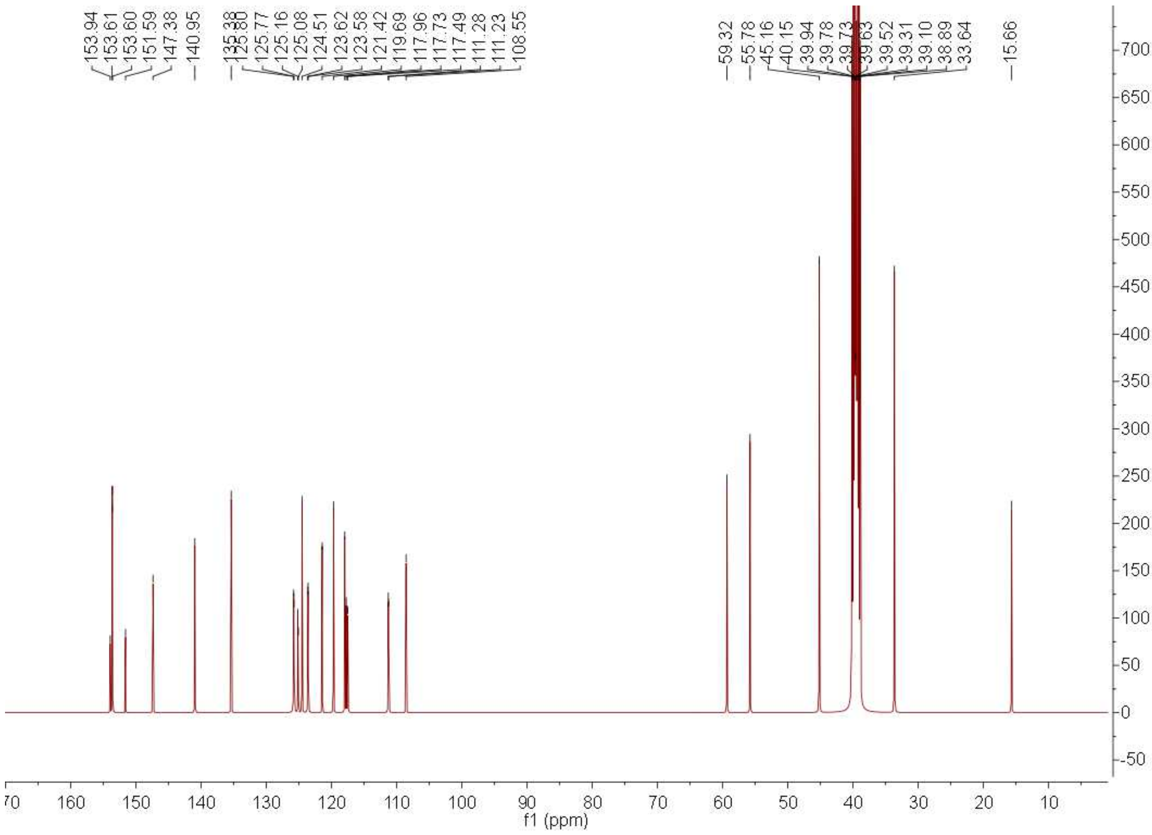

Figure S16. ^{13}C-NMR spectrum of **11** (100 MHz, DMSO-d$_6$).

Elemental Analysis Data of compounds **1, 2, 5 – 14, 19 – 22, 24 – 31**

3-(3-fluorophenyl)-5-(1-(1-methylpiperidin-4-yl)-1H-pyrazol-4-yl)-1H-pyrrolo[2,3-b]pyridine (1).

Anal. ($C_{22}H_{22}FN_5$, 375.44) % found. (% calcd) C: 70.52 (70.38); H: 5.76 (5.91); N: 18.31 (18.65).

5-bromo-3-(3-fluorophenyl)-1-tosyl-1H-pyrrolo[2,3-b]pyridine (2).

Anal. ($C_{20}H_{14}BrFN_2O_2S$, 445.30) % found. (% calcd) C: 53.78 (53.94); H: 3.44 (3.17); N: 6.58 (6.29); S: 7.18 (7.20).

3-(3-methoxyphenyl)-5-(1-(1-methylpiperidin-4-yl)-1H-pyrazol-4-yl)-1H-pyrrolo[2,3-b]pyridine (5).

Anal. ($C_{23}H_{25}N_5O$, 387.48) % found. (% calcd) C: 71.01 (71.29); H: 5.76 (6.66); N: 18.01 (18.07).

3-(3,5-dichlorophenyl)-5-(1-(1-methylpiperidin-4-yl)-1H-pyrazol-4-yl)-1H-pyrrolo[2,3-b]pyridine (6)

Anal. ($C_{22}H_{21}Cl_2N_5$, 426.34) % found. (% calcd) C: 62.16 (61.98); H: 5.16 (4.96); N: 16.13 (16.43).

3-(2-fluoro-5-methoxy-4-methylphenyl)-5-(1-(1-methylpiperidin-4-yl)-1H-pyrazol-4-yl)-1H-pyrrolo[2,3-b]pyridine (7).

Anal. ($C_{24}H_{26}FN_5O$, 419.49) % found. (% calcd) C: 68.80 (68.72); H: 6.52 (6.25); N: 16.72 (16.69).

3-(3-fluorophenyl)-5-(1-(piperidin-4-yl)-1H-pyrazol-4-yl)-1H-pyrrolo[2,3-b]pyridine (8).

Anal. ($C_{21}H_{20}FN_5$, 361.42) % found. (% calcd) C: 69.55 (69.79); H: 5.66 (5.58); N: 19.52 (19.38).

3-(3-methoxyphenyl)-5-(1-(piperidin-4-yl)-1H-pyrazol-4-yl)-1H-pyrrolo[2,3-b]pyridine (9).

Anal. ($C_{22}H_{23}N_5O$, 373.45) % found. (% calcd) C: 70.99 (70.76); H: 6.45 (6.21); N: 18.52 (18.75).

3-(3,5-dichlorophenyl)-5-(1-(piperidin-4-yl)-1H-pyrazol-4-yl)-1H-pyrrolo[2,3-b]pyridine (10).

Anal. ($C_{21}H_{19}Cl_2N_5$, 412.32) % found. (% calcd) C: 60.84 (61.17); H: 4.83 (4.64); N: 16.60 (16.99).

3-(2-fluoro-5-methoxy-4-methylphenyl)-5-(1-(piperidin-4-yl)-1H-pyrazol-4-yl)-1H-pyrrolo[2,3-b]pyridine (11). Anal. ($C_{23}H_{24}FN_5O$, 405.47) % found. (% calcd) C: 67.98 (68.13); H: 6.00 (5.97); N: 16.92 (17.27).

5-bromo-3-(3-methoxyphenyl)-1-tosyl-1H-pyrrolo[2,3-b]pyridine (12).

Anal. ($C_{21}H_{17}BrN_2O_3S$, 457.34) % found. (% calcd) C: 55.56 (55.15); H: 3.96 (3.75); N: 6.19 (6.13); S: 6.93 (7.01).

5-bromo-3-(3,5-dichlorophenyl)-1-tosyl-1H-pyrrolo[2,3-b]pyridine (13).

Anal. ($C_{20}H_{13}BrCl_2N_2O_2S$, 496.2) % found. (% calcd) C: 48.72 (48.41); H: 2.90 (2.64); N: 5.79 (5.65); S: 6.41 (6.46).

5-bromo-3-(2-fluoro-5-methoxy-4-methylphenyl)-1-tosyl-1H-pyrrolo[2,3-b]pyridine (14).

Anal. ($C_{22}H_{18}BrFN_2O_3S$, 489.36) % found. (% calcd) C: 53.65 (54.00); H: 3.92 (3.71); N: 5.92 (5.72); S: 6.56 (6.55).

Tert-butyl 4-(4-(3-(3-fluorophenyl)-1-tosyl-1H-pyrrolo[2,3-b]pyridin-5-yl)-1H-pyrazol-1-yl)piperidine-1-carboxylate (19).

Anal. ($C_{33}H_{34}FN_5O_4S$, 615.72) % found. (% calcd) C: 64.48 (64.37); H: 5.65 (5.57); N: 11.24 (11.37); S: 5.24 (5.21).

Tert-butyl 4-(4-(3-(3-methoxyphenyl)-1-tosyl-1H-pyrrolo[2,3-b]pyridin-5-yl)-1H-pyrazol-1-yl)piperidine-1-carboxylate (20).

Anal. ($C_{34}H_{37}N_5O_5S$, 627.75) % found. (% calcd) C: 65.31 (65.05); H: 6.05 (5.94); N: 11.11 (11.16); S: 5.06 (5.11).

Tert-butyl 4-(4-(3-(3,5-dichlorophenyl)-1-tosyl-1H-pyrrolo[2,3-b]pyridin-5-yl)-1H-pyrazol-1-yl)piperidine-1-carboxylate (21).

Anal. ($C_{33}H_{33}Cl_2N_5O_4S$, 666.62) % found. (% calcd) C: 59.59 (59.46); H: 5.23 (4.99); N: 10.15 (10.51); S: 4.40 (4.81).

Tert-butyl 4-(4-(3-(2-fluoro-5-methoxy-4-methylphenyl)-1-tosyl-1H-pyrrolo[2,3-b]pyridin-5-yl)-1H-pyrazol-1-yl)piperidine-1-carboxylate (22).

Anal. ($C_{35}H_{38}FN_5O_5S$, 659.77) % found. (% calcd) C: 63.51 (63.72); H: 5.94 (5.81); N: 10.66 (10.61); S: 4.85 (4.86).

3-(3-fluorophenyl)-5-(1-(piperidin-4-yl)-1H-pyrazol-4-yl)-1-tosyl-1H-pyrrolo[2,3-b] pyridine (24).

Anal. ($C_{28}H_{26}FN_5O_2S$, 515.6) % found. (% calcd) C: 64.95 (65.22); H: 5.25 (5.08); N: 13.37 (13.58); S: 6.11 (6.22).

3-(3-methoxyphenyl)-5-(1-(piperidin-4-yl)-1H-pyrazol-4-yl)-1-tosyl-1H-pyrrolo[2,3-b]pyridine (25).

Anal. ($C_{29}H_{29}N_5O_3S$, 527.64) % found. (% calcd) C: 66.21 (66.01); H: 5.84 (5.54); N: 13.17 (13.27); S: 6.03 (6.08).

3-(3,5-dichlorophenyl)-5-(1-(piperidin-4-yl)-1H-pyrazol-4-yl)-1-tosyl-1H-pyrrolo[2,3-b]pyridine (26).

Anal. ($C_{28}H_{25}Cl_2N_5O_2S$, 566.50) % found. (% calcd) C: 59.64 (59.36); H: 4.58 (4.45); N: 11.99 (12.36); S: 5.47 (5.66).

3-(2-fluoro-5-methoxy-4-methylphenyl)-5-(1-(piperidin-4-yl)-1H-pyrazol-4-yl)-1-tosyl-1H-pyrrolo[2,3-b]pyridine (27).

Anal. ($C_{30}H_{30}FN_5O_3S$, 559.65) % found. (% calcd) C: 64.68 (64.38); H: 5.25 (5.40); N: 12.37 (12.51); S: 6.01 (5.73).

3-(3-fluorophenyl)-5-(1-(1-methylpiperidin-4-yl)-1H-pyrazol-4-yl)-1-tosyl-1H-pyrrolo[2,3-b]pyridine (28).

Anal. ($C_{29}H_{28}FN_5O_2S$, 529.63) % found. (% calcd) C: 65.98 (65.77); H: 5.66 (5.33); N: 13.12 (13.22); S: 5.99 (6.05).

3-(3-methoxyphenyl)-5-(1-(1-methylpiperidin-4-yl)-1H-pyrazol-4-yl)-1-tosyl-1H-pyrrolo[2,3-b]pyridine (29).

Anal. ($C_{30}H_{31}N_5O_3S$, 541.66) % found. (% calcd) C: 65.34 (65.52); H: 5.96 (5.77); N: 12.63 (12.93); S: 8.64 (8.92).

3-(3,5-dichlorophenyl)-5-(1-(1-methylpiperidin-4-yl)-1H-pyrazol-4-yl)-1-tosyl-1H-pyrrolo[2,3-b]pyridine (30).

Anal. ($C_{29}H_{27}C_{2}IN_{5}O_{2}S$, 580.53) % found. (% calcd) C: 60.34 (60.00); H: 5.06 (4.69); N: 12.03 (12.06); S: 5.64 (5.52).

3-(2-fluoro-5-methoxy-4-methylphenyl)-5-(1-(1-methylpiperidin-4-yl)-1H-pyrazol-4-yl)-1-tosyl-1H-pyrrolo[2,3-b]pyridine (31).

Anal. ($C_{31}H_{32}FN_{5}O_{3}S$, 573.68) % found. (% calcd) C: 64.60 (64.90); H: 6.00 (5.62); N: 11.97 (12.21); S: 5.61 (5.59).

HPLC chromatograms of compounds **1**, **5** – **11**.

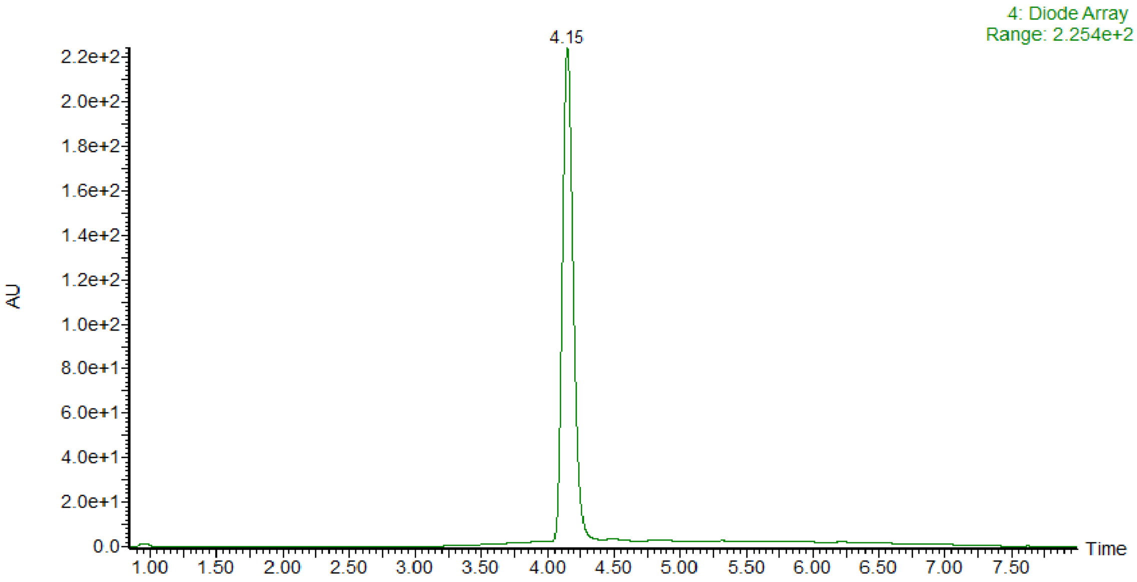

HPLC chromatogram of compound **1**.

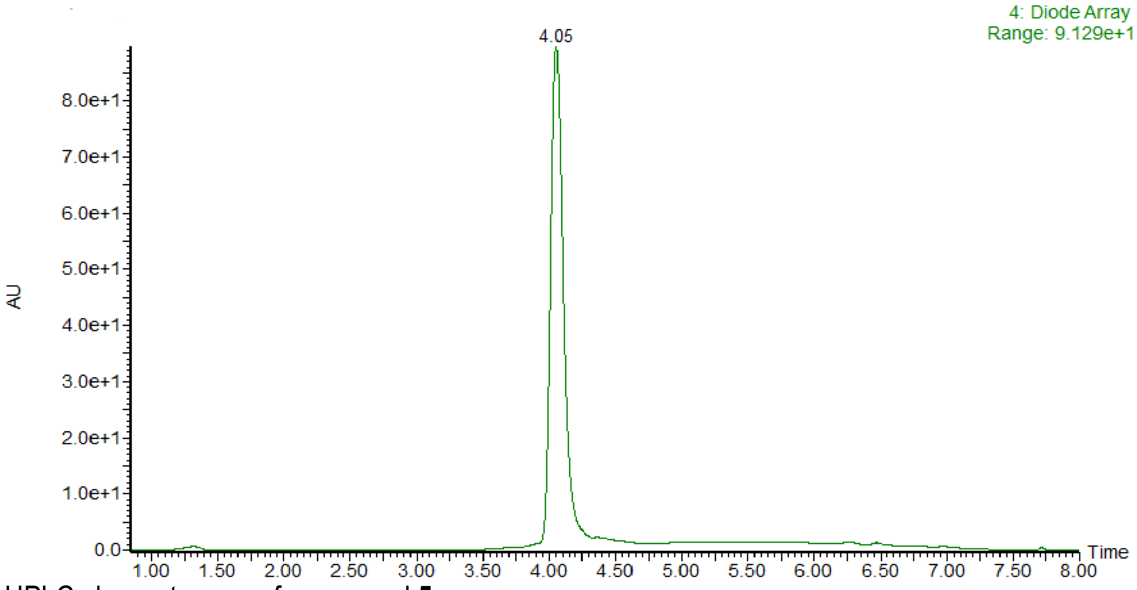

HPLC chromatogram of compound **5**.

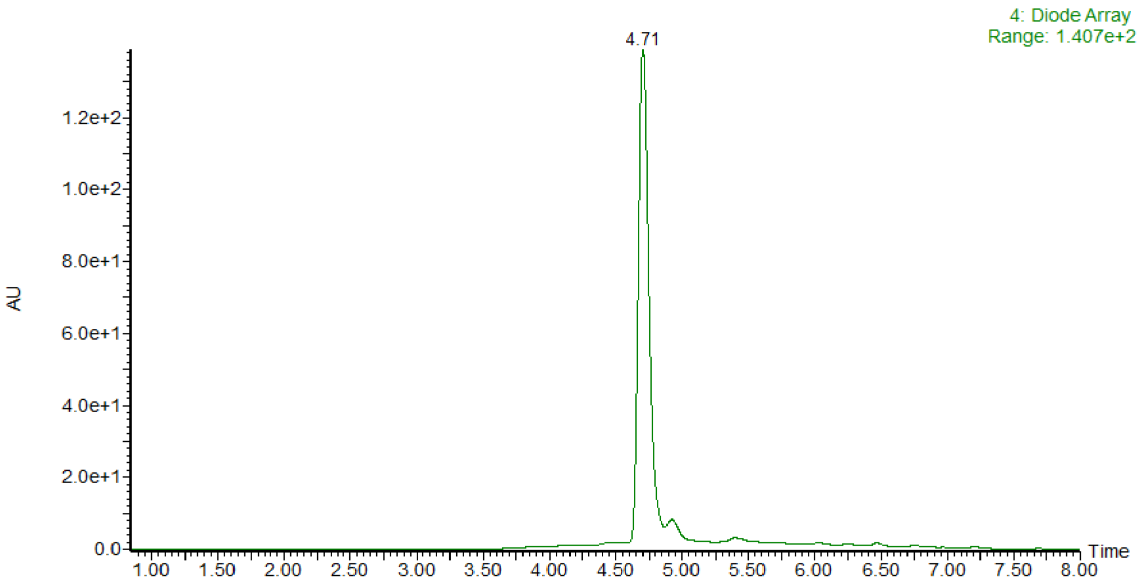

HPLC chromatogram of compound **6**.

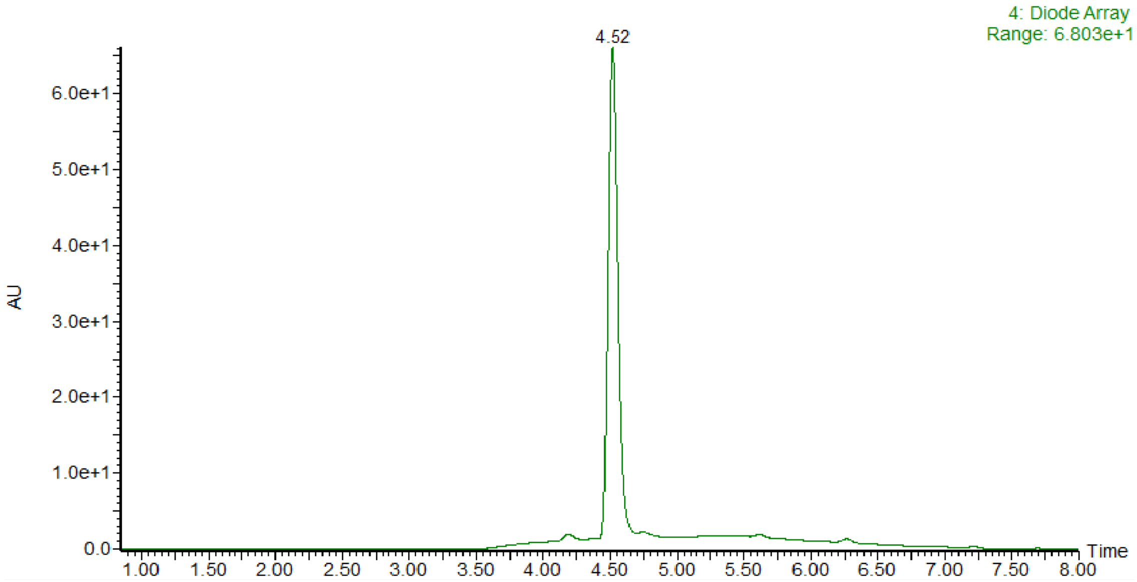

HPLC chromatogram of compound **7**.

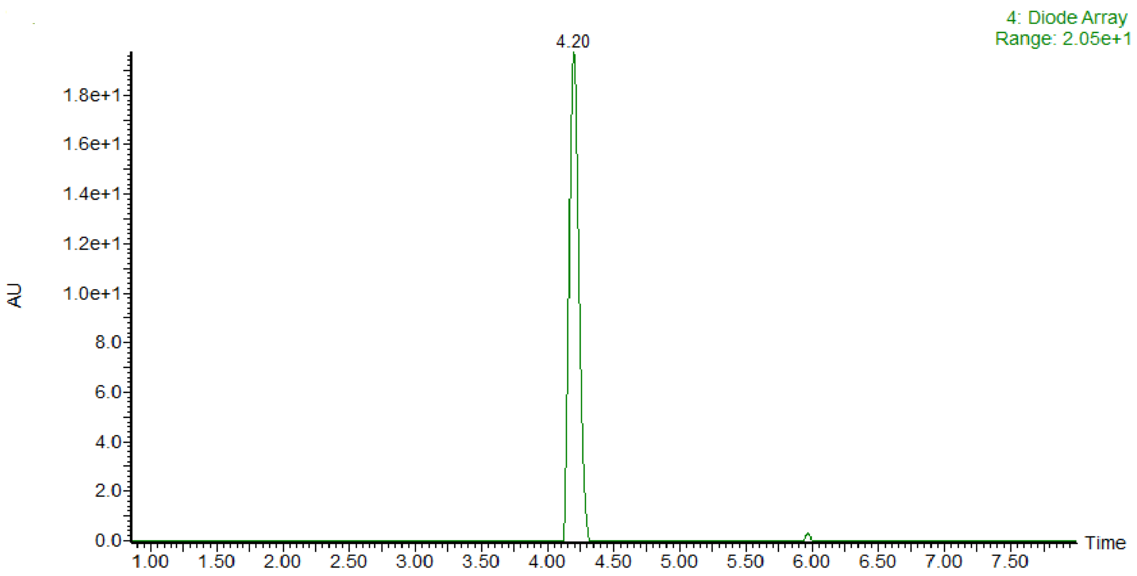

HPLC chromatogram of compound **8**.

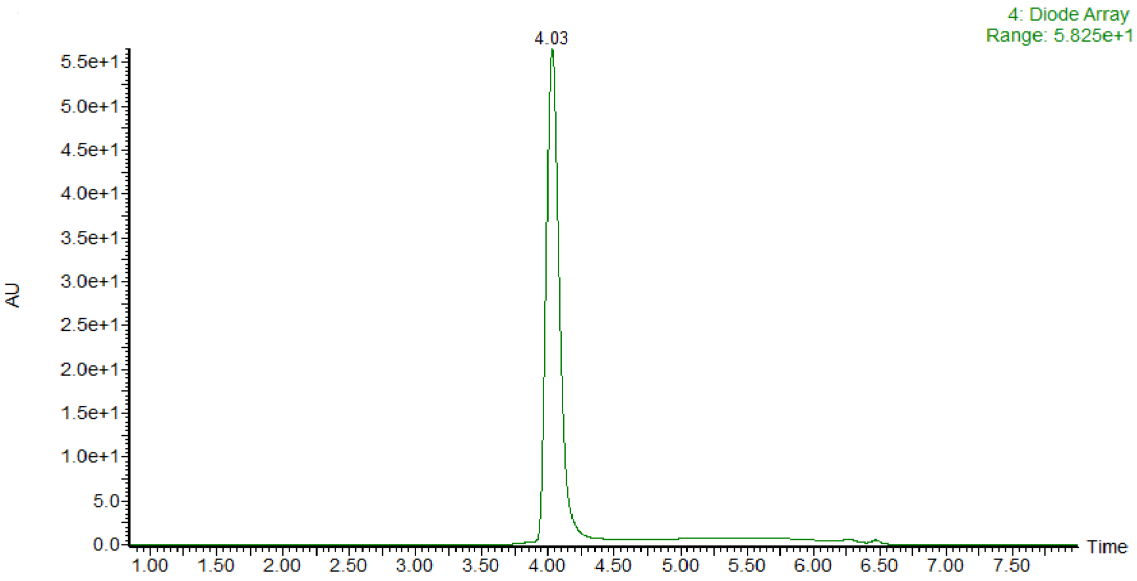

HPLC chromatogram of compound **9**.

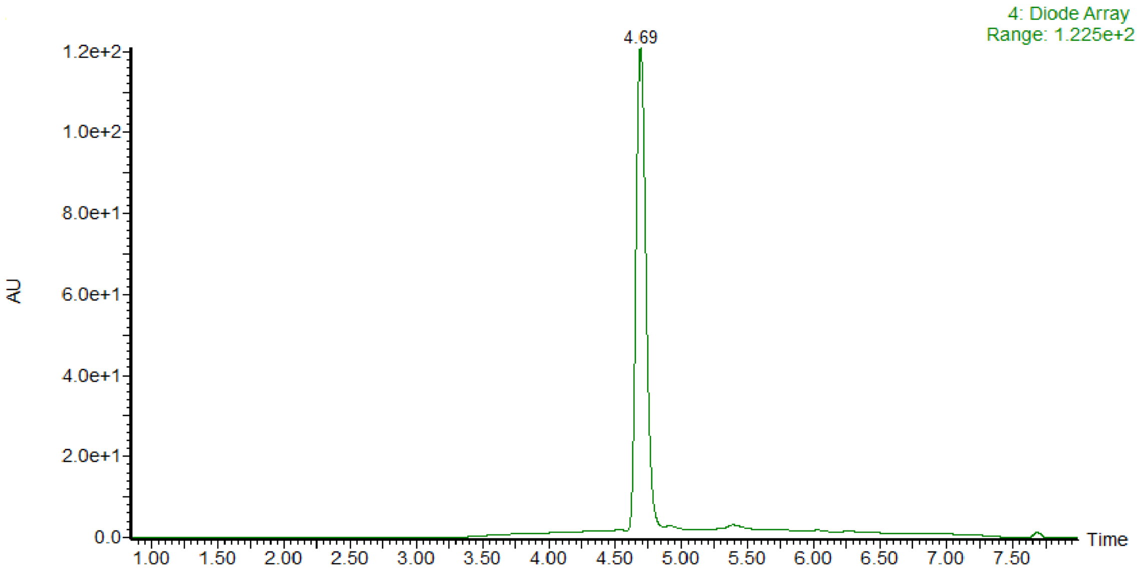

HPLC chromatogram of compound **10**.

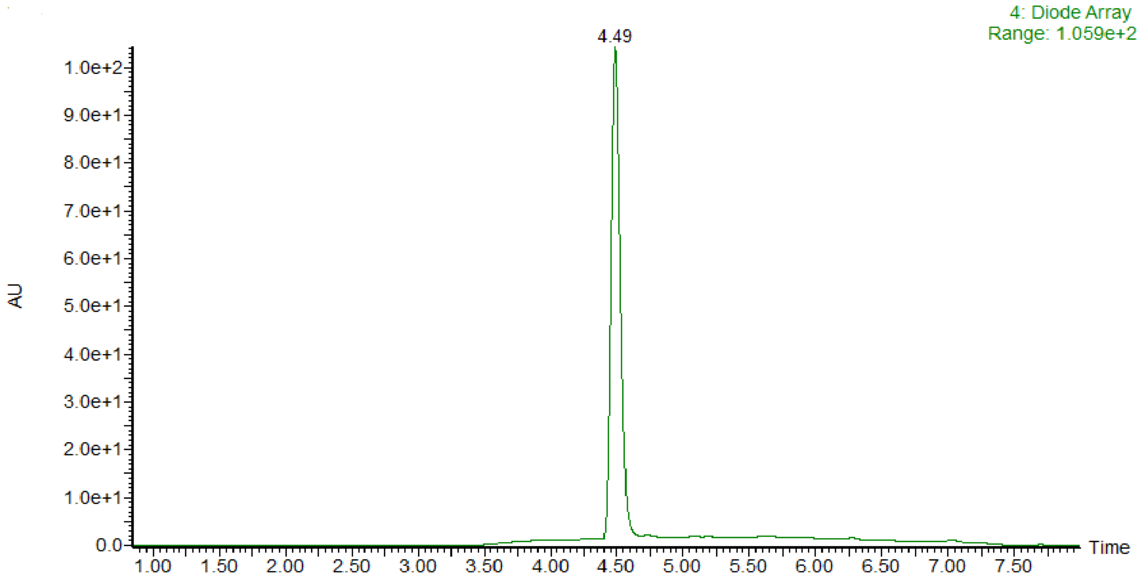

HPLC chromatogram of compound **11**.

Figures S17 – S18: Effect of compounds **1**, **5 - 11** in the nitrite production of BV2 Cells.

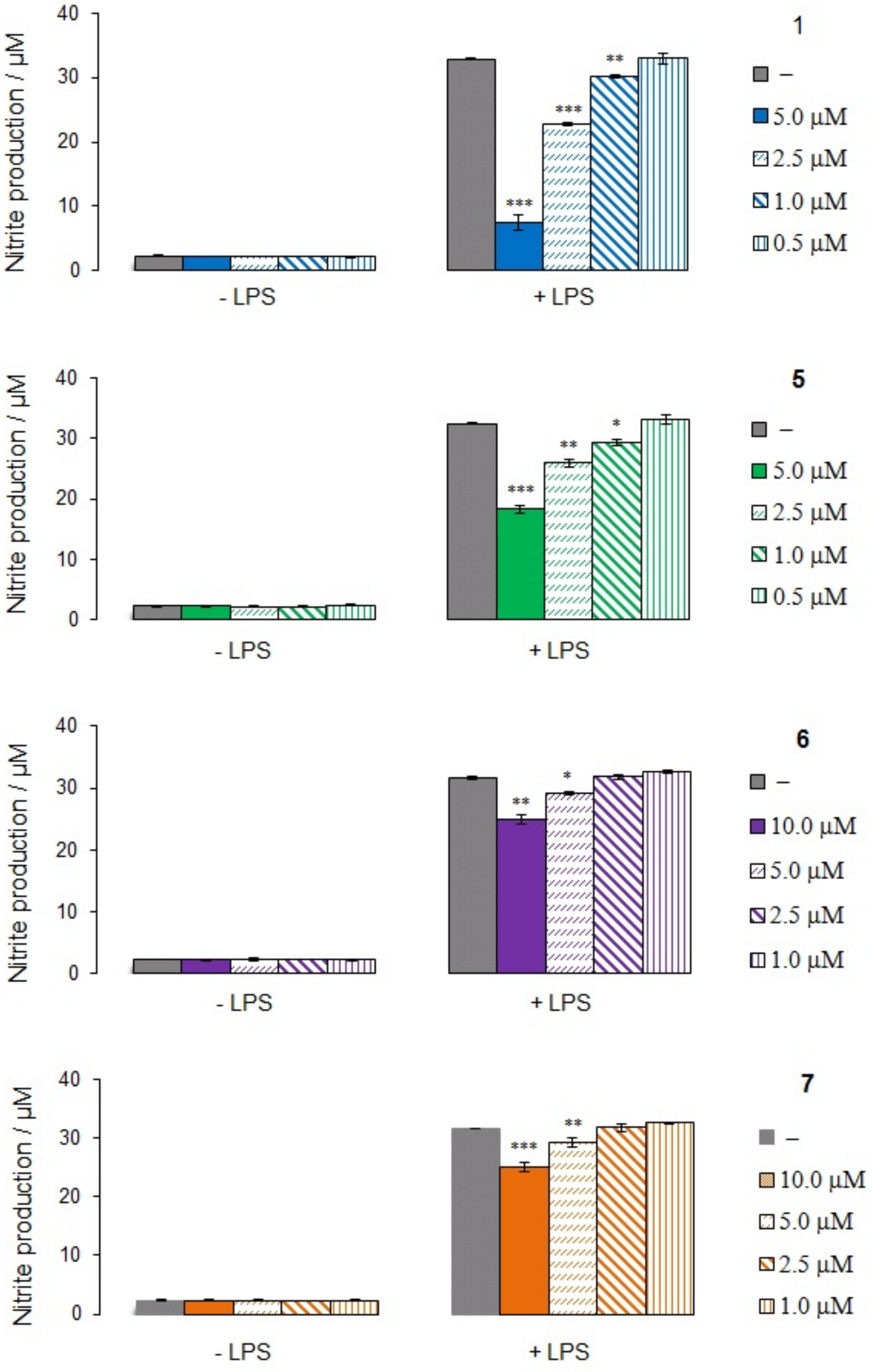

Figure S17. Effect of compounds **1**, **5** - **7** in the nitrite production of BV2 Cells. BV2 cells were incubated for 24 h with lipopolysaccharide (LPS; 200 ng mL−1) in the absence or presence of inhibitors at decreased concentrations, and the production of nitrite was evaluated by the Griess reaction. Cells were pretreated with inhibitors for 1 h before lipopolysaccharide (LPS) stimulation. Values represent the mean ± SD from three independent experiments. *: $p < 0.05$; **: $p < 0.01$; ***: $p < 0.01$ versus LPS-treated cells.

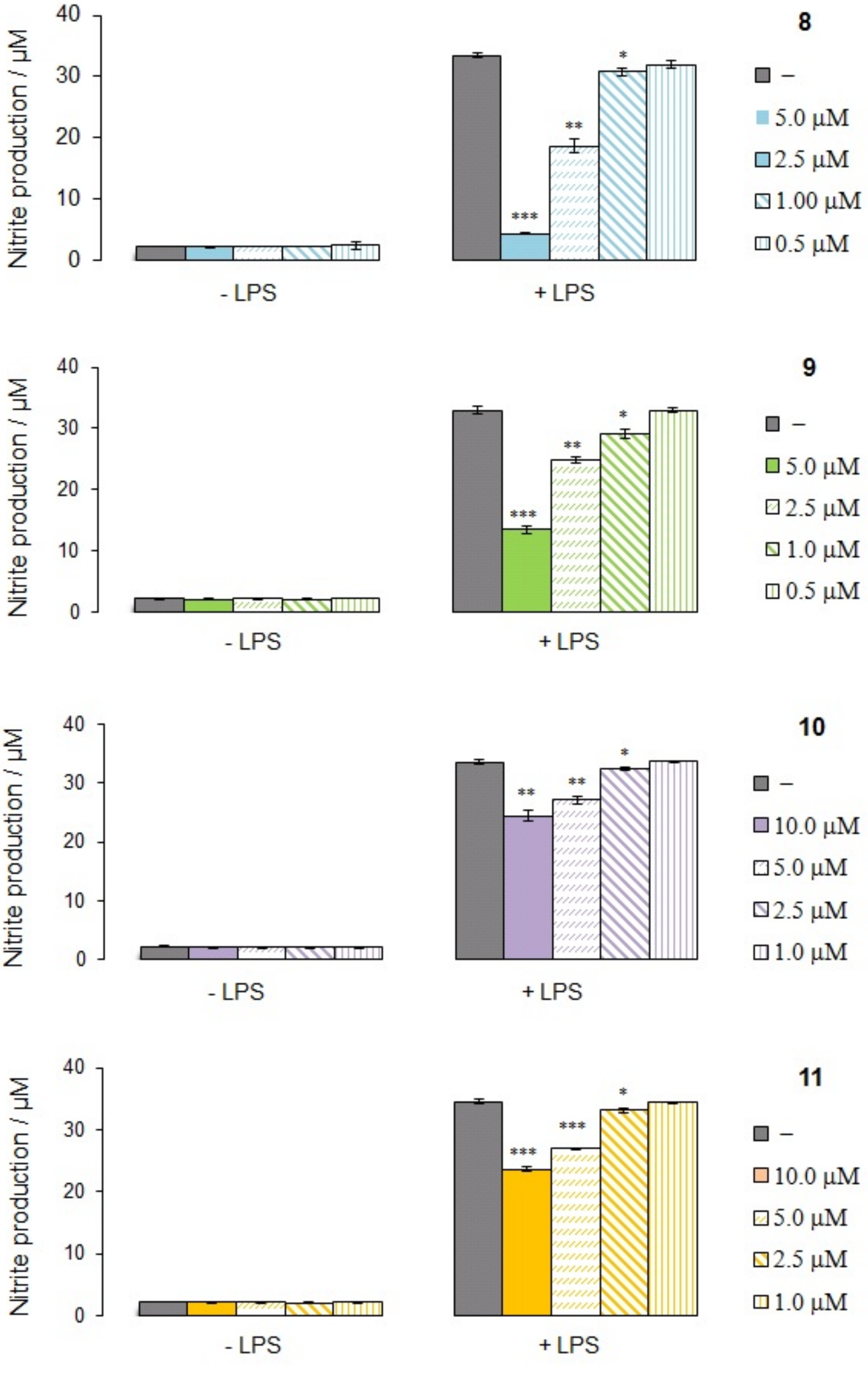

Figure S18. Effect of compounds **8** – **11** in the nitrite production of BV2 Cells. BV2 cells were incubated for 24 h with lipopolysaccharide (LPS; 200 ng mL−1) in the absence or presence of inhibitors at decreased concentrations, and the production of nitrite was evaluated by the Griess reaction. Cells were pretreated with inhibitors for 1 h before lipopolysaccharide (LPS) stimulation. Values represent the mean ± SD from three independent experiments. *: $p < 0.05$; **: $p < 0.01$; ***: $p < 0.01$ versus LPS-treated cells.

Figures S19-S20. Inhibitor dose-response curve for DYRK1A and DYRK1B

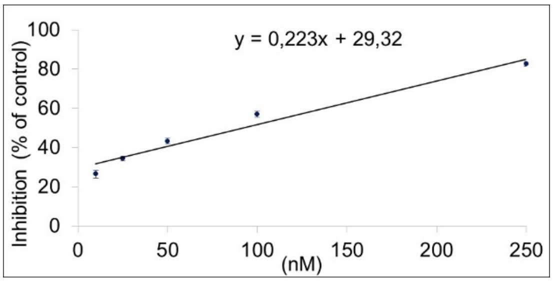

Harmine

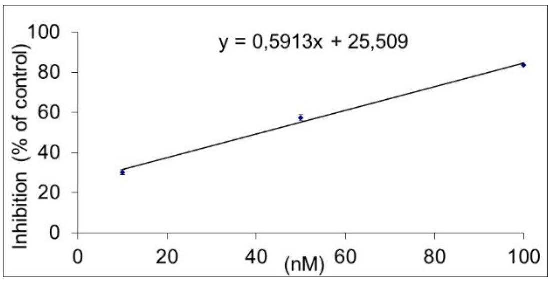

1

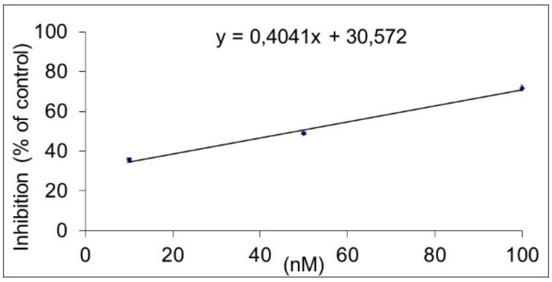

8

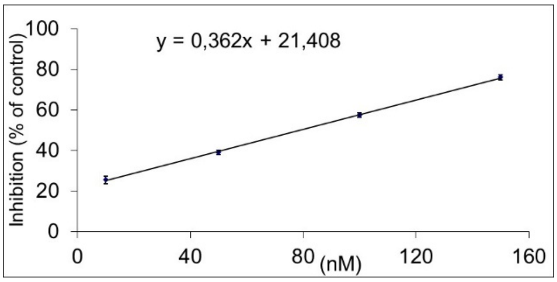

5

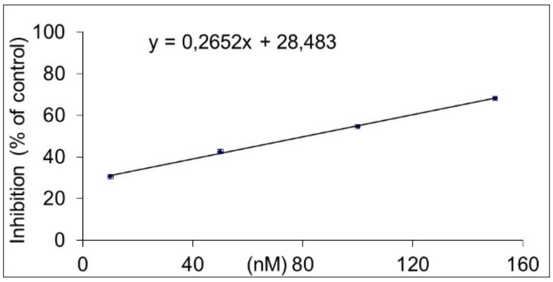

9

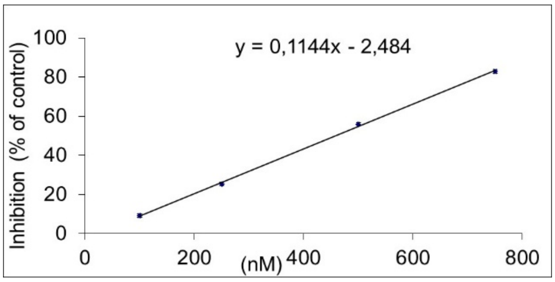

6

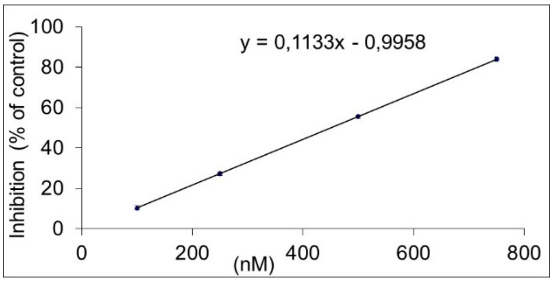

10

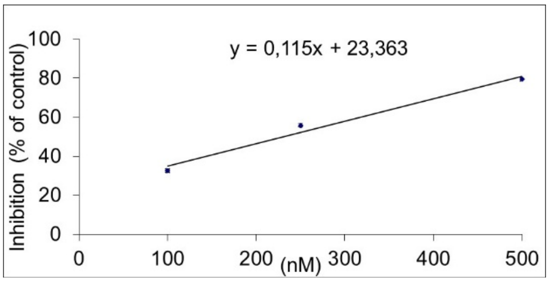

7

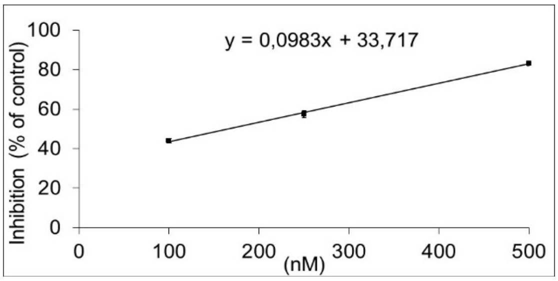

11

Figure S19. Inhibitor dose-response curve for DYRK1A IC_{50} determination of Harmine and compounds **1**, **5-11**.

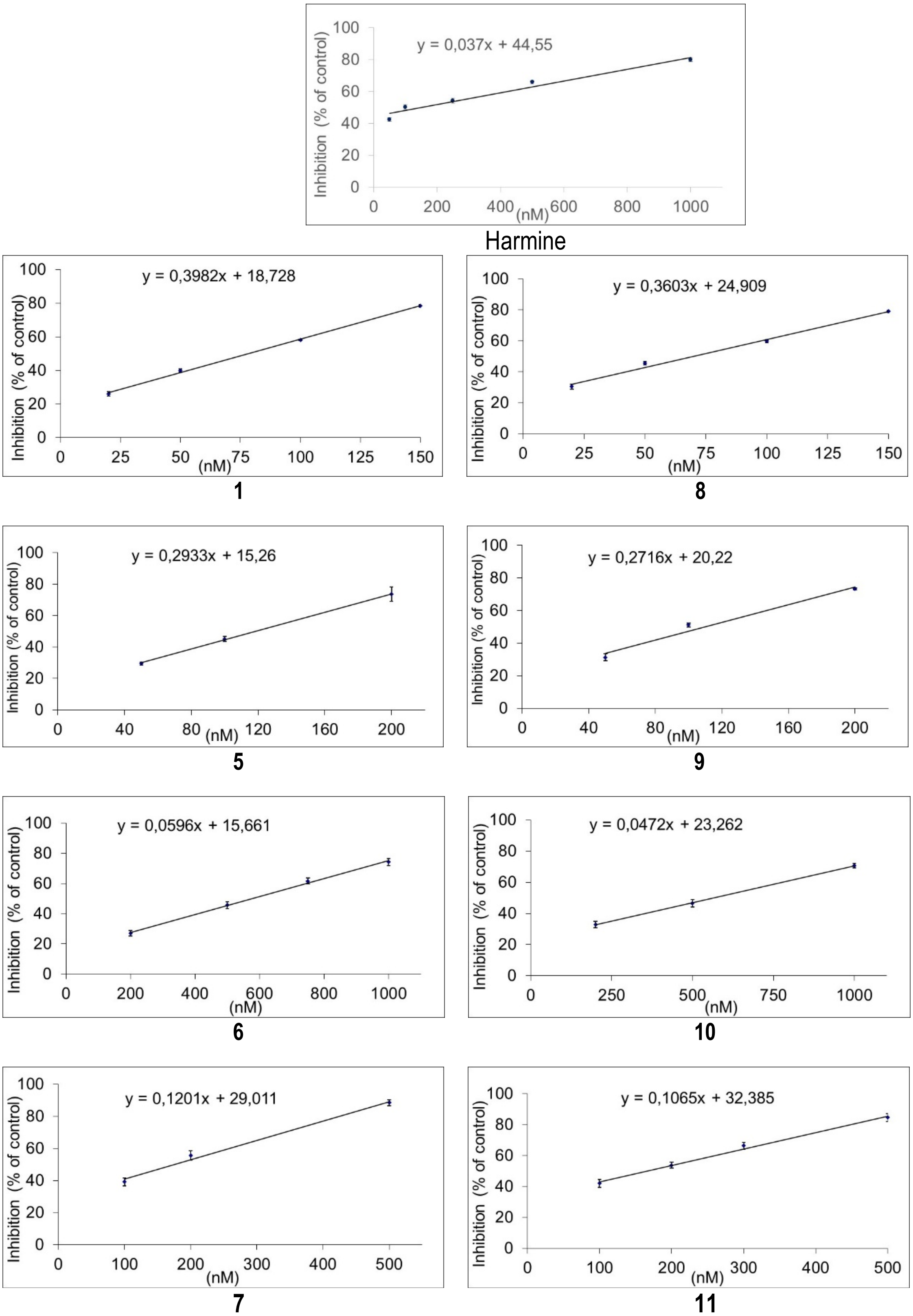

Figure S20. Inhibitor dose-response curve for DYRK1B IC_{50} determination of Harmine and compounds **1**, **5**-**11**.

Table S5. The descriptors used for ADMET/Tox analysis

Molecule	QPlogPo/w	QPlogHERG	QPPCaco	QPlogBB	QPPMDCK	QPlogKp	#metab	QPlogKhsa	%HOA	RuleOfFive
4E3	4,209	-3,459	207,821	-0,169	837,574	-2,585	0	0,247	93,07	0
I	4,45	-6,971	447,18	0,255	389,146	-3,904	2	1,026	100	0
5	4,334	-7,077	458,077	0,079	235,367	-3,849	3	0,988	100	0
6	5,13	-6,678	448,755	0,487	1296,522	-4,134	2	1,196	91,493	1
7	4,621	-6,388	444,657	0,182	336,224	-4,138	4	1,108	100	0
8	3,922	-6,895	269,179	0,036	237,551	-4,334	1	0,878	93,403	0
9	3,767	-6,787	270,503	-0,137	133,191	-4,322	2	0,85	92,532	0
10	4,557	-6,415	260,701	0,264	707,265	-4,606	1	1,045	96,873	0
11	4,088	-6,224	260,981	-0,04	189,138	-4,59	3	0,979	94,134	0
Range or recommended values	–2.0 – 6.5	< -5	<25 poor, >500 great	–3.0 – 1.2	<25 poor, >500 great	–8.0 – –1.0	1–8	–1.5 – 1.5	>80% is high, <25% is poor	maximum is 4

Docking studies

Table S6. Ligand interactions for redocked 4E3 ligand into 4YLL (HB: Hydrogen bond)

**Pose**	**RMSD (Å)**	**Docking Score (Kcal/mol)**	**Residue; Interaction, Distance (Å)**
1	0.715	-8.417	Lys167; Aromatic HB; 2.77
			Lys188; HB; 1.81
			Lys188; Salt bridge; 3.55
			Glu239; Aromatic HB; 2.39
			Leu241; Halogen Bond (Br); 2.78
2	0.739	-8.247	Lys167; Aromatic HB; 2.76
			Lys188; HB; 1.87
			Lys188; Salt bridge; 3.61
			Glu239; Aromatic HB; 2.35
			Leu241; Halogen Bond (Br); 2.70

DFT studies

Morfeus descriptors

Descriptors Definitions

Solvent-Accessible Surface Area (SASA):

The solvent-accessible surface area (SASA) measures how much of the molecular surface is available to interact with a solvent. It is calculated using a modified version of the Shrake and Rupley method, which employs a constant density of points on the molecular surface instead of a fixed number of points regardless of atom size. A probe radius of 1.4 Å is conventionally used, representing water.

Sterimol Parameters (Sterimol B1, B5, and Boltzmann-Averaged Sterimol B1 and B5):

- *Sterimol B1*: The smallest width of a substituent measured perpendicular to the bond axis connecting the substituent to the core structure.
- *Sterimol B5*: The maximum width of a substituent measured perpendicular to the bond axis.
- *Boltzmann-weighted Sterimol B1/B5*: The Boltzmann-weighted average of Sterimol B1 and B5 values over all conformers at room temperature (298.15 K).

Emin (Minimum Molecular Electrostatic Potential, Vmin):
Emin represents the lowest electrostatic potential in the phosphorus lone pair region. It is an indicator of the

electron-deficient regions in a Compound and is crucial in assessing the molecular reactivity, particularly in electrophilic and nucleophilic interactions.

Boltz (Boltzmann-Weighted Average):

The Boltzmann-weighted average (*Boltz*) is used to condense molecular descriptor data by averaging values across all conformers, weighted according to their relative Boltzmann population. This approach provides a more physically meaningful representation of a Compound's steric and electronic properties by incorporating thermal contributions at 298.15 K**.**

Compound	Pyr_Angle	Dipole	Sterimol_B1	Sterimol_B5	SASA_Area
**1**	0.04	1.93	2.70	10.24	601.12
**5**	0.08	1.19	2.58	10.43	629.66
**6**	0.08	2.12	**3.28**	**11.74**	**646.19**
**7**	0.10	1.32	3.21	10.44	**663.32**

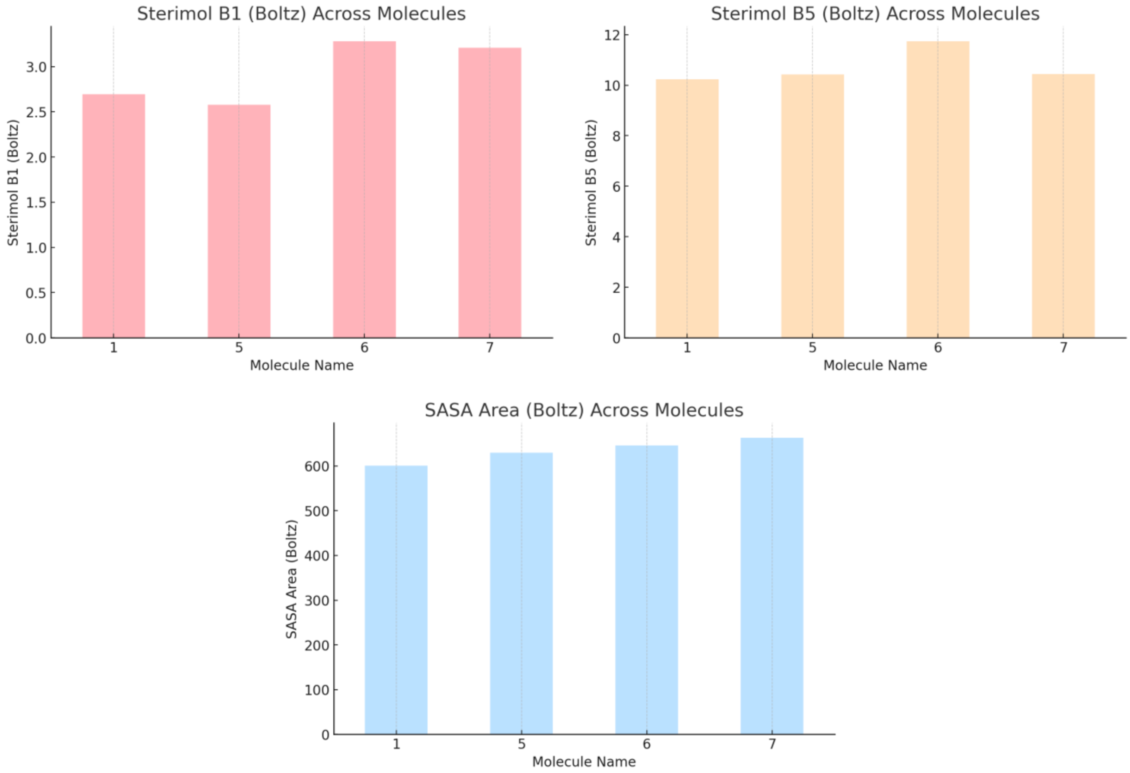

Figure S21. Bar chart comparison of Steric descriptors for Compounds **1**, **5**, **6** and **7**.

DFT analysis

Geometric conformation of compounds **1**, **5**, and **7** can be easily reproduced by optimizing their geometry using DFT calculations. As observed in the following image, the phenyl ring in Compound 1 adopts a

minimum-energy conformation in which the ring rotates, separating protons H1 and H2. This rotation occurs due to steric repulsion between these two atoms.

A similar effect is observed in derivatives **5** and **6,** as there are no substituents in the ortho position of the phenyl ring to restrict rotation. In contrast, Compound 7 contains a fluorine atom in the ortho position. Although fluorine is not a highly bulky atom, the minimum-energy conformation involves orienting the fluorine as far away as possible from the most steric stress point.

Additionally, the steric hindrance between H1 and H2, combined with the steric interactions between fluorine and H3, results in the geometry previously obtained during the docking study.

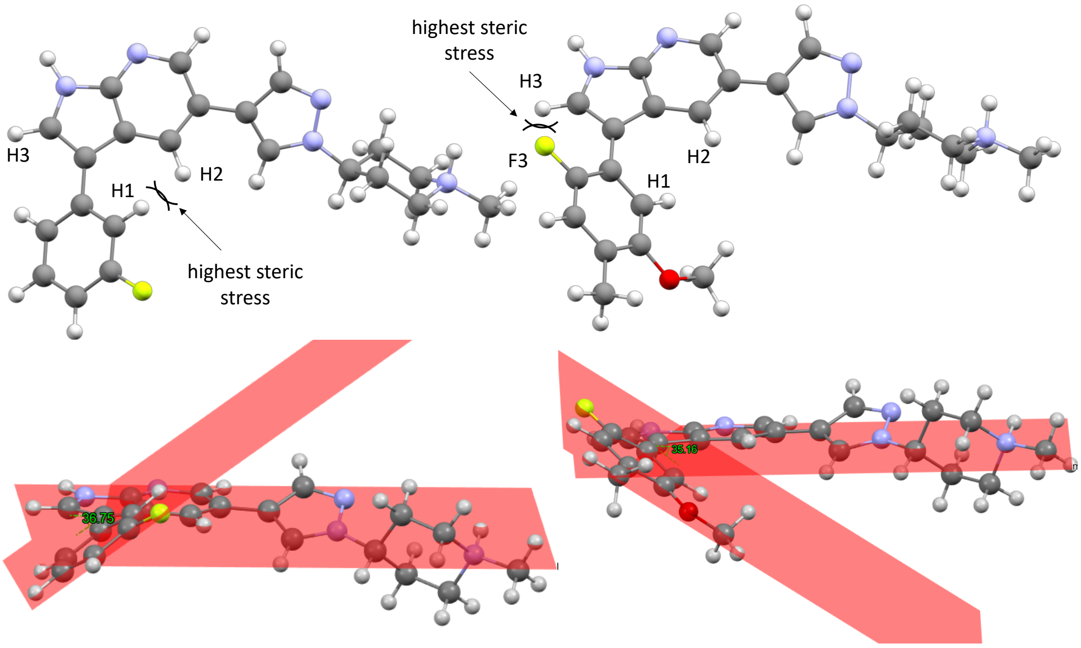

Figure S22. Optimized geometries of **1** and **7**. Planes were generated using Mercury Software. Angles are given in degrees.

Mulliken Charges Comparison

Below is a comparison of Mulliken charges for selected atoms across the Compounds:

Atom Type	Compound 1	Compound 6	Compound 7
**N (Pyrrole)**	-0.152	-0.149	-0.151
**N (Pyridine)**	-0.218	-0.206	-0.214
**H (N-H Bond)**	0.321	0.323	0.320
**C (Adjacent to N)**	0.266	0.235	0.305
**C (Aromatic Core 1)**	-0.579	-0.582	-0.475
**C (Aromatic Core 2)**	0.455	0.498	0.586
**C (Aromatic Core 3)**	-0.061	-0.244	-0.166
**C (Extended System)**	-0.210	0.102	-0.166

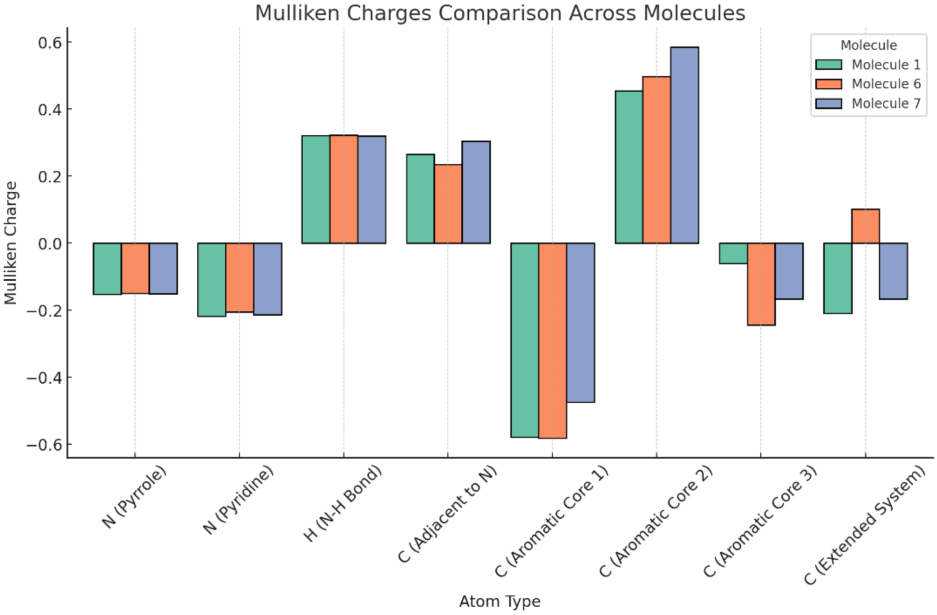

Figure S23. Bar chart comparison of Mulliken charges for compounds **1**, **6** and **7**.

The analysis of Mulliken charges across the studied Compounds reveals distinct electronic differences, particularly in Compound 7. While Compounds **1** and **6** share similar charge distributions, Compound **7** exhibits notable deviations, especially in the pyridine nitrogen (N14), adjacent carbon (C15), and aromatic core (C17-C19). The increased positive charge on C15 and the decreased negative charge on N14 suggest a weaker electron-donating ability of the nitrogen lone pair in Compound **7**, which could influence its reactivity and interaction potential. These differences in Mulliken charges can be explained by a higher electron density donated by the attached phenyl group. Having three functional groups ultimately imparts a certain donor character, resulting in a different electron density distribution in this Compound.

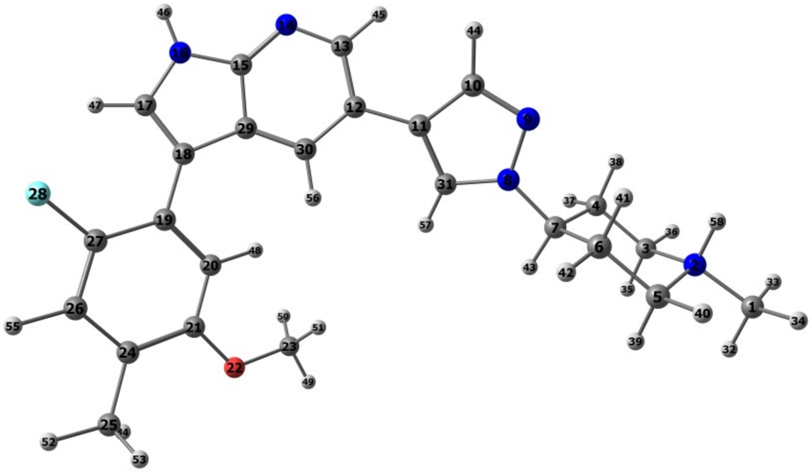

Figure S24. Optimized geometries **7**. Atom labels have been included.

Natural Bond Orbital analysis.

Interaction	Compound 1	Compound 6	Compound 7

**LP (Npyrazole)⟶ BD*(π-system)**	10.34	9.40	7.35
**LP (Npy) ⟶ BD*(π-system)**	35.13	35.66	34.34
**LP (Npy)⟶ BD*(π-system)**	49.84	37.74	33.80

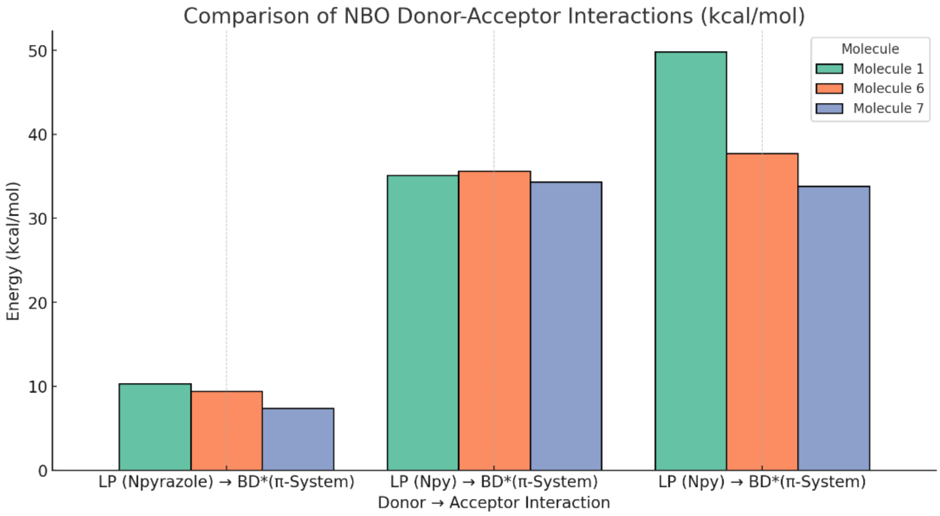

Figure S25. Bar chart comparison of NBO donations for compounds **1, 6** and **7**.

- The higher LP(N) ⟶ Aromatic System interaction in 1 means greater nitrogen electron delocalization, making the N-H bond more electropositive and favoring hydrogen bonding.
- Compound **7**, with lower donation (7.35 kcal/mol), retains more electron density on nitrogen, reducing the electropositivity of the N-H bond, and weakening the hydrogen bonding interaction.
- Compound **6** (9.4 kcal/mol) is between these cases, indicating that its hydrogen bonding potential might also be weaker than Compound **1** but stronger than Compound **7.**

Calculated coordinates

Compound 1

6 8.017498000 1.672773000
0.232709000
7 6.743746000 0.899043000
0.044923000
6 5.699080000 1.688856000 -
0.727912000
6 4.465973000 0.831968000 -
0.991370000
6 3.862435000 0.267007000
0.309248000
7 2.762692000 -0.627342000
0.006772000
6 1.441727000 -0.498959000
0.329227000
6 0.782590000 -1.596112000 -
0.208887000
6 -0.652200000 -1.907414000 -
0.144560000
6 -1.087577000 -3.256868000 -
0.169770000
7 -2.363768000 -3.641427000 -
0.108137000
6 -3.246438000 -2.649039000 -
0.022406000
7 -4.609850000 -2.794515000
0.072352000
6 -5.191796000 -1.545512000
0.152090000
6 -4.224019000 -0.560884000
0.111399000
6 -4.448065000 0.889941000
0.178157000
6 -5.461116000 1.434690000
0.989835000
6 -5.674661000 2.813348000
1.037675000
6 -4.874619000 3.688564000
0.295653000
6 -3.875098000 3.133309000 -
0.493501000
9 -3.083265000 3.962673000 -
1.229466000
6 -3.647923000 1.766843000 -
0.577478000
6 -2.960670000 -1.252554000
0.001493000
6 -1.612172000 -0.890598000 -
0.056110000
6 1.819382000 -2.334442000 -
0.844738000
7 3.008653000 -1.748051000 -
0.715195000
6 4.943829000 -0.468121000
1.123285000
6 6.171624000 0.403227000
1.363830000
1 7.798588000 2.560514000
0.827726000
1 8.742860000 1.044746000
0.751774000
1 8.404769000 1.964424000 -
0.744507000
1 6.168397000 2.023913000 -
1.655957000

1 5.474767000 2.567131000 -
0.114274000
1 3.726701000 1.442793000 -
1.518500000
1 4.713880000 -0.006354000 -
1.653026000
1 3.449023000 1.087891000
0.907211000
1 1.077892000 0.330565000
0.918805000
1 -0.354162000 -4.058076000 -
0.223661000
1 -5.091706000 -3.681075000
0.053263000
1 -6.265782000 -1.440093000
0.207878000
1 -6.068166000 0.773102000
1.599995000
1 -6.461876000 3.215069000
1.668489000

Compound 5

6 8.092714000 1.829607000
0.362381000
7 6.850935000 1.020156000
0.120229000
6 5.781002000 1.812619000 -
0.614483000
6 4.584331000 0.925354000 -
0.938230000
6 3.994806000 0.262171000
0.321625000
7 2.935845000 -0.657147000 -
0.042739000
6 1.614905000 -0.624248000
0.304582000

1 -5.013955000 4.763503000
0.321290000
1 -2.884850000 1.397173000 -
1.252733000
1 -1.311870000 0.152577000 -
0.045684000
1 1.735361000 -3.257776000 -
1.401595000
1 5.220278000 -1.384527000
0.588880000
1 4.543667000 -0.777153000
2.093776000
1 6.966348000 -0.145504000
1.874776000
1 5.939791000 1.301630000
1.944676000
1 6.962427000 0.067384000 -
0.515628000

6 1.008562000 -1.719486000 -
0.295520000
6 -0.404308000 -2.118025000 -
0.229574000
6 -0.757446000 -3.487928000 -
0.326909000
7 -2.006324000 -3.953658000 -
0.265637000
6 -2.945231000 -3.023780000 -
0.105143000
7 -4.294162000 -3.258001000
0.006039000
6 -4.948166000 -2.052219000
0.170566000

6 -4.043303000 -1.009316000
0.169883000
6 -4.358081000 0.419007000
0.326058000
6 -5.388791000 0.840176000
1.191055000
6 -5.690442000 2.193720000
1.308551000
6 -4.975726000 3.162805000
0.592636000
6 -3.944511000 2.748640000 -
0.260177000
6 -3.644759000 1.385867000 -
0.393876000
6 -2.743901000 -1.615342000 -
0.004051000
6 -1.421564000 -1.168142000 -
0.065245000
6 2.074343000 -2.356629000 -
0.990330000
7 3.232064000 -1.715213000 -
0.836976000
6 5.099417000 -0.475082000
1.102584000
6 6.290431000 0.427602000
1.403801000
1 7.836319000 2.672587000
1.005576000
1 8.838632000 1.200846000
0.850505000
1 8.474364000 2.191630000 -
0.593280000
1 6.242994000 2.219324000 -
1.517250000

1 5.518537000 2.644030000
0.047434000
1 3.824844000 1.536448000 -
1.435457000
1 4.869147000 0.137398000 -
1.645304000
1 3.543659000 1.029538000
0.961896000
1 1.214709000 0.144544000
0.950247000
1 0.022075000 -4.237871000 -
0.440439000
1 -4.721950000 -4.169829000 -
0.055250000
1 -6.024870000 -2.016113000
0.254041000
1 -5.932242000 0.106612000
1.778319000
1 -5.228641000 4.209554000
0.708212000
1 -2.876077000 1.099025000 -
1.103323000
1 -1.186920000 -0.110114000 -
0.000762000
1 2.031741000 -3.245299000 -
1.605252000
1 5.415434000 -1.347655000
0.518942000
1 4.704861000 -0.854980000
2.049946000
1 7.102878000 -0.118339000
1.889270000
1 6.019892000 1.281532000
2.033005000

1 7.104585000 0.231093000 -0.485380000
1 -6.486559000 2.511604000 1.975808000
8 -3.174875000 3.594055000 -1.013361000
6 -3.487589000 4.983451000 -1.004892000
1 -3.355785000 5.418990000 -0.005681000
1 -2.787215000 5.451182000 -1.698470000
1 -4.514060000 5.163928000 -1.348625000

Compound 6

6 -8.378672000 -2.324616000 0.482606000
7 -7.219846000 -1.396754000 0.252696000
6 -6.135515000 -2.031610000 -0.603976000
6 -5.030026000 -1.025288000 -0.903435000
6 -4.423047000 -0.430792000 0.381950000
7 -3.454761000 0.596936000 0.053808000
6 -2.116871000 0.629816000 0.325143000
6 -1.623490000 1.815429000 -0.202814000
6 -0.238105000 2.305209000 -0.178449000
6 0.022020000 3.699516000 -0.171341000
7 1.240551000 4.241802000 -0.137095000
6 2.244083000 3.368262000 -0.113289000
7 3.581241000 3.683980000 -0.054298000
6 4.319226000 2.519419000 -0.033751000
6 3.482264000 1.421242000 -0.080739000
6 3.888657000 0.009674000 -0.071910000
6 4.983379000 -0.417554000 0.699623000
6 5.358113000 -1.759667000 0.686018000
6 4.661109000 -2.713338000 -0.059306000
6 3.572677000 -2.272972000 -0.812176000
6 3.183663000 -0.935717000 -0.838027000
6 2.138138000 1.947299000 -0.129611000
6 0.845233000 1.417240000 -0.156601000
6 -2.769433000 2.432114000 -0.777876000
7 -3.868997000 1.696022000 -0.622903000
6 -5.534796000 0.145883000 1.278825000

6 -6.634777000 -0.873115000 1.554984000
1 -8.020509000 -3.195972000 1.032612000
1 -9.142051000 -1.804505000 1.062898000
1 -8.785229000 -2.632814000 -0.481609000
1 -6.615063000 -2.393370000 -1.516629000
1 -5.773136000 -2.894129000 -0.035670000
1 -4.255798000 -1.527367000 -1.491653000
1 -5.413471000 -0.203906000 -1.520082000
1 -3.882245000 -1.213569000 0.926942000
1 -1.628186000 -0.161481000 0.875722000
1 -0.808377000 4.401702000 -0.174200000
1 3.945894000 4.625199000 -0.055595000
1 5.399276000 2.550471000 -0.012747000
1 5.524081000 0.286743000 1.321544000
1 4.956018000 -3.755266000 -0.052339000
1 2.364490000 -0.626046000 -1.475575000
1 0.679529000 0.344453000 -0.172497000
1 -2.824843000 3.372400000 -1.309389000
1 -5.948282000 1.037206000 0.792571000
1 -5.118461000 0.472921000 2.236542000
1 -7.457807000 -0.438622000 2.127318000
1 -6.264591000 -1.753003000 2.090706000
1 -7.569208000 -0.582256000 -0.265225000
17 2.672208000 -3.442517000 -1.768388000
17 6.728243000 -2.276382000 1.650496000

Compound 7

6 8.379725000 1.980294000 -0.293991000
7 7.161596000 1.101686000 -0.321318000
6 6.624099000 0.819042000 1.072804000
6 5.459665000 -0.163057000 1.010203000
6 6.065481000 1.664480000 -1.212423000
6 4.894586000 0.692155000 -1.301011000
6 4.329902000 0.332230000 0.087140000
7 3.300649000 -0.680627000 -0.036619000

7 3.633297000 -1.888965000 -
0.553087000
6 2.495884000 -2.581959000 -
0.552107000
6 1.405066000 -1.828527000 -
0.034359000
6 0.004021000 -2.247107000
0.110001000
6 -0.303118000 -3.615827000
0.316833000
7 -1.539947000 -4.106547000
0.413415000
6 -2.512280000 -3.202291000
0.327428000
7 -3.859057000 -3.470164000
0.352974000
6 -4.558347000 -2.290061000
0.207005000
6 -3.684896000 -1.225116000
0.098818000
6 -4.030799000 0.193593000 -
0.049367000
6 -3.240005000 1.199743000
0.547449000
6 -3.549231000 2.551890000
0.398481000
8 -2.820822000 3.569283000
0.964306000
6 -1.737048000 3.237604000
1.815996000
6 -4.667072000 2.965804000 -
0.358072000
6 -4.986077000 4.430284000 -
0.504263000

6 -5.455973000 1.973054000 -
0.938961000
6 -5.138078000 0.629377000 -
0.777638000
9 -5.953291000 -0.291821000 -
1.368958000
6 -2.357626000 -1.793234000
0.164158000
6 -1.047660000 -1.323519000
0.031162000
6 1.974922000 -0.603866000
0.286960000
1 8.100896000 2.945298000
0.131534000
1 9.146188000 1.507906000
0.321939000
1 8.746248000 2.113238000 -
1.312800000
1 6.330861000 1.791143000
1.481862000
1 7.454214000 0.428507000
1.666248000
1 5.080795000 -0.315700000
2.025413000
1 5.799925000 -1.140803000
0.649618000
1 5.781216000 2.622806000 -
0.766223000
1 6.511074000 1.855592000 -
2.191566000
1 5.200383000 -0.233627000 -
1.802347000
1 4.115271000 1.144608000 -
1.921860000

1 3.855840000 1.216256000

0.530063000

1 2.483834000 -3.592714000 -

0.936556000

1 0.502354000 -4.340495000

0.410968000

1 -4.255776000 -4.393351000

0.446809000

1 -5.637558000 -2.284026000

0.190953000

1 -2.404266000 0.890115000

1.161203000

1 -1.336195000 4.187115000

2.175026000

1 -2.066596000 2.634436000

2.672570000

1 -0.950240000 2.693223000

1.272799000

1 -5.879022000 4.578056000 -

1.116839000

1 -4.152076000 4.969898000 -

0.966824000

1 -5.153318000 4.894150000

0.474176000

1 -6.331065000 2.233927000 -

1.525603000

1 -0.848242000 -0.275027000 -

0.169642000

1 1.548195000 0.286106000

0.727148000

1 7.436645000 0.197895000 -

0.722928000

References

NBO = Glendening, E. D.; Landis, C. R.; Weinhold, F. NBO 7.0: New Features and Capabilities. *J. Comput. Chem.* 2019, *40*, 2234–2241.

Frisch, M. J.; Trucks, G. W.; Schlegel, H. B.; Scuseria, G. E.; Robb, M. A.; Cheeseman, J. R.; Scalmani, G.; Barone, V.; Petersson, G. A.; Nakatsuji, H.; Li, X.; Caricato, M.; Marenich, A. V.; Bloino, J.; Janesko, B. G.; Mennucci, B.; Hratchian, H. P.; Ortiz, J. V.; Izmaylov, A. F.; Sonnenberg, J. L.; Williams-Young, D.; Ding, F.; Lipparini, F.; Egidi, F.; Goings, J.; Peng, B.; Petrone, A.; Henderson, T.; Ranasinghe, D.; Zakrzewski, V. G.; Gao, J.; Rega, N.; Liang, W.; Hada, M.; Ehara, M.; Toyota, K.; Fukuda, R.; Hasegawa, J.; Ishida, M.; Nakajima, T.; Honda, Y.; Kitao, O.; Nakai, H.; Vreven, T.; Throssell, K.; Montgomery, J. A., Jr.; Peralta, J. E.; Ogliaro, F.; Bearpark, M. J.; Heyd, J. J.; Brothers, E. N.; Kudin, N.; Staroverov, V. N.; Keith, T. A.; Kobayashi, R.; Normand, J.; Raghavachari, K.; Rendell, A. P.; Burant, J. C.; Iyengar, S. S.; Tomasi, J.; Cossi, M.; Millam, J. M.; Klene, M.; Adamo, C.; Cammi, R.; Ochterski, J. W.; Martin, R. L.; Morokuma, K.; Farkas, O.; Foresman, J. B.; Fox, D. J. Gaussian 16, Revision E.01; Gaussian, Inc.: Wallingford, CT, 2009.

Marenich, A. V.; Cramer, C. J.; Truhlar, D. G. Universal Solvation Model Based on Solute Electron Density and on a Continuum Model of the Solvent Defined by the Bulk Dielectric Constant and Atomic Surface Tensions. *J. Phys. Chem. B* **2009**, *113*, 6378–6396.

(a) Lee, C.; Yang, W.; Parr, R. G. Development of the Colle-Salvetti Correlation-Energy Formula into a Functional of the Electron Density. *Phys. Rev. B* **1988**, *37*, 785–789.
(b) Miehlich, B.; Savin, A.; Stoll, H.; Preuss, H. Results Obtained with the Correlation Energy Density Functionals of Becke and Lee, Yang and Parr. *Chem. Phys. Lett.* **1989**, *157* , 200–206.
(c) Becke, A. D. Density-Functional Thermochemistry. III. The Role of Exact Exchange. *J. Chem. Phys.* **1993**, *98*, 5648–5652.

Grimme, S.; Antony, J.; Ehrlich, S.; Krieg, H. A Consistent and Accurate *ab initio* Parametrization of Density Functional Dispersion Correction (DFT-D) for the 94 Elements H-Pu. *J. Chem. Phys.* **2010**, *132*, 154104.

Hehre, W. J.; Ditchfield, R.; Pople, J. A. Self-Consistent Molecular Orbital Methods. XII. Further Extensions of Gaussian-Type Basis Sets for Use in Molecular Orbital Studies of Organic Compounds. *J. Chem. Phys.* **1972**, *56*, 2257–2261.

Hehre, W. J.; Pietro, W. J.; Binkley, J. S.; Gordon, M. S.; DeFrees, D. J.; Pople, J. A. Self-Consistent Molecular Orbital Methods. XV. Extended Gaussian-Type Basis Sets for Molecular Orbital Studies of Organic Compounds. *J. Chem. Phys.* **1982**, *77*, 3654–3665.
